# Microbiota composition data of imago and larval stage of the anhydrobiotic midge

**DOI:** 10.1016/j.dib.2020.106527

**Published:** 2020-11-18

**Authors:** Nurislam Shaikhutdinov, Natalia Gogoleva, Oleg Gusev, Elena Shagimardanova

**Affiliations:** a Institute of Fundamental Medicine and Biology, Kazan Federal University, Kazan, Russia; b Kazan Institute of Biochemistry and Biophysics, FRC Kazan Scientific Center of RAS, Kazan, Russia; c Riken Cluster for Science, Technology and Innovation Hub, Riken, Yokohama, Kanagawa, Japan; d Riken Center for Integrative Medical Sciences, Riken, Yokohama, Kanagawa, Japan

**Keywords:** Anhydrobiosis, Microbiome, Radioresistance, Metagenomics, Chironomids

## Abstract

The ability of larvae of a non-biting midge *Polypedilum vanderplanki* (Chironomidae) to withstand complete desiccation is a remarkable natural example of adaptation to extreme environment. In anhydrobiosis the larvae lose up to 99.2% of water and stay in a dry form until rainfall in natural environment or up to several decades in laboratory maintaining ability to restore activity soon after rehydration [1]. In the desiccated state, the larvae tolerate a variety of abiotic stresses, including high radiation exposure (7000Gry of ^60^Co gamma rays) [2]. Such a cross-resistance to desiccation and ionizing radiation is a characteristic of many anhydrobiotic organisms and believed to be based on similar molecular mechanisms.

Microorganisms associated with the anhydrobiotic midge can also sustain desiccation and thus be radiation-resistant because desiccation-resistant prokaryotes are shown to be cross-resistant to ionizing radiation [3]. Microorganisms inhabiting larvae of the anhydrobiotic midge can also sustain desiccation and probably can sustain high doses of ionizing radiation.

Therefore, it would be of interest to analyze the taxonomic and functional composition of microbiome of the anhydrobiotic midge. Sequencing data for the total DNA of anhydrobiotic organisms, which also contain reads derived from symbiotic microorganisms provide a promising opportunity to identify microorganisms with remarkable adaptation. It is known that at least some protective genes, such as late embryogenesis abundant (LEA) genes appeared in the genome of the midge by probable horizontal gene transfer from bacteria [1]. We performed shotgun sequencing of imago and larvae DNA samples using HiSeq 2000 and Genome Analyzer IIX System platforms. To assess microbiome diversity specific to anhydrobiotic midges, we analyzed Pool-seq data of the natural population of imago and Pool-seq data of final instar larvae. Data has been deposited in NCBI BioProject repository at NCBI under the accession number PRJNA659554 and consists of raw sequence data.

## Specifications Table

**Table:** 

Subject	Environmental Science
Specific subject area	Determination of the microbiome composition of Pool-seq data of the anhydrobiotic midge at different life cycles
Type of data	Figures
How data were acquired	Shotgun sequencing using HiSeq 2000 platform and Genome AnalyzerIIX System (Illumina, San Diego, CA, USA). Bioinformatic approach: taxonomic profiling of unmapped reads using kraken2 software [4]
Data format	Raw Analyzed Filtered
Parameters for data collection	Imago samples were collected from granite rocks outcrops in the natural habitat of the anhydrobiotic midge in Nigeria. Inbred line of midge larvae was cultivated by the anhydrobiosis research group in the National Institute of Agrobiological Sciences. Sequenced data was used to perform microbiome analysis of reads that were not mapped to the reference genome of the anhydrobiotic midge.
Description of data collection	DNA from the imago sample was extracted using NucleoSpin Tissue kit (Clontech Takara). DNA from the larvae sample was extracted with conventional cetrimonium bromide (CTAB) method and NucleoSpin tissue kit (Macherey-Nagel, Germany).
Data source location	Institutions: Kazan (Volga region) Federal University; Moscow State University; National Institute of Agrobiological Sciences City/Town/Region: Kazan, Republic of Tatarstan; Moscow; Tsukuba, Ibaraki Country: Russia; Japan
Data accessibility	Repository name: NCBI Bioproject Data identification number: PRJNA659554 Direct URL to data: https://www.ncbi.nlm.nih.gov/bioproject/?term=PRJNA659554

## Value of the Data

• The data presents microbiome diversity of the anhydrobiotic midge that can lose up almost all water in the larval stage and replace it with trehalose.
• The data allow researchers to evaluate microbiome composition and analyze differences of microbiome between two successive stages of insect development.
• The data can be used to detect microorganisms that probably can tolerate and adapt to harsh environments such as ionizing radiation and desiccation inside of extremophile species.

## 1 Data Description

**Fig. 1:**
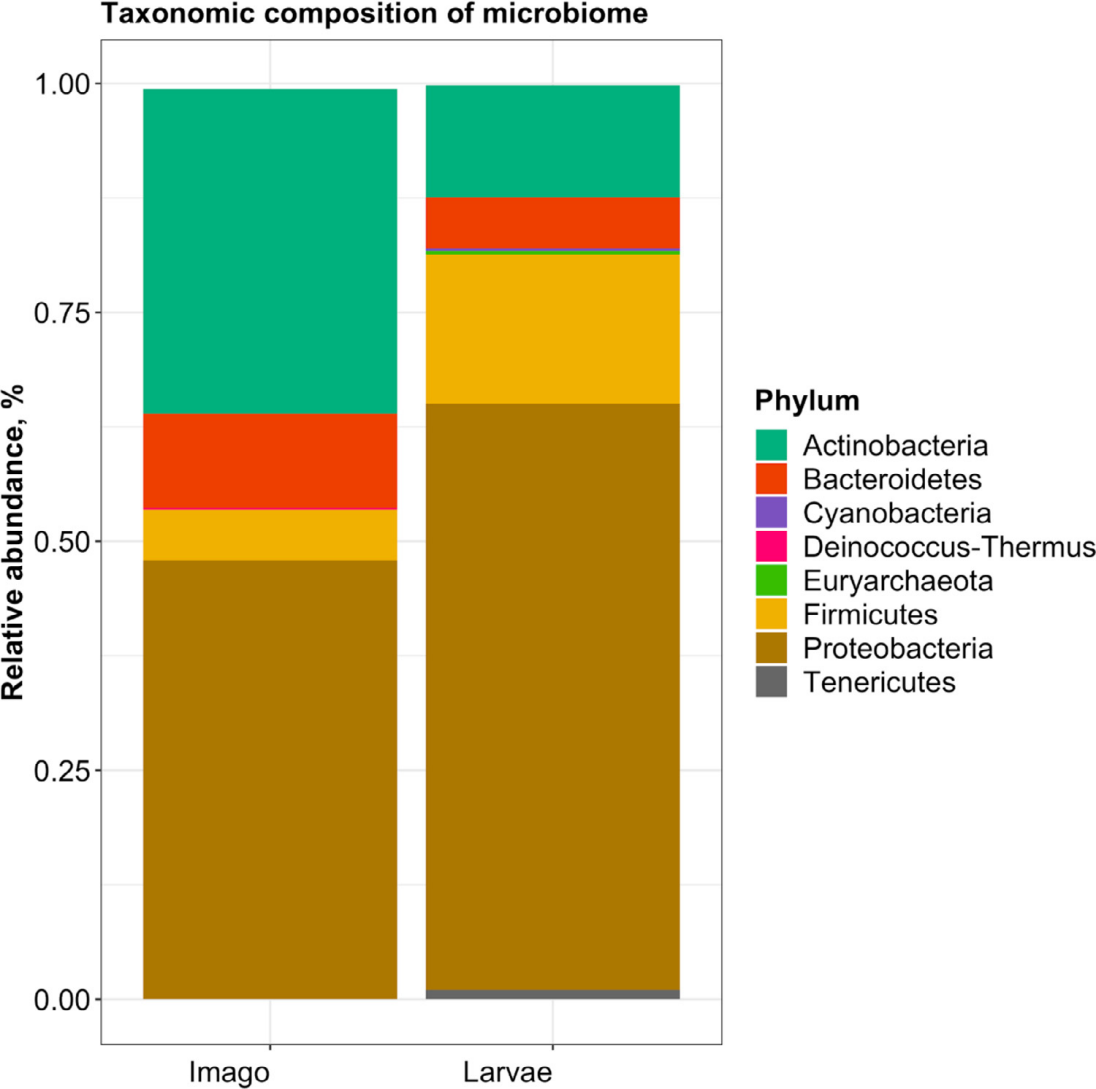
Comparison of the relative abundance of the most common phyla in the samples of adults and larvae. Using kraken2 software we find that imago and larvae share the same bacterial phyla, but they have a different relative abundance of them that was found by bracken software.

**Fig. 2:**
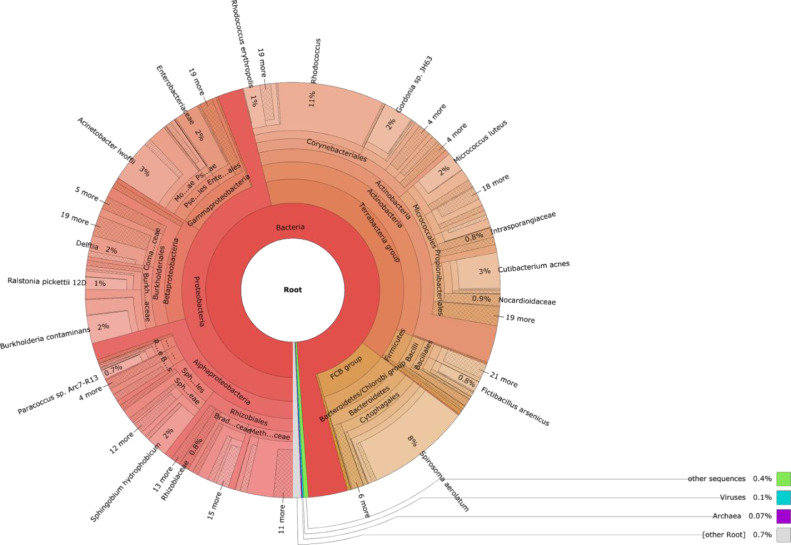
Metagenomic diversity of the imago stage of *P. vanderplanki*.

**Fig. 3:**
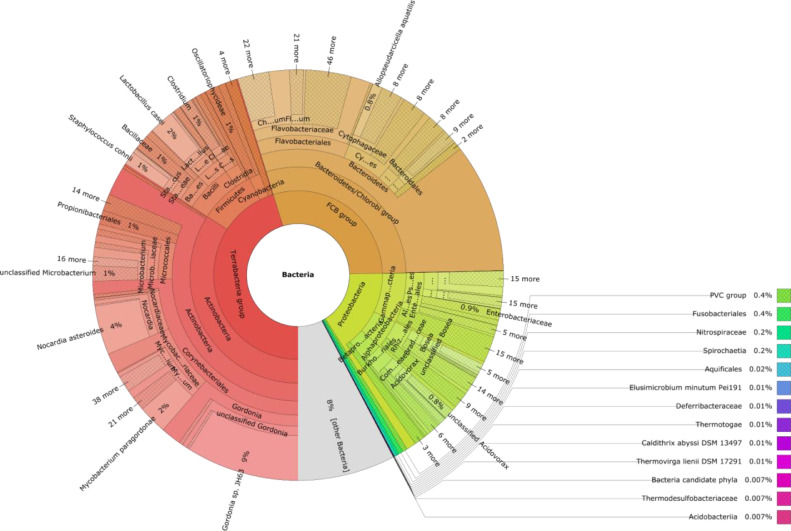
Metagenomic diversity of larvae stage of *P. vanderplanki*.

The dataset contains fastq files derived from Pool-seq data that refer only to microbiome of two successive stages of midge development. Contamination sequences were identified and removed from this dataset. There are two fastq files that contains 207,381 and 419,976 reads, respectively. The raw data files were deposited in NCBI BioProject under reference number: PRJNA659554. The composition of the microbiome of two samples (imago and larvae stages) were illustrated in Fig. 1–3. Taxonomic profiling of imago microbiome using the kraken2 database showed that the most abundant domain was Bacteria, followed by Viruses (0.1%) and Archaea (0.07%) (Fig. 2). Out of the 36 phyla present, the most abundant were Proteobacteria (47.9%) followed by Actinobacteria (35.4%) and Bacteroidetes (10.2%). Moreover, a total of 72 classes and 1112 genus were identified in imago microbiome.

Taxonomic profiling of larvae microbiome using kraken2 database showed that the most abundant domain was also Bacteria, followed by Archaea (0.07%) and Viruses (0.05%) (Fig. 3). Out of the 31 phyla present, the most abundant were Proteobacteria (64%) followed by Firmicutes (16.3%), Acidobacteria (12.3%) and Bacteroidetes (5.5%). Moreover, a total of 61 classes and 771 genus were identified in larvae microbiome.

Files used for taxonomic visualization can be found in the supplementary material and represent kraken2 software output files.

## 2 Experimental Design, Materials and Methods

### 2.1 Sample collection and DNA extraction

Pool of adult individuals (imago) from the natural habitat of *P. vanderplanki* were collected in 2015 in Nigeria, homogenized with polypropylene pestle in Eppendorf plastic tubes (1.5 mL). Genomic DNA was extracted from 12 adult individuals (imago) from the population using NucleoSpin Tissue kit (Clontech Takara), according to the manufacturer instruction.

Over 500 final instar larvae (inbred line) individuals were collected from NARO (Japan) in 2014 and used for genomic DNA extraction. After homogenisation with polypropylene pestle in Eppendorf plastic tubes (1.5 mL), highly pure genomic DNA was extracted using conventional cetrimonium bromide (CTAB) method with further purification by NucleoSpin tissue kit (Macherey-Nagel, Germany) (cat #740952). Genomic DNA concentration was estimated using a Qubit 3.0 fluorometer (Invitrogen) with Quantifluor dsDNA system (Promega).

### 2.2 Libraries preparation and sequencing

gDNA was fragmented using Covaris s220 (USA) DNA shearing protocol. Length of DNA fragments was estimated using Agilent Bioanalyzer 2100 (Agilent technologies). Libraries from each pool of gDNA were prepared using NEBNext Ultra II DNA Library Prep Kit for Illumina (cat #E7645L) following manufacturer's protocol. The concentration of libraries was measured by Qubit 3.0 fluorometer (Invitrogen), its quality was verified on the Bioanalyzer using DNA High Sensitivity chip, Agilent technologies (cat #5067-4626). Before sequencing, the number of molecules in each library was validated by real-time PCR using 2.5x Reaction mixture for PCR-RV in the presence of EVA Green (SINTOL, Russia) (cat #M-439) and primers for Illumina adapters (Evrogen, Russia).

Imago DNA libraries with final concentration 9 pM were clustered using cBot instrument (Illumina) with TruSeq PE Cluster Kit v3 (Illumina) (cat #PE-401-3001) and sequenced in pair-end mode (100 bp) with TruSeq SBS Kit v3 (cat# FC-401-3001) using Illumina HiSeq 2000 platform sequencing.

Larvae DNA libraries with final concentration 11 pM were sequenced on Illumina Genome Analyzer IIx System using TruSeq PE Cluster Kit v2 (cat #PE-300-2001) for cluster formation and TruSeq SBS Kit V5 (cat #FC-104-5001) with 100 bp paired end sequencing.

### 2.3 Taxonomic profiling

Raw data was processed through the standard NGS pipeline. Quality of raw paired-end reads were assessed using FastQC software [5]. Trimmomatic software (version 0.35) [6] was used to cut adapters and other illumina-specific sequences from raw data. Processed reads mapped to the current version of the reference genome of *P. vanderplanki* using BWA-MEM [7]. Then unmapped reads taken from alignment data and analyzed using kraken2 [4]. Kraken2 output files used to filter unmapped reads from unclassified and human contamination reads. Seqtk toolkit used for obtaining final dataset. To estimate relative abundance in each sample, kraken2 output of filtered data was used as input for bracken software [8]. Microbiome composition visualized using krona software [9].

## Author's contributions

Nurislam Shaikhutdinov: Methodology, Investigation, Visualization, Writing - Original Draft. Natalia Gogoleva: Methodology, Supervision. Oleg Gusev: Resources, Funding acquisition. Elena Shagimardanova: Conceptualization, Supervision, Funding acquisition.

## Ethics Statement

The work was not related to the use of human objects and did not include experiments with animals.

## Declaration of Competing Interest

The authors wish to declare that there are no conflicts of interest whatsoever, be it financial or personal. Hence, none of this was perceived to have influenced the outcome of the research reported herein in this data article.

## Appendix Supplementary materials

Image, application 1

Image, application 2

Image, application 3

Image, application 4

Image, application 5

Image, application 6
