# Supplementary material for: Microbiota composition data of imago and larval stage of the anhydrobiotic midge
Source: Data Brief. 2020 Nov 18;33:106527. doi: 10.1016/j.dib.2020.106527 (PMC7689402; doi:10.1016/j.dib.2020.106527)
Supplement: Supplementary file 4 [file mmc4.zip › mmc4/file_for_krona_visualization_of_imago.fig2.html]

Javascript must be enabled to view this page.

members
magnitude
magnitudeUnassigned
count
unassigned
taxon
rank

Anguantuta\_BWA\_MAPPER\_TRIMMED\_READS\_TO\_Pv11\_5.0\_sorted\_unmapped.filtered

419976
2757
node0.members.0.js

node1.members.0.js
no rank
28384
1498

12756
2
414928
node2.members.0.js
superkingdom

node3.members.0.js
phylum
1224
193664
8425

node4.members.0.js
class
1236
41417
1300

order
181
1706369

1706371
141
family

2
447467
genus

447471
2
species

strain
node9.members.0.js
2
1117647

2425
1
genus

2426
1
species

node12.members.0.js
strain
377629
1

genus
2
2036021

node14.members.0.js
species
1737490
2

genus
4
316625

species
86304
4

203122
4
node17.members.0.js
strain

4
genus
node18.members.0.js
132
10

18
122
2624793
no rank
node19.members.0.js

43
454662
species
node20.members.0.js

34
2303332
species
node21.members.0.js

5
1987723
species
node22.members.0.js

node23.members.0.js
species
1945512
22

node24.members.0.js
species
155077
6

family
16
1706373

genus
48073
16

species
node27.members.0.js
5
260552

48074
1
node28.members.0.js
species

no rank
2619833
9

1516059
2
node30.members.0.js
species

species
node31.members.0.js
3
359370

4
2591606
species
node32.members.0.js

species
node33.members.0.js
1
1769779

19
1706372
family

2
2678529
genus

no rank
2678530
2

node37.members.0.js
species
2708301
2

3
1706379
no rank

species
node39.members.0.js
3
2518989

14
1217416
genus

3
930805
species
node41.members.0.js

930806
11
node42.members.0.js
species

family
1706375
5

genus
2
630749

2631504
2
no rank

species
node46.members.0.js
2
1620392

1434050
1
genus

1470434
1
node48.members.0.js
species

genus
1084558
2

716816
2
node50.members.0.js
species

order
742030
4

family
742031
4

4
180541
genus

2649847
4
no rank

2183911
4
node55.members.0.js
species

9474
91347
order
node56.members.0.js
443

family
node57.members.0.js
8556
543
8080

genus
4
929812

node59.members.0.js
species
929813
4

570
50
node60.members.0.js
genus
26

node61.members.0.js
species
1463165
2

species
node62.members.0.js
6
573

548
5
node63.members.0.js
species

3
1134687
species
node64.members.0.js

8
571
species
node65.members.0.js

genus
1330546
7

61647
4
node67.members.0.js
species

node68.members.0.js
species
1334193
3

genus
2172100
1

1
2172103
species
node70.members.0.js

genus
1335483
5

species
node72.members.0.js
5
563

genus
5
2055880

species
node74.members.0.js
5
566

4
genus
node75.members.0.js
15
590

node76.members.0.js
species
28901
11
8

59201
3
subspecies

no rank
1
1243577

strain
node79.members.0.js
1
1243578

149539
2
node80.members.0.js
no rank

genus
17
413496

species
3
413497

subspecies
413498
3

1159554
3
node84.members.0.js
strain

6
413501
species

6
1159613
strain
node86.members.0.js

species
2
535744

2
1074000
strain
node88.members.0.js

node89.members.0.js
species
413503
4

1163710
2
species

2
1073999
strain
node91.members.0.js

40
node92.members.0.js
genus
547
109

1
885040
species
node93.members.0.js

2478464
6
node94.members.0.js
species

species group
node95.members.0.js
59
354276
37

14
550
15
node96.members.0.js
species

1
69219
subspecies

1
1104326
strain
node98.members.0.js

1
158836
species

301105
1
node100.members.0.js
subspecies

1
69218
species
node101.members.0.js

node102.members.0.js
species
61645
1

node103.members.0.js
species
299767
4

no rank
3
2608935

species
node105.members.0.js
2
1914861

1
1166130
species
node106.members.0.js

genus
8
1330545

2642424
2
no rank

species
node109.members.0.js
2
2153385

61646
2
node110.members.0.js
species

4
1907578
species
node111.members.0.js

9
genus
node112.members.0.js
104
561

node113.members.0.js
species
564
5

1499973
4
node114.members.0.js
species

208962
2
node115.members.0.js
species

no rank
1
2608889

1
2044467
species
node117.members.0.js

80
species
node118.members.0.js
83
562

83334
1
node119.members.0.js
serotype

2
866768
strain
node120.members.0.js

1330547
21
node121.members.0.js
genus
5

no rank
2632876
1

species
node123.members.0.js
1
2492396

208223
13
node124.members.0.js
species

2
1158459
species

node126.members.0.js
strain
1235834
2

no rank
8
36866

2052938
1
node128.members.0.js
species

node129.members.0.js
species
891974
2

species
node130.members.0.js
5
693444

genus
579
3

species
node132.members.0.js
3
61648

82976
3
genus

3
2634062
no rank

node135.members.0.js
species
2479367
3

genus
1903434
16

species
node137.members.0.js
16
565

genus
node138.members.0.js
36
83654
24

1
no rank
node139.members.0.js
6
2627398

node140.members.0.js
species
2282310
2

node141.members.0.js
species
2681307
1

2
2282309
species
node142.members.0.js

6
83655
species
node143.members.0.js

3
160674
10
node144.members.0.js
genus

54291
2
node145.members.0.js
species

5
577
species
node146.members.0.js

38
544
genus
node147.members.0.js
7

species
2
67825

637910
2
node149.members.0.js
strain

species
35703
2

strain
node151.members.0.js
2
1261127

2644389
3
no rank

3
2653932
species
node153.members.0.js

10
24
1344959
species group
node154.members.0.js

species
node155.members.0.js
5
67827

node156.members.0.js
species
546
9

genus
1
158483

158822
1
node158.members.0.js
species

15
620
genus
node159.members.0.js
2

species
node160.members.0.js
4
621

species
node161.members.0.js
8
622

species
node162.members.0.js
1
624

1903412
11
family

8
635
genus
node164.members.0.js
1

67780
1
node165.members.0.js
species

93378
4
node166.members.0.js
species

2
636
species
node167.members.0.js

568
3
node168.members.0.js
genus

1
1903409
214
node169.members.0.js
family

genus
82986
1

53336
1
node171.members.0.js
species

genus
32199
6

9
6
species

node174.members.0.js
forma specialis
1241869
4

forma specialis
node175.members.0.js
1
1265350

forma specialis
node176.members.0.js
1
557993

551
32
node177.members.0.js
genus
10

2622719
2
no rank

species
node179.members.0.js
2
215689

node180.members.0.js
species
1922217
3

1
338565
species

node182.members.0.js
strain
465817
1

species
node183.members.0.js
8
55211

node184.members.0.js
species
1619313
8

20
genus
node185.members.0.js
165
53335

no rank
2630326
53

node187.members.0.js
species
2479547
6

592316
5
node188.members.0.js
species

node189.members.0.js
species
2575375
10

node190.members.0.js
species
1484158
32

species group
51
1654067

549
51
node192.members.0.js
species

node193.members.0.js
species
59814
20

node194.members.0.js
species
1891675
6

node195.members.0.js
species
553
6

species
node196.members.0.js
9
470934

genus
node197.members.0.js
9
2100764
1

1458355
1
node198.members.0.js
species

species
node199.members.0.js
1
665913

node200.members.0.js
species
665914
6

1903414
22
family

583
5
genus

3
585
species
node203.members.0.js

species
node204.members.0.js
2
584

29487
2
genus

291112
1
node206.members.0.js
species

1
2218628
species

141679
1
node208.members.0.js
subspecies

637
1
genus

node210.members.0.js
species
638
1

genus
7
581

582
7
node212.members.0.js
species

node213.members.0.js
genus
586
7
1

node214.members.0.js
species
126385
4

species
node215.members.0.js
2
333965

no rank
451511
10

702
10
genus

703
10
node218.members.0.js
species

family
2
1903416

2
82984
genus

82985
2
node221.members.0.js
species

1903410
62
node222.members.0.js
family
3

8
genus
node223.members.0.js
25
204037

1
204038
species
node224.members.0.js

node225.members.0.js
species
1778540
1

species
node226.members.0.js
10
204042

no rank
node227.members.0.js
3
2622466

1
556
species

strain
node229.members.0.js
1
1223569

node230.members.0.js
species
568768
1

1
genus
node231.members.0.js
3
84565

species
63612
2

node233.members.0.js
strain
343509
2

7
19
122277
genus
node234.members.0.js

species
55208
2

strain
node236.members.0.js
2
1175631

2042057
5
node237.members.0.js
species

3
554
species

subspecies
node239.members.0.js
3
555

node240.members.0.js
species
180957
2

4
1082702
genus

node242.members.0.js
species
1082704
4

7
71655
8
node243.members.0.js
genus

node244.members.0.js
species
1109412
1

1903411
154
node245.members.0.js
family
6

34037
24
node246.members.0.js
genus
17

no rank
2635087
1

node248.members.0.js
species
1805933
1

3
species
node249.members.0.js
6
34038

node250.members.0.js
strain
745277
3

5
27
629
genus
node251.members.0.js

node252.members.0.js
species
630
13

no rank
4
2653513

node254.members.0.js
species
1179669
4

29486
1
node255.members.0.js
species

node256.members.0.js
species
29485
1

2607663
1
node257.members.0.js
species

species
29483
1

strain
node259.members.0.js
1
1453495

263819
1
node260.members.0.js
species

1
1964366
genus

2636213
1
no rank

node263.members.0.js
species
2126321
1

genus
1
1565532

species
node265.members.0.js
1
1646377

genus
2
1745211

species
1639108
2

1441930
2
node268.members.0.js
strain

49
node269.members.0.js
genus
613
91

28151
10
node270.members.0.js
species

3
species
node271.members.0.js
4
82996

node272.members.0.js
strain
1154756
1

node273.members.0.js
species
47917
7

species
node274.members.0.js
10
615

614
2
node275.members.0.js
species

node276.members.0.js
species
138074
4

618
5
node277.members.0.js
species

genus
1927833
2

node279.members.0.js
species
1878942
2

14
204
135622
order
node280.members.0.js

family
1
267891

1
58050
genus

node283.members.0.js
species
80854
1

family
1
267889

genus
1
28228

1
196834
no rank

species
node287.members.0.js
1
1816218

72275
62
family

genus
node289.members.0.js
27
2742
1

1
1874317
species
node290.members.0.js

species
node291.members.0.js
1
1420916

1420917
4
node292.members.0.js
species

2743
1
species

1
351348
strain
node294.members.0.js

8
83889
19
node295.members.0.js
no rank

2547598
9
node296.members.0.js
species

2488665
1
node297.members.0.js
species

1415568
1
node298.members.0.js
species

node299.members.0.js
genus
226
17
4

species
node300.members.0.js
5
314275

no rank
2614992
6

6
2267264
species
node302.members.0.js

species
node303.members.0.js
1
589873

node304.members.0.js
species
28108
1

2
2650549
genus

2
2259620
species
node306.members.0.js

5
1172191
genus

no rank
2638362
5

5
2172099
species
node309.members.0.js

genus
7
288793

2
2303538
species
node311.members.0.js

914153
1
node312.members.0.js
species

no rank
2645119
4

node314.members.0.js
species
2572577
4

1621534
2
genus

326544
2
species

1129794
2
node317.members.0.js
strain

261825
1
genus

680279
1
node319.members.0.js
species

genus
89404
1

2618684
1
no rank

species
node322.members.0.js
1
2489595

family
30
267888

8
genus
node324.members.0.js
30
53246

species
node325.members.0.js
9
43657

194690
2
no rank

2583375
2
node327.members.0.js
species

species
166935
5

strain
node329.members.0.js
5
1315283

species
node330.members.0.js
6
43662

2
267892
family

44011
2
genus

species
2
44012

2
550540
strain
node334.members.0.js

family
5
267893

no rank
5
946227

5
1298881
species
node337.members.0.js

80
267890
family

15
node339.members.0.js
genus
22
80

4
1965282
species
node340.members.0.js

225848
1
node341.members.0.js
species

4
2018305
species
node342.members.0.js

species
404011
1

strain
node344.members.0.js
1
225849

node345.members.0.js
species
24
1

species
271097
5

425104
5
node347.members.0.js
strain

70864
4
species

strain
node349.members.0.js
4
398579

species
node350.members.0.js
3
150120

no rank
196818
11

1930557
7
node352.members.0.js
species

species
node353.members.0.js
4
2698686

4
260364
species
node354.members.0.js

60478
1
species

1
326297
strain
node356.members.0.js

species
56812
18

318167
18
node358.members.0.js
strain

species
60961
6

6
392500
strain
node360.members.0.js

2
60217
species

node362.members.0.js
strain
637905
2

family
9
267894

9
67572
genus
node364.members.0.js
1

357794
8
species

node366.members.0.js
strain
357804
8

1692040
20
order

node368.members.0.js
family
1692041
20
1

genus
4
1692042

1675686
4
node370.members.0.js
species

12
986106
genus

2640868
12
no rank

node373.members.0.js
species
1281578
12

3
1744881
genus

node375.members.0.js
species
1620215
3

16
1934945
order

1934946
16
family

genus
1934947
16

1810504
16
node379.members.0.js
species

24
118969
order

16
444
family

8
genus
node382.members.0.js
16
445

4
28087
species
node383.members.0.js

456
1
node384.members.0.js
species

1
45056
species
node385.members.0.js

28082
1
node386.members.0.js
species

1
45067
species
node387.members.0.js

family
8
118968

genus
8
254245

8
254246
species
node390.members.0.js

74
135623
order

family
641
74

1
51366
genus

1
1908198
species
node394.members.0.js

58
662
genus
node395.members.0.js
5

1
190893
species
node396.members.0.js

675
1
node397.members.0.js
species

node398.members.0.js
species
673372
2

689
1
node399.members.0.js
species

28172
1
node400.members.0.js
species

node401.members.0.js
species
29497
3

2
672
species
node402.members.0.js

species
node403.members.0.js
2
2572923

species group
13
717610

1
680
species
node405.members.0.js

4
species
node406.members.0.js
6
670

node407.members.0.js
strain
1429044
2

node408.members.0.js
species
696485
6

6
55601
species
node409.members.0.js

species
4
246167

4
1191300
strain
node411.members.0.js

2
45658
species
node412.members.0.js

29494
2
node413.members.0.js
species

no rank
2614977
6

node415.members.0.js
species
2587865
1

species
node416.members.0.js
5
2662262

node417.members.0.js
species
1763883
1

6
676
species
node418.members.0.js

1
511678
genus

1
80852
species
node420.members.0.js

genus
13
657

5
38293
species
node422.members.0.js

species
3
1295392

3
658445
strain
node424.members.0.js

species
5
74109

node426.members.0.js
strain
298386
5

2042066
1
genus

node428.members.0.js
species
1755811
1

order
13
135615

family
13
868

2717
13
genus

node432.members.0.js
species
2718
13

no rank
118884
15

1608298
10
genus

1076588
10
node435.members.0.js
species

genus
3
745410

2291597
3
node437.members.0.js
species

32036
2
clade

species
2
113267

forma specialis
node440.members.0.js
2
1303921

order
135619
346

family
1920240
1

1
261963
genus

1
261964
species

node445.members.0.js
strain
523791
1

135620
17
family

1
267849
genus

no rank
1
2614692

2614693
1
node449.members.0.js
species

genus
4
1537406

1249553
4
node451.members.0.js
species

48075
1
genus

node453.members.0.js
species
1821621
1

genus
28253
11

no rank
196814
3

species
node456.members.0.js
1
2071621

400668
2
node457.members.0.js
species

species
936476
8

node459.members.0.js
strain
491952
8

439484
3
no rank

node461.members.0.js
species
2683272
3

family
9
255527

230494
5
genus

species
node464.members.0.js
5
1336806

genus
4
231683

node466.members.0.js
species
2161747
4

family
224379
14

14
158481
genus
node468.members.0.js
3

7
158327
species

7
349521
strain
node470.members.0.js

4
2624107
no rank

1628392
4
node472.members.0.js
species

family
449732
3

418700
3
genus

species
node475.members.0.js
3
418701

family
20
224372

1
genus
node477.members.0.js
15
59753

no rank
4
2638842

4
2014542
species
node479.members.0.js

species
285091
7

node481.members.0.js
strain
930169
7

species
3
1306787

3
391936
strain
node483.members.0.js

5
2025617
genus

1917421
5
node485.members.0.js
species

node486.members.0.js
family
28256
279
3

genus
12
404432

12
1771309
species
node488.members.0.js

114403
2
no rank

genus
33073
1

33074
1
node491.members.0.js
species

genus
114185
1

1
114186
species
node493.members.0.js

4
1897649
genus

node495.members.0.js
species
1883414
4

node496.members.0.js
genus
2745
226
16

species
node497.members.0.js
4
1897729

1
29570
species
node498.members.0.js

475662
11
node499.members.0.js
species

1081866
3
node500.members.0.js
species

4
node501.members.0.js
no rank
2609666
183

2497861
6
node502.members.0.js
species

species
node503.members.0.js
9
1883416

1761789
1
node504.members.0.js
species

2562282
10
node505.members.0.js
species

2
2576841
species
node506.members.0.js

species
node507.members.0.js
123
2306583

species
node508.members.0.js
10
2587849

1504981
8
node509.members.0.js
species

species
node510.members.0.js
2
1118153

2
2014541
species
node511.members.0.js

2587844
6
node512.members.0.js
species

species
2746
4

4
768066
strain
node514.members.0.js

507626
2
node515.members.0.js
species

node516.members.0.js
species
664683
2

2
13
504090
genus
node517.members.0.js

no rank
2648671
5

node519.members.0.js
species
657387
5

157779
4
node520.members.0.js
species

species
node521.members.0.js
2
698828

genus
6
42054

158080
6
species

290398
6
node524.members.0.js
strain

204286
3
node525.members.0.js
genus
2

2609414
1
no rank

2686360
1
node527.members.0.js
species

376488
10
genus

node529.members.0.js
species
376489
10

order
1
1240482

family
1
1240483

genus
1
1193503

1196095
1
node533.members.0.js
species

order
143
135625

143
712
family
node535.members.0.js
6

105
724
genus
node536.members.0.js
21

62
729
species
node537.members.0.js
51

strain
node538.members.0.js
11
862965

no rank
5
2609962

712310
5
node540.members.0.js
species

species
node541.members.0.js
9
726

node542.members.0.js
species
727
6
5

strain
node543.members.0.js
1
1295140

1
249188
species
node544.members.0.js

node545.members.0.js
species
735
1

2
476528
genus

node547.members.0.js
species
47735
2

416916
8
genus

node549.members.0.js
species
714
4

node550.members.0.js
species
732
4

genus
22
745

747
22
species

21
22
44283
subspecies
node553.members.0.js

1
1304873
strain
node554.members.0.js

order
32
1775403

568386
24
family

21
413435
genus

2637139
21
no rank

node559.members.0.js
species
2303331
21

1861863
3
genus

no rank
3
2621506

2698684
3
node562.members.0.js
species

family
8
2689614

8
469322
genus

8
465721
species
node565.members.0.js

4
order
node566.members.0.js
200
135613

255526
6
family

genus
6
109262

species
3
927

555778
3
node570.members.0.js
strain

no rank
3
2636392

node572.members.0.js
species
1860122
3

451214
1
no rank

1
1273155
genus

node575.members.0.js
species
585455
1

family
6
1676141

genus
6
1676142

species
node578.members.0.js
6
1579979

2
node579.members.0.js
family
1046
112

7
85073
genus

species
1050
7

node582.members.0.js
strain
316276
7

1227
6
node583.members.0.js
genus
2

species
node584.members.0.js
1
1814290

species
3
1229

323261
3
node586.members.0.js
strain

85076
17
genus

species
37487
17

765910
17
node589.members.0.js
strain

genus
85072
4

1049
4
species

572477
4
node592.members.0.js
strain

genus
51
67575

115860
51
no rank

2545632
1
node595.members.0.js
species

2498451
50
node596.members.0.js
species

15
53392
genus

15
1166950
species
node598.members.0.js

156885
5
genus

80679
5
species

node601.members.0.js
strain
765912
5

13724
5
genus

species
73141
5

5
765911
strain
node604.members.0.js

family
node605.members.0.js
60
72276
1

3
node606.members.0.js
genus
106633
23

no rank
2
2621013

species
node608.members.0.js
2
396595

186931
8
species

8
1255043
strain
node610.members.0.js

1
108010
species

node612.members.0.js
strain
713585
1

species
1033854
4

strain
node614.members.0.js
4
396588

species
node615.members.0.js
5
106634

7
genus
node616.members.0.js
16
1051

2684909
5
no rank

5
1442136
species
node618.members.0.js

4
421628
species
node619.members.0.js

1
187271
no rank

1
947515
species
node621.members.0.js

133193
3
genus

3
351052
species

3
187272
strain
node624.members.0.js

genus
85108
9

species
9
1053

strain
node627.members.0.js
9
349124

genus
1765964
4

node629.members.0.js
species
160660
2

1765967
2
node630.members.0.js
species

3
1335745
genus

species
node632.members.0.js
1
1855875

1335757
2
node633.members.0.js
species

2
2035710
family

genus
2
2035712

species
node636.members.0.js
2
47960

family
1096778
7

genus
7
2034504

species
node639.members.0.js
7
1972068

family
449719
2

genus
1
1860077

1
1811807
species
node642.members.0.js

genus
1
437504

species
1
437505

1
1192854
strain
node645.members.0.js

62
135618
order

52
403
family

genus
node648.members.0.js
15
416
2

1
1538553
species
node649.members.0.js

12
2608980
no rank

107637
1
node651.members.0.js
species

11
1727196
species
node652.members.0.js

3
node653.members.0.js
genus
39773
15

species
6
271065

1091494
6
node655.members.0.js
strain

no rank
2
2631527

node657.members.0.js
species
2049332
2

species
39775
4

4
686340
strain
node659.members.0.js

genus
6
762296

node661.members.0.js
species
1704499
6

genus
4
73778

node663.members.0.js
species
1432792
4

1
1808977
genus

1808979
1
node665.members.0.js
species

11
413
genus

414
11
species

strain
node668.members.0.js
11
243233

1486721
10
family

2749459
10
no rank

species
node671.members.0.js
10
417

37
72274
26393
node672.members.0.js
order

54
node673.members.0.js
family
135621
3403

subfamily
351
32

genus
node675.members.0.js
32
352
2

species
node676.members.0.js
12
354

node677.members.0.js
species
69964
13

5
353
species
node678.members.0.js
1

strain
node679.members.0.js
4
1328314

no rank
190017
1

node681.members.0.js
species
2079806
1

genus
1849530
1

1
1697053
species
node683.members.0.js

genus
1649479
7

species
node685.members.0.js
7
1510150

1380
node686.members.0.js
genus
286
3308

3
46677
species
node687.members.0.js

species
node688.members.0.js
3
364197

5
1499686
species
node689.members.0.js

species
node690.members.0.js
4
122355

603
136846
species group
node691.members.0.js
12

node692.members.0.js
species
47886
9

10
271420
species
node693.members.0.js

species
3
74829

node695.members.0.js
strain
1123016
3

species subgroup
578833
569

503
node697.members.0.js
species
316
569

4
379731
strain
node698.members.0.js

node699.members.0.js
strain
644801
5

strain
node700.members.0.js
46
1196835

11
1123519
strain
node701.members.0.js

487184
4
node702.members.0.js
species

node703.members.0.js
species
1434072
6

species
node704.members.0.js
1
157783

46
136849
species group
node705.members.0.js
24

251695
8
species subgroup

317
8
node707.members.0.js
species
2

no rank
4
264449

103796
3
node709.members.0.js
no rank

264451
1
node710.members.0.js
no rank

2
1332075
strain
node711.members.0.js

8
36746
species

strain
node713.members.0.js
8
1441629

1
33069
species
node714.members.0.js

3
251701
species

no rank
3
59511

3
629265
strain
node717.members.0.js

species subgroup
251698
2

2
47877
species

no rank
322
2

2
573066
strain
node721.members.0.js

species
node722.members.0.js
5
472181

196821
334
no rank

2604832
5
node724.members.0.js
species

node725.members.0.js
species
2320867
13

node726.members.0.js
species
1294143
7

species
node727.members.0.js
4
2083053

2
2054919
species
node728.members.0.js

species
node729.members.0.js
101
2054914

species
node730.members.0.js
8
1573719

6
1898684
species
node731.members.0.js

1
2505979
species
node732.members.0.js

species
node733.members.0.js
7
1573711

node734.members.0.js
species
2653853
26

species
node735.members.0.js
3
2201356

node736.members.0.js
species
2587845
6

node737.members.0.js
species
2597770
3

node738.members.0.js
species
1881017
2

2609418
1
node739.members.0.js
species

1886807
1
node740.members.0.js
species

1573704
1
node741.members.0.js
species

1
1755504
species
node742.members.0.js

253237
19
node743.members.0.js
species

species
node744.members.0.js
2
1283291

3
1855380
species
node745.members.0.js

7
2518644
species
node746.members.0.js

node747.members.0.js
species
1856685
2

2
2498848
species
node748.members.0.js

node749.members.0.js
species
2025658
8

node750.members.0.js
species
118613
2

2073078
1
node751.members.0.js
species

4
1028989
species
node752.members.0.js

6
1931241
species
node753.members.0.js

species
node754.members.0.js
1
1611770

node755.members.0.js
species
1207075
2

node756.members.0.js
species
658630
5

1
658629
species
node757.members.0.js

2126069
5
node758.members.0.js
species

76885
14
node759.members.0.js
species

2706126
5
node760.members.0.js
species

node761.members.0.js
species
1855331
1

6
143813
species
node762.members.0.js

1930532
7
node763.members.0.js
species

node764.members.0.js
species
1702250
1

3
2604941
species
node765.members.0.js

2
2213057
species
node766.members.0.js

1
1415630
species
node767.members.0.js

node768.members.0.js
species
2083055
1

1
2018067
species
node769.members.0.js

node770.members.0.js
species
2169583
3

species
node771.members.0.js
1
1573712

1573718
6
node772.members.0.js
species

5
1736226
species
node773.members.0.js

1573720
2
node774.members.0.js
species

2219057
2
node775.members.0.js
species

node776.members.0.js
species
2479392
5

3
1148509
species
node777.members.0.js

136843
157
node778.members.0.js
species group
15

47878
2
node779.members.0.js
species

node780.members.0.js
species
76758
13

node781.members.0.js
species
47883
16
14

strain
node782.members.0.js
2
96901

76760
6
node783.members.0.js
species

node784.members.0.js
species
651740
2

node785.members.0.js
species
29442
10

2
75588
species
node786.members.0.js

species
node787.members.0.js
1
200450

129817
6
node788.members.0.js
species

species
node789.members.0.js
7
46679

node790.members.0.js
species
200451
1

node791.members.0.js
species
75612
9

50
species
node792.members.0.js
62
294

node793.members.0.js
strain
205922
4

node794.members.0.js
strain
1221522
2

216595
1
node795.members.0.js
strain

1038922
5
node796.members.0.js
strain

4
5
380021
species
node797.members.0.js

isolate
node798.members.0.js
1
1420599

9
237609
species
node799.members.0.js

species
node800.members.0.js
10
104087

702115
3
node801.members.0.js
species

244566
4
node802.members.0.js
species

198618
6
node803.members.0.js
species

1
321846
species
node804.members.0.js

species
node805.members.0.js
4
553151

species
node806.members.0.js
9
65741

species group
54
136842

2
47884
species
node808.members.0.js

296
8
node809.members.0.js
species

species
node810.members.0.js
21
86185

22
587753
23
node811.members.0.js
species

1
86192
subspecies
node812.members.0.js

node813.members.0.js
species
198620
25

5
312306
species
node814.members.0.js
2

384676
3
node815.members.0.js
strain

node816.members.0.js
species
1788301
22

1
1615674
species
node817.members.0.js

species
node818.members.0.js
2
219572

species group
node819.members.0.js
228
136845
13

47885
27
node820.members.0.js
species

1
2217867
species
node821.members.0.js

5
70775
species
node822.members.0.js

species
6
47880

strain
node824.members.0.js
6
743720

species
node825.members.0.js
1
78327

node826.members.0.js
species
76759
17

303
158
node827.members.0.js
species
86

390235
69
node828.members.0.js
strain

strain
node829.members.0.js
1
1211579

1384061
2
node830.members.0.js
strain

node831.members.0.js
species
797277
1

25
101564
species

strain
node833.members.0.js
25
741155

359110
2
node834.members.0.js
species

16
node835.members.0.js
species group
136841
228

53412
16
species

node837.members.0.js
strain
1245471
16

node838.members.0.js
species
287
62
60

strain
node839.members.0.js
1
1457392

node840.members.0.js
strain
910265
1

species subgroup
8
627141

node842.members.0.js
species
46680
8

species subgroup
1232139
57

10
1149133
species
node844.members.0.js

species
node845.members.0.js
47
301

34
node846.members.0.js
species
300
41

1
1001585
strain
node847.members.0.js

6
399739
strain
node848.members.0.js

53408
14
node849.members.0.js
species

species
node850.members.0.js
14
43263

1274359
24
node851.members.0.js
species

node852.members.0.js
species
1302376
2

7
12
321662
species
node853.members.0.js

node854.members.0.js
strain
1395516
5

node855.members.0.js
species
191390
1

1392877
1
node856.members.0.js
species

species group
3
136844

3
43306
species
node858.members.0.js

3
95300
species
node859.members.0.js

1628086
2
node860.members.0.js
species

437900
4
node861.members.0.js
species

395598
1
node862.members.0.js
species

node863.members.0.js
species
1245526
9

21
237610
species
node864.members.0.js

species
node865.members.0.js
1
1421430

157782
13
node866.members.0.js
species

216142
11
node867.members.0.js
species

node868.members.0.js
species
930166
3

4
515393
species
node869.members.0.js

22953
468
family
node870.members.0.js
190

genus
537
475

480
3
node872.members.0.js
species

node873.members.0.js
species
386891
1

529
34062
species
node874.members.0.js

species
node875.members.0.js
4
34061

genus
node876.members.0.js
22095
469
5855

13957
species
node877.members.0.js
13959
28090

2
1046625
strain
node878.members.0.js

species
202950
4

4
62977
strain
node880.members.0.js

196816
334
node881.members.0.js
no rank
29

node882.members.0.js
species
1407071
7

5
2004644
species
node883.members.0.js

node884.members.0.js
species
2079596
14

node885.members.0.js
species
1636603
140

26
2004646
species
node886.members.0.js

species
node887.members.0.js
30
1646498

1608473
14
node888.members.0.js
species

1879049
8
node889.members.0.js
species

27
1808001
species
node890.members.0.js

29
2662362
species
node891.members.0.js

5
1758189
species
node892.members.0.js

node893.members.0.js
species
70348
10

32
species
node894.members.0.js
34
40216

strain
node895.members.0.js
2
981334

species
node896.members.0.js
1030
108981

7
202954
species
node897.members.0.js

species
node898.members.0.js
2
1324350

40215
68
node899.members.0.js
species

node900.members.0.js
species
756892
100

52133
4
species

4
1197884
strain
node902.members.0.js

909768
119
node903.members.0.js
species group
8

species
node904.members.0.js
9
106654

10
48296
species
node905.members.0.js

6
471
species
node906.members.0.js

species
node907.members.0.js
6
1785128

80
470
species
node908.members.0.js
75

node909.members.0.js
strain
1400867
1

strain
node910.members.0.js
4
1401639

node911.members.0.js
species
134534
2

1879050
14
node912.members.0.js
species

28
202956
species
node913.members.0.js

76
108980
species
node914.members.0.js

node915.members.0.js
species
1871111
5

53
106649
species
node916.members.0.js

6
2004650
species
node917.members.0.js

node918.members.0.js
species
487316
3

299
40214
310
node919.members.0.js
species

node920.members.0.js
strain
1242245
11

106648
3
node921.members.0.js
species

node922.members.0.js
species
1789224
9

species
node923.members.0.js
38
29430

2136182
22
node924.members.0.js
species

497
131
node925.members.0.js
genus
36

334543
9
species

259536
9
node927.members.0.js
strain

85
196806
no rank
node928.members.0.js
26

species
node929.members.0.js
17
1699621

species
node930.members.0.js
2
2203895

18
1720344
species
node931.members.0.js

1699624
2
node932.members.0.js
species

1699622
1
node933.members.0.js
species

node934.members.0.js
species
1699623
3

node935.members.0.js
species
1028416
7

8
571800
species
node936.members.0.js

1
349106
species
node937.members.0.js

node938.members.0.js
species
330922
1

135614
2685
node939.members.0.js
order
118

family
node940.members.0.js
124
1775411
9

genus
70411
13

species
81475
13

strain
node943.members.0.js
13
767434

22
242605
genus

species
242606
6

6
1440763
strain
node946.members.0.js

2589080
16
node947.members.0.js
species

genus
2233801
3

node949.members.0.js
species
2021234
3

5
323413
genus

5
323415
species

strain
node952.members.0.js
5
1300342

genus
8
2707020

node954.members.0.js
species
2010829
8

33
75309
genus

species
node956.members.0.js
9
582702

24
666685
species
node957.members.0.js

genus
231454
31

231455
10
species

node960.members.0.js
strain
1217721
10

node961.members.0.js
species
445710
21

513
family
node962.members.0.js
2443
32033

genus
2709666
2

2
2511995
species
node964.members.0.js

1
83618
65
node965.members.0.js
genus

species
node966.members.0.js
52
314722
27

743721
25
node967.members.0.js
strain

12
415229
species

1045855
12
node969.members.0.js
strain

334
890
40323
genus
node970.members.0.js

2
node971.members.0.js
no rank
196198
82

node972.members.0.js
species
1904944
44

1
1827305
species
node973.members.0.js

species
node974.members.0.js
7
2282124

node975.members.0.js
species
2005046
7

species
node976.members.0.js
1
2211160

2691571
18
node977.members.0.js
species

species
node978.members.0.js
1
2303750

species
node979.members.0.js
1
2546450

138
216778
species
node980.members.0.js

128780
26
node981.members.0.js
species

47
995085
310
node982.members.0.js
species group

235
238
40324
species
node983.members.0.js

2
868597
strain
node984.members.0.js

1
1190567
strain
node985.members.0.js

2072414
13
node986.members.0.js
species

2072413
7
node987.members.0.js
species

species
node988.members.0.js
1
2072407

2
2072412
species
node989.members.0.js

node990.members.0.js
species
2072406
2

genus
141948
58

58
2633315
no rank

species
node993.members.0.js
58
2202149

node994.members.0.js
genus
338
277
104

6
node995.members.0.js
species
56460
7

925775
1
node996.members.0.js
strain

7
node997.members.0.js
species
56448
14

node998.members.0.js
no rank
195709
6

69929
1
node999.members.0.js
no rank

25
343
species
node1000.members.0.js
8

152263
9
node1001.members.0.js
no rank

134875
2
no rank

2
1261556
strain
node1003.members.0.js

node1004.members.0.js
no rank
487909
6

species
56454
5

5
487904
no rank

863365
5
node1007.members.0.js
strain

species
node1008.members.0.js
3
29447

4
1985254
species

92828
4
no rank

1437877
4
node1011.members.0.js
strain

18
56458
species
node1012.members.0.js

species
node1013.members.0.js
5
48664

56453
8
node1014.members.0.js
species

species
node1015.members.0.js
7
56455

species
3
56450

strain
node1017.members.0.js
3
1219375

19
347
species
node1018.members.0.js
18

1
64187
no rank
node1019.members.0.js

56459
1
node1020.members.0.js
species

species
node1021.members.0.js
37
339
25

340
6
node1022.members.0.js
no rank

node1023.members.0.js
no rank
359385
6

643453
17
species group

2
node1025.members.0.js
species
346
17

node1026.members.0.js
no rank
611301
6

6
473426
no rank
node1027.members.0.js

node1028.members.0.js
no rank
473421
3

node1029.members.0.js
genus
68
487
80

453783
31
node1030.members.0.js
species

1324796
22
node1031.members.0.js
species

node1032.members.0.js
species
1605891
32

2698682
172
node1033.members.0.js
species

node1034.members.0.js
species
84531
15

node1035.members.0.js
species
262324
10

node1036.members.0.js
species
435897
12

node1037.members.0.js
species
2591633
17

69
46
node1038.members.0.js
species

no rank
50
2635362

node1040.members.0.js
species
2290922
50

83614
138
genus

node1042.members.0.js
no rank
2629088
103
15

46
1176533
species
node1043.members.0.js

node1044.members.0.js
species
1896164
13

29
2508168
species
node1045.members.0.js

2006110
19
node1046.members.0.js
species

2565782
16
node1047.members.0.js
species

2
2370
genus

node1049.members.0.js
species
1444770
1

2371
1
species

subspecies
671135
1

node1052.members.0.js
strain
155920
1

genus
490567
11

370777
11
node1054.members.0.js
species

14
72273
order

135616
7
family

genus
28884
2

2
39765
species
node1058.members.0.js
1

317025
1
node1059.members.0.js
strain

genus
933
1

1
147268
species

node1062.members.0.js
strain
717773
1

genus
2
2039723

species
node1064.members.0.js
2
2267253

40222
2
genus

754476
2
node1066.members.0.js
species

6
34064
family

1
genus
node1068.members.0.js
6
262

5
573570
species
node1069.members.0.js

1
135617
family

1
1021
genus

species
node1072.members.0.js
1
288004

135624
202
order

node1074.members.0.js
family
84642
202
6

genus
5
129577

5
2636315
no rank

node1077.members.0.js
species
511062
5

96
node1078.members.0.js
genus
642
183

node1079.members.0.js
species
948519
4

no rank
node1080.members.0.js
18
257493
5

node1081.members.0.js
species
2033033
3

9
2033032
species
node1082.members.0.js

1
1636606
species
node1083.members.0.js

1
73010
species
node1084.members.0.js

196024
2
node1085.members.0.js
species

node1086.members.0.js
species
654
2

4
645
species
node1087.members.0.js
2

2
96473
subspecies

1324960
2
node1089.members.0.js
strain

27
species
node1090.members.0.js
30
651

1208104
3
node1091.members.0.js
strain

20
26
644
species
node1092.members.0.js

subspecies
196023
5

380703
5
node1094.members.0.js
strain

node1095.members.0.js
strain
1354302
1

genus
8
347533

347534
8
node1097.members.0.js
species

14
33811
no rank

species
node1099.members.0.js
2
947516

12
1248727
species
node1100.members.0.js

1807140
19
class

order
225057
19

family
225058
19

10
19
119977
genus
node1104.members.0.js

2
3
160808
species
node1105.members.0.js

743299
1
node1106.members.0.js
strain

species
node1107.members.0.js
6
33059
4

2
637389
strain
node1108.members.0.js

node1109.members.0.js
class
28211
87631
5511

2543
order
node1110.members.0.js
44287
356

41294
14145
node1111.members.0.js
family
1650

3518
374
10587
node1112.members.0.js
genus

species
node1113.members.0.js
96
1274631

node1114.members.0.js
species
255045
74

119
190148
species
node1115.members.0.js

1355477
67
node1116.members.0.js
species

1404367
53
node1117.members.0.js
species

node1118.members.0.js
species
244734
597

931866
8
node1119.members.0.js
species

node1120.members.0.js
species
1325107
80

1325095
229
node1121.members.0.js
species

1325115
76
node1122.members.0.js
species

node1123.members.0.js
no rank
2631580
5026
943

16
319017
species
node1124.members.0.js

1404864
35
node1125.members.0.js
species

node1126.members.0.js
species
858422
86

species
node1127.members.0.js
31
2493093

species
node1128.members.0.js
65
167468

node1129.members.0.js
species
114615
53

node1130.members.0.js
species
376
62

species
node1131.members.0.js
615
1197460

1223566
65
node1132.members.0.js
species

node1133.members.0.js
species
288000
233

node1134.members.0.js
species
2599819
4

2725
2057741
species
node1135.members.0.js

species
node1136.members.0.js
23
335659

species
node1137.members.0.js
65
115808

species
node1138.members.0.js
5
2599805

91
375
103
node1139.members.0.js
species

476282
12
node1140.members.0.js
strain

210
1437360
species
node1141.members.0.js

49
44255
species

1245469
49
node1143.members.0.js
strain

node1144.members.0.js
species
1404768
39

722472
92
node1145.members.0.js
species

node1146.members.0.js
species
1325090
151

104
911
genus
node1147.members.0.js
8

species
28
913

strain
node1149.members.0.js
28
323098

species
68
912

strain
node1151.members.0.js
68
323097

genus
29
1649510

species
node1153.members.0.js
29
1333996

1073
426
genus

426
1076
species
node1155.members.0.js
209

strain
node1156.members.0.js
33
258594

316056
61
node1157.members.0.js
strain

strain
node1158.members.0.js
8
395960

316055
35
node1159.members.0.js
strain

316058
71
node1160.members.0.js
strain

strain
node1161.members.0.js
9
652103

51
40136
genus

40137
51
node1163.members.0.js
species

genus
1033
66

no rank
2642050
66

node1166.members.0.js
species
1882747
66

85413
908
node1167.members.0.js
genus
85

species
node1168.members.0.js
57
1526658

125
no rank
node1169.members.0.js
766
2653178

99
1867715
species
node1170.members.0.js

node1171.members.0.js
species
2015316
174

species
node1172.members.0.js
241
2599640

species
node1173.members.0.js
74
1842539

species
node1174.members.0.js
53
1792307

1395974
109
genus

107
no rank
node1176.members.0.js
109
2631404

species
node1177.members.0.js
2
2592814

no rank
215
81426

species
node1179.members.0.js
215
709797

family
255475
73

414371
22
genus

2615206
22
no rank

22
1349819
species
node1183.members.0.js

1
51
293088
genus
node1184.members.0.js

10
1486262
species
node1185.members.0.js

2629616
24
no rank

24
686597
species
node1187.members.0.js

species
16
293089

16
1122214
strain
node1189.members.0.js

4
335928
190
node1190.members.0.js
family

genus
21
152053

species
21
921

node1193.members.0.js
strain
639283
21

5
genus
node1194.members.0.js
45
556257

no rank
2638514
20

20
2562284
species
node1196.members.0.js

331696
20
node1197.members.0.js
species

genus
204476
21

node1199.members.0.js
species
376174
21

99
33
genus

no rank
2626613
18

18
1850374
species
node1202.members.0.js

15
1745854
species
node1203.members.0.js

279
44
genus

44
280
species

44
78245
strain
node1206.members.0.js

genus
6
22

22
7
species

438753
22
node1209.members.0.js
strain

45401
489
node1210.members.0.js
family
20

genus
node1211.members.0.js
45
59282
1

species
node1212.members.0.js
22
1079

22
2233851
species
node1213.members.0.js

genus
55
29407

55
2619116
no rank

node1216.members.0.js
species
674703
55

genus
1082930
44

531813
44
species

node1219.members.0.js
strain
1082931
44

1827478
33
genus

species
node1221.members.0.js
33
1447062

9
1068
genus

species
9
1069

648757
9
node1224.members.0.js
strain

genus
34
81

2
node1226.members.0.js
species
53399
7

strain
node1227.members.0.js
3
582899

node1228.members.0.js
strain
670307
2

no rank
2619925
16

717785
16
node1230.members.0.js
species

11
1427356
species

node1232.members.0.js
strain
1029756
11

genus
5
119044

node1234.members.0.js
species
1608628
5

244
46913
genus
node1235.members.0.js
17

no rank
node1236.members.0.js
147
196773
6

2499144
28
node1237.members.0.js
species

node1238.members.0.js
species
1736675
44

node1239.members.0.js
species
2083786
69

node1240.members.0.js
species
400770
80

2269
119045
21179
node1241.members.0.js
family

407
15054
node1242.members.0.js
genus
8798

species
146
114616

node1244.members.0.js
strain
460265
146

1351
270351
species
node1245.members.0.js

68
39956
species

68
908290
strain
node1247.members.0.js

species
node1248.members.0.js
390
418223

739
2202827
species
node1249.members.0.js

31998
36
species

strain
node1251.members.0.js
36
426355

52
334852
species

52
693986
strain
node1253.members.0.js

2202825
322
node1254.members.0.js
species

54
1680
2615210
no rank
node1255.members.0.js

70
739141
species
node1256.members.0.js

43
925818
species
node1257.members.0.js

2603276
228
node1258.members.0.js
species

887
2202826
species
node1259.members.0.js

node1260.members.0.js
species
426117
127

species
node1261.members.0.js
140
2067957

38
1479019
species
node1262.members.0.js

93
2202828
species
node1263.members.0.js

2051553
1369
node1264.members.0.js
species

269660
103
node1265.members.0.js
species

186650
68
node1266.members.0.js
genus
3

1882682
38
node1267.members.0.js
species

no rank
2617746
27

13
2651334
species
node1269.members.0.js

species
node1270.members.0.js
14
2082949

node1271.members.0.js
genus
2282523
3788
1696

773
408
1834
node1272.members.0.js
species

strain
node1273.members.0.js
115
440085

419610
157
node1274.members.0.js
strain

789
272630
strain
node1275.members.0.js

173
node1276.members.0.js
species
223967
220

strain
node1277.members.0.js
47
441620

38
29429
species
node1278.members.0.js

14
119043
family

genus
5
1541445

node1281.members.0.js
species
1458461
5

genus
8
256616

species
8
256618

strain
node1284.members.0.js
8
402881

no rank
1712260
1

1
2026785
species
node1286.members.0.js

6
node1287.members.0.js
family
118882
122

18
node1288.members.0.js
genus
234
24

4
2632610
6
node1289.members.0.js
no rank

2
1885919
species
node1290.members.0.js

92
528
genus
node1291.members.0.js
19

58
529
59
node1292.members.0.js
species

node1293.members.0.js
strain
439375
1

271865
10
node1294.members.0.js
species

node1295.members.0.js
species
571256
4

family
node1296.members.0.js
1966
69277
65

genus
28100
17

17
1867719
species
node1298.members.0.js

2
31988
66
node1299.members.0.js
genus

42
2644704
no rank

node1301.members.0.js
species
374606
42

node1302.members.0.js
species
83263
22

20
245876
genus

2641084
20
no rank

species
node1305.members.0.js
6
1756988

2599600
14
node1306.members.0.js
species

genus
6
449972

no rank
6
2643807

6
266779
species
node1309.members.0.js

genus
4
2712688

2712698
4
node1311.members.0.js
species

317
node1312.members.0.js
genus
68287
1759

702
325217
no rank
node1313.members.0.js
105

node1314.members.0.js
species
2493671
9

node1315.members.0.js
species
278153
2

2493668
16
node1316.members.0.js
species

species
node1317.members.0.js
12
2493672

6
1854057
species
node1318.members.0.js

2584466
38
node1319.members.0.js
species

4
2493681
species
node1320.members.0.js

11
2108445
species
node1321.members.0.js

17
2493676
species
node1322.members.0.js

2493675
26
node1323.members.0.js
species

4
2493674
species
node1324.members.0.js

node1325.members.0.js
species
2493669
13

6
2493670
species
node1326.members.0.js

node1327.members.0.js
species
2493673
15

node1328.members.0.js
species
2082387
384

2493677
7
node1329.members.0.js
species

2493678
27
node1330.members.0.js
species

species
node1331.members.0.js
8
1670800

species
71433
20

20
1082933
strain
node1333.members.0.js

2
2066070
11
node1334.members.0.js
species

4
935547
strain
node1335.members.0.js

5
266835
strain
node1336.members.0.js

species
18
593909

536019
18
node1338.members.0.js
strain

species
5
28104

763057
5
node1340.members.0.js
strain

6
node1341.members.0.js
species
39645
11

5
682633
strain
node1342.members.0.js

44
381
species
node1343.members.0.js
15

935546
29
node1344.members.0.js
strain

19
536018
species

19
754035
strain
node1346.members.0.js

node1347.members.0.js
species
1295366
604

16
274591
genus

species
244596
14

node1350.members.0.js
strain
411684
14

no rank
2
2614931

species
node1352.members.0.js
2
1620421

genus
1649463
2

2
2647570
no rank

2305987
2
node1355.members.0.js
species

genus
11
1915401

node1357.members.0.js
species
1852022
11

no rank
80
119042

1572860
25
genus

25
1482074
species
node1360.members.0.js

9
1484898
genus

1384459
4
node1362.members.0.js
species

no rank
2617503
5

5
2170729
species
node1364.members.0.js

genus
14
169055

no rank
14
2623691

node1367.members.0.js
species
2712222
14

32
1734920
genus

species
node1369.members.0.js
32
1235591

286
82115
3256
node1370.members.0.js
family

2661800
14
genus

node1372.members.0.js
species
2341112
14

586
no rank
node1373.members.0.js
2496
227290

6
141
1525371
genus
node1374.members.0.js

48
2629175
no rank

node1376.members.0.js
species
2060726
19

node1377.members.0.js
species
1825976
29

87
399
species
node1378.members.0.js
33

323656
30
no rank

node1380.members.0.js
strain
1028801
30

no rank
323655
24

1028800
24
node1382.members.0.js
strain

node1383.members.0.js
genus
379
655
180

9
node1384.members.0.js
species
29449
11

538025
2
node1385.members.0.js
strain

node1386.members.0.js
species
240521
11

node1387.members.0.js
no rank
2613769
182
29

node1388.members.0.js
species
1301032
5

6
1914541
species
node1389.members.0.js

node1390.members.0.js
species
2028343
14

2048897
17
node1391.members.0.js
species

node1392.members.0.js
species
2364272
4

2603277
37
node1393.members.0.js
species

136098
1
node1394.members.0.js
species

node1395.members.0.js
species
2590777
7

node1396.members.0.js
species
1869170
14

species
node1397.members.0.js
5
2020312

1981173
10
node1398.members.0.js
species

species
node1399.members.0.js
8
424182

17
1571470
species
node1400.members.0.js

node1401.members.0.js
species
1435607
8

12
396
14
node1402.members.0.js
species

2
526949
strain
node1403.members.0.js

node1404.members.0.js
species
348824
15

species
node1405.members.0.js
9
451876

56730
6
node1406.members.0.js
species

node1407.members.0.js
species
2267833
4

node1408.members.0.js
species
1335061
34

398
19
species

node1410.members.0.js
strain
698761
19

species
11
448181

11
1336235
strain
node1412.members.0.js

5
648995
species
node1413.members.0.js

species
node1414.members.0.js
2
1538158

node1415.members.0.js
species
379684
26

species
node1416.members.0.js
17
1120045

13
1312183
species
node1417.members.0.js

49
node1418.members.0.js
species
384
96

no rank
386
27

strain
node1420.members.0.js
7
1033991

strain
node1421.members.0.js
11
395491

395492
2
node1422.members.0.js
strain

strain
node1423.members.0.js
7
754523

20
387
no rank
node1424.members.0.js
14

strain
node1425.members.0.js
6
216596

genus
node1426.members.0.js
1114
357
155

species
node1427.members.0.js
9
160699

826
1183400
species group
node1428.members.0.js
32

species
node1429.members.0.js
651
358
631

node1430.members.0.js
strain
1300224
4

311403
15
node1431.members.0.js
strain

1
1300225
strain
node1432.members.0.js

node1433.members.0.js
species
1176649
143
87

node1434.members.0.js
strain
176299
56

species
node1435.members.0.js
26
359

46
node1436.members.0.js
no rank
2632611
68

1
861208
species
node1437.members.0.js

species
node1438.members.0.js
10
2664893

2580515
4
node1439.members.0.js
species

species
node1440.members.0.js
7
1842536

30
373
species

30
311402
strain
node1442.members.0.js

genus
143
323620

143
2643062
no rank

node1445.members.0.js
species
879274
143

12
node1446.members.0.js
no rank
227292
316

node1447.members.0.js
genus
28105
229
26

2613772
114
no rank

node1449.members.0.js
species
430451
5

109
1842534
species
node1450.members.0.js

1
node1451.members.0.js
species
194963
3

2
1408224
strain
node1452.members.0.js

110321
9
species

366394
9
node1454.members.0.js
strain

41
382
42
node1455.members.0.js
species

node1456.members.0.js
strain
693982
1

species group
663276
35

22
species
node1458.members.0.js
35
380

strain
node1459.members.0.js
4
1128334

1128331
1
node1460.members.0.js
strain

4
1185652
strain
node1461.members.0.js

strain
node1462.members.0.js
1
1117943

node1463.members.0.js
strain
394
3

75
106591
genus
node1464.members.0.js
2

716925
9
species

9
716928
strain
node1466.members.0.js

species
node1467.members.0.js
49
106592
25

node1468.members.0.js
strain
1416753
24

15
1752398
species
node1469.members.0.js

1
34019
genus

species
node1471.members.0.js
1
1273132

83
45404
family

1156568
10
genus

10
569860
species
node1474.members.0.js

genus
532
10

533
10
species

31994
10
subspecies

10
395963
strain
node1478.members.0.js

45405
24
no rank

23
2572036
species
node1480.members.0.js

node1481.members.0.js
species
1978229
1

genus
120652
39

species
node1483.members.0.js
12
227605

species
199596
27

395965
27
node1485.members.0.js
strain

19
655351
family

15
1406135
genus

2624130
15
no rank

species
node1489.members.0.js
15
2304600

655352
4
genus

no rank
4
2631913

1798205
4
node1492.members.0.js
species

family
2723775
12

genus
2723776
12

2528642
12
node1495.members.0.js
species

772
1
family

773
1
genus

1
2645622
no rank
node1498.members.0.js

2036754
41
family

genus
node1500.members.0.js
41
28209
13

node1501.members.0.js
species
444444
17

11
2638111
no rank

node1503.members.0.js
species
1702325
11

74
31993
family
node1504.members.0.js
1

52
133
genus
node1505.members.0.js
1

2625913
4
no rank

node1507.members.0.js
species
187303
4

8
391905
species
node1508.members.0.js

14
134
species
node1509.members.0.js

node1510.members.0.js
species
655015
18

node1511.members.0.js
species
173366
7

425
20
genus

6
2624500
no rank

node1514.members.0.js
species
2699395
6

species
14
426

strain
node1516.members.0.js
14
595536

196080
1
genus

no rank
2614952
1

species
node1519.members.0.js
1
2560057

204458
8053
order

node1521.members.0.js
family
76892
8053
421

no rank
node1522.members.0.js
50
81440
4

28
2686094
species
node1523.members.0.js

node1524.members.0.js
species
1759059
18

76890
22
genus

18
22
78587
species
node1526.members.0.js

node1527.members.0.js
strain
573065
4

node1528.members.0.js
genus
20
76
9

2640670
8
no rank

node1530.members.0.js
species
2201350
8

284016
59
species

strain
node1532.members.0.js
59
450851

1011
node1533.members.0.js
genus
75
3021

785
2648921
no rank
node1534.members.0.js
39

366602
109
node1535.members.0.js
species

514
69665
species
node1536.members.0.js

node1537.members.0.js
species
2708539
123

88688
335
node1538.members.0.js
species

node1539.members.0.js
species
155892
465

1679497
191
node1540.members.0.js
species

75
69395
species
node1541.members.0.js

2010972
109
node1542.members.0.js
species

50
69666
species
node1543.members.0.js

node1544.members.0.js
genus
41275
4463
2003

node1545.members.0.js
species
588932
242

74329
197
node1546.members.0.js
species

species
node1547.members.0.js
54
1325724

node1548.members.0.js
species
41276
125

519
2622653
1637
node1549.members.0.js
no rank

species
node1550.members.0.js
120
2562582

1938605
125
node1551.members.0.js
species

2591463
216
node1552.members.0.js
species

species
node1553.members.0.js
190
1532555

2561924
96
node1554.members.0.js
species

species
node1555.members.0.js
163
2579977

node1556.members.0.js
species
2560058
43

node1557.members.0.js
species
1827469
165

species
100
74313

100
633149
strain
node1559.members.0.js

species
node1560.members.0.js
105
293

order
1191478
1

1191479
1
family

162171
1
genus

species
1
1124597

node1565.members.0.js
strain
156889
1

order
766
11

no rank
1
1699067

1
1528098
species
node1568.members.0.js

775
3
family

tribe
33988
3

780
2
genus

114277
1
species group

no rank
2750824
1

species
node1574.members.0.js
1
444612

species group
1
1129742

species
node1576.members.0.js
1
788

1
69474
genus

species
node1578.members.0.js
1
784

942
7
family

2
768
genus

770
2
node1581.members.0.js
species

genus
943
2

species group
2
106178

species
node1584.members.0.js
2
779

tribe
952
3

genus
node1586.members.0.js
3
953

1650
204457
23064
node1587.members.0.js
order

family
node1588.members.0.js
19421
41297
3230

72173
42
genus

2630544
42
no rank

42
1634516
species
node1591.members.0.js

genus
1649486
86

species
node1593.members.0.js
86
1850238

genus
68
335405

node1595.members.0.js
species
335406
14

2644549
54
no rank

1892855
54
node1597.members.0.js
species

2676233
46
genus

no rank
46
2676235

node1600.members.0.js
species
2711215
46

genus
1434046
48

7
48
2614947
no rank
node1602.members.0.js

15
2077182
species
node1603.members.0.js

15
2584094
species
node1604.members.0.js

11
1806885
species
node1605.members.0.js

41
genus
node1606.members.0.js
765
165696

species
169176
21

1219035
21
node1608.members.0.js
strain

node1609.members.0.js
species
158500
37

205844
62
species

node1611.members.0.js
strain
1088721
62

node1612.members.0.js
no rank
2644732
533
5

species
node1613.members.0.js
43
1609758

164608
57
node1614.members.0.js
species

species
node1615.members.0.js
27
702113

node1616.members.0.js
species
2675225
35

species
node1617.members.0.js
35
1016987

node1618.members.0.js
species
2571749
331

species
node1619.members.0.js
46
1176536

5
node1620.members.0.js
species
48935
25

20
279238
strain
node1621.members.0.js

21
2685927
genus

21
1921510
species
node1623.members.0.js

771
13687
4223
node1624.members.0.js
genus

species
node1625.members.0.js
108
1560345

species
node1626.members.0.js
78
13689

397260
94
species

strain
node1628.members.0.js
94
1123269

species
node1629.members.0.js
192
93064

1549858
95
node1630.members.0.js
species

no rank
node1631.members.0.js
1875
196159
245

91
2599297
species
node1632.members.0.js

127
1327635
species
node1633.members.0.js

2219696
100
node1634.members.0.js
species

node1635.members.0.js
species
1938607
133

node1636.members.0.js
species
1961362
49

356
2565555
species
node1637.members.0.js

42
745310
species
node1638.members.0.js

2681549
67
node1639.members.0.js
species

2698679
41
node1640.members.0.js
species

193
1523415
species
node1641.members.0.js

14
1381597
species
node1642.members.0.js

node1643.members.0.js
species
1813879
74

node1644.members.0.js
species
2594473
31

2711156
46
node1645.members.0.js
species

304378
131
node1646.members.0.js
species

node1647.members.0.js
species
1030157
35

species
node1648.members.0.js
63
1390395

15
2492837
species
node1649.members.0.js

22
2319844
species
node1650.members.0.js

node1651.members.0.js
species
941907
42

64
160791
species

392499
64
node1653.members.0.js
strain

46
363835
species
node1654.members.0.js

species
node1655.members.0.js
56
424800

node1656.members.0.js
species
152682
654
523

131
621456
strain
node1657.members.0.js

148
1609977
species
node1658.members.0.js

1724
10236
165695
genus
node1659.members.0.js

species
node1660.members.0.js
449
13690

2611147
785
node1661.members.0.js
no rank
62

species
node1662.members.0.js
45
2072936

34
2676077
species
node1663.members.0.js

species
node1664.members.0.js
16
407020

species
node1665.members.0.js
46
1855519

node1666.members.0.js
species
2082188
289

species
node1667.members.0.js
30
484429

species
node1668.members.0.js
53
2565554

node1669.members.0.js
species
1843368
114

12
2185111
species
node1670.members.0.js

627192
19
node1671.members.0.js
species

species
node1672.members.0.js
65
1315974

332056
8
species

452662
8
node1674.members.0.js
strain

44
1332080
species
node1675.members.0.js

species
node1676.members.0.js
57
135719

7001
1673076
species
node1677.members.0.js

species
46429
22

strain
node1679.members.0.js
22
690566

species
332055
2

node1681.members.0.js
strain
861109
2

species
node1682.members.0.js
45
76947

species
18
336203

node1684.members.0.js
strain
1208342
18

120107
81
node1685.members.0.js
species

genus
1
541

species
node1687.members.0.js
1
542

40
150203
63
node1688.members.0.js
genus

7
1550728
species
node1689.members.0.js

2626550
16
no rank

node1691.members.0.js
species
1842535
16

165697
592
node1692.members.0.js
genus
154

11
no rank
node1693.members.0.js
237
2614943

node1694.members.0.js
species
1913578
4

species
node1695.members.0.js
55
292913

species
node1696.members.0.js
31
1357916

node1697.members.0.js
species
1874061
53

species
node1698.members.0.js
20
1866325

node1699.members.0.js
species
2565556
24

species
node1700.members.0.js
39
1914525

33050
64
node1701.members.0.js
species

2054227
50
node1702.members.0.js
species

node1703.members.0.js
species
267128
11

49
117207
species

node1705.members.0.js
strain
317655
49

species
node1706.members.0.js
27
1515612

family
node1707.members.0.js
1993
335929
381

678
361177
genus
node1708.members.0.js
31

22
1267766
species
node1709.members.0.js

12
476157
species
node1710.members.0.js

no rank
node1711.members.0.js
396
2614945
6

165
2067415
species
node1712.members.0.js

species
node1713.members.0.js
36
2604537

33
2185142
species
node1714.members.0.js

species
node1715.members.0.js
106
2338327

2060312
50
node1716.members.0.js
species

species
node1717.members.0.js
42
543877

34
645517
species
node1718.members.0.js

species
node1719.members.0.js
30
361183

1982042
29
node1720.members.0.js
species

692370
82
node1721.members.0.js
species

12
336
1111
genus
node1722.members.0.js

18
285
2683265
no rank
node1723.members.0.js

1896196
33
node1724.members.0.js
species

176
2547601
species
node1725.members.0.js

species
node1726.members.0.js
20
2023229

node1727.members.0.js
species
2003315
38

species
node1728.members.0.js
39
1112

genus
128
1295327

species
node1730.members.0.js
62
1348774

450378
66
node1731.members.0.js
species

genus
1855416
43

species
node1733.members.0.js
43
1532023

427
1041
genus
node1734.members.0.js
30

266951
33
node1735.members.0.js
species

16
2182384
species
node1736.members.0.js

21
502682
species
node1737.members.0.js

species
node1738.members.0.js
139
192812

species
node1739.members.0.js
29
1648404

3
no rank
node1740.members.0.js
85
2633097

node1741.members.0.js
species
2011159
14

1798193
18
node1742.members.0.js
species

node1743.members.0.js
species
2502843
33

node1744.members.0.js
species
2587851
17

species
node1745.members.0.js
74
39960
21

node1746.members.0.js
strain
314225
53

1921002
4
order

1777752
1
family

1
1521255
genus

species
node1750.members.0.js
1
91604

family
3
44746

2601651
1
genus

species
node1753.members.0.js
1
2601668

no rank
204454
1

species
node1755.members.0.js
1
86106

2601574
1
node1756.members.0.js
genus

10
204455
5396
node1757.members.0.js
order

69657
32
family

6
74317
genus

species
6
74318

strain
node1761.members.0.js
6
394221

1433402
6
genus

1434191
6
node1763.members.0.js
species

85
20
genus

species
81032
6

6
228405
strain
node1766.members.0.js

no rank
14
2630699

4
1906738
species
node1768.members.0.js

1873716
10
node1769.members.0.js
species

node1770.members.0.js
family
31989
5354
489

40
60136
genus

4
1402135
species
node1772.members.0.js

196795
36
no rank

species
node1774.members.0.js
2
664426

node1775.members.0.js
species
2070369
3

species
node1776.members.0.js
9
1968541

1389004
6
node1777.members.0.js
species

species
node1778.members.0.js
2
1389011

species
node1779.members.0.js
6
2587855

8
1917485
species
node1780.members.0.js

genus
12
2211641

node1782.members.0.js
species
245188
12

2
191028
18
node1783.members.0.js
genus

6
2614906
no rank

node1785.members.0.js
species
2508306
4

2
2508307
species
node1786.members.0.js

133924
6
species

node1788.members.0.js
strain
999552
6

species
node1789.members.0.js
4
1396826

4
238783
genus

no rank
4
2644082

node1792.members.0.js
species
2500533
4

26
34008
genus
node1793.members.0.js
2

node1794.members.0.js
species
35806
4

20
2631432
no rank

node1796.members.0.js
species
308754
15

1564506
5
node1797.members.0.js
species

2
node1798.members.0.js
genus
74030
34

10
540747
species
node1799.members.0.js

215743
1
node1800.members.0.js
species

2614913
21
node1801.members.0.js
no rank
8

node1802.members.0.js
species
2587846
1

2587847
5
node1803.members.0.js
species

2587850
1
node1804.members.0.js
species

6
2494550
species
node1805.members.0.js

6
74032
genus

74033
6
node1807.members.0.js
species

2
258255
genus

2627060
2
no rank

species
node1810.members.0.js
2
911045

11
44
478070
genus
node1811.members.0.js

187304
2
node1812.members.0.js
species

5
388408
species

node1814.members.0.js
strain
244592
5

no rank
node1815.members.0.js
26
2648686
9

2021862
5
node1816.members.0.js
species

2590016
4
node1817.members.0.js
species

8
2587861
species
node1818.members.0.js

genus
387095
6

6
2639808
no rank

species
node1821.members.0.js
6
2587842

genus
1648497
2

2
2634418
no rank

species
node1824.members.0.js
2
2552942

1844498
7
genus

node1826.members.0.js
species
1505036
7

119541
10
genus

species
node1828.members.0.js
10
441209

5
299261
genus

5
299262
species
node1830.members.0.js

genus
58842
8

no rank
8
2624628

8
2009329
species
node1833.members.0.js

12
204456
genus

no rank
12
2636157

node1836.members.0.js
species
2169400
12

genus
16
436357

no rank
16
2633072

16
2017482
species
node1839.members.0.js

10
1855413
genus

node1841.members.0.js
species
1267768
10

12
360528
genus

node1843.members.0.js
species
1250539
12

genus
92944
17

3
92947
species
node1845.members.0.js

node1846.members.0.js
species
92945
14

188905
3
genus

2645469
3
no rank

3
290400
species
node1849.members.0.js

genus
93682
14

no rank
node1851.members.0.js
14
2639302
8

2587852
6
node1852.members.0.js
species

genus
node1853.members.0.js
4022
265
270

species
node1854.members.0.js
71
1945662

34003
29
species

1367847
29
node1856.members.0.js
strain

129
node1857.members.0.js
no rank
246570
3239

2560053
89
node1858.members.0.js
species

2966
2500532
species
node1859.members.0.js

node1860.members.0.js
species
2259340
23

node1861.members.0.js
species
1529068
32

31
225362
species
node1862.members.0.js

19
82367
species
node1863.members.0.js

node1864.members.0.js
species
147645
206

species
node1865.members.0.js
2
453842

135740
18
node1866.members.0.js
species

species
node1867.members.0.js
8
59779

5
1499308
species
node1868.members.0.js

node1869.members.0.js
species
266
26

2065379
42
node1870.members.0.js
species

species
node1871.members.0.js
29
1077935

34004
27
node1872.members.0.js
species

36
152161
genus
node1873.members.0.js
1

no rank
2629676
12

12
1881061
species
node1875.members.0.js

species
node1876.members.0.js
23
538381

285107
9
genus

node1878.members.0.js
species
1915078
9

genus
366614
14

195105
14
node1880.members.0.js
species

2433
2
genus

species
1
42443

strain
node1883.members.0.js
1
391595

node1884.members.0.js
species
2434
1

genus
13
367771

species
42444
13

13
988812
strain
node1887.members.0.js

genus
1097466
19

19
1335048
species
node1889.members.0.js

node1890.members.0.js
genus
1060
104
6

37
1063
species
node1891.members.0.js
35

557760
1
node1892.members.0.js
strain

strain
node1893.members.0.js
1
349101

species
21
1061

node1895.members.0.js
strain
272942
21

196779
29
no rank

1850250
12
node1897.members.0.js
species

2033869
17
node1898.members.0.js
species

11
1075
species
node1899.members.0.js

1579315
4
genus

species
node1901.members.0.js
4
1579316

8
947567
genus

no rank
8
2620315

node1904.members.0.js
species
2599296
8

302485
29
node1905.members.0.js
genus
3

60890
17
node1906.members.0.js
species

no rank
2
2621772

681157
2
node1908.members.0.js
species

1
221822
species
node1909.members.0.js

species
node1910.members.0.js
4
1580596

species
node1911.members.0.js
2
1844006

genus
27
875170

species
node1913.members.0.js
4
1411902

14
1208324
species
node1914.members.0.js

875171
1
node1915.members.0.js
species

node1916.members.0.js
species
1758178
5

1397108
3
node1917.members.0.js
species

genus
252301
6

6
2628660
no rank

2587163
6
node1920.members.0.js
species

genus
10
309512

10
215813
species

398580
10
node1923.members.0.js
strain

4
1609958
genus

no rank
2643318
4

4
1609966
species
node1926.members.0.js

159345
9
genus

159346
9
species

1294273
9
node1929.members.0.js
strain

53945
12
genus

1458307
3
node1931.members.0.js
species

no rank
1
196158

species
node1933.members.0.js
1
2602067

1
1217908
species

node1935.members.0.js
strain
391626
1

species
7
53946

strain
node1937.members.0.js
7
391616

263377
14
genus

1229727
14
node1939.members.0.js
species

1443919
28
genus

node1941.members.0.js
species
2494374
28

2
2738399
genus

2
2483033
species
node1943.members.0.js

genus
17
1955420

node1945.members.0.js
species
1826607
17

genus
node1946.members.0.js
22
97050
2

no rank
2625375
15

species
node1948.members.0.js
6
2293862

185588
3
node1949.members.0.js
species

species
node1950.members.0.js
6
292414

5
89184
species

246200
5
node1952.members.0.js
strain

58840
54
no rank

1
2171755
species
node1954.members.0.js

2605946
10
node1955.members.0.js
species

species
node1956.members.0.js
6
2683284

node1957.members.0.js
species
1904441
10

species
node1958.members.0.js
27
2033435

227873
35
genus

35
121719
species
node1960.members.0.js
29

strain
node1961.members.0.js
6
1402210

19
354203
genus
node1962.members.0.js
1

no rank
6
2645800

node1964.members.0.js
species
1792508
6

node1965.members.0.js
species
311180
12

4
2661844
genus

node1967.members.0.js
species
2552766
4

genus
2683599
28

28
2579971
species
node1969.members.0.js

genus
2
1759396

2
2622455
no rank

2
1920883
species
node1972.members.0.js

4
1649279
genus

species
node1974.members.0.js
4
379347
3

node1975.members.0.js
strain
1265309
1

order
2066490
7

2066491
7
family

genus
7
1602338

no rank
2644914
7

2582913
7
node1980.members.0.js
species

54526
5
order

family
1655514
5

198251
5
genus

5
2647897
no rank

5
1977865
species
node1985.members.0.js

82117
136
node1986.members.0.js
no rank
2

991903
17
genus

species
991904
17

17
991905
strain
node1989.members.0.js

3
114
1632780
genus
node1990.members.0.js

node1991.members.0.js
species
1868589
32

node1992.members.0.js
species
1940610
48

no rank
2644610
31

species
node1994.members.0.js
31
2570229

213485
3
genus

species
349221
3

node1997.members.0.js
strain
349215
3

order
node1998.members.0.js
1155
204441
11

41295
871
node1999.members.0.js
family
13

207
genus
node2000.members.0.js
569
191

node2001.members.0.js
species
192
47
41

6
1064539
strain
node2002.members.0.js

species
node2003.members.0.js
35
1226968

1
species
node2004.members.0.js
51
193

862719
27
node2005.members.0.js
strain

137722
23
node2006.members.0.js
strain

species
node2007.members.0.js
20
2202148

11
156
2630922
no rank
node2008.members.0.js

15
664962
species
node2009.members.0.js

709810
54
node2010.members.0.js
species

76
652764
species
node2011.members.0.js

682998
23
node2012.members.0.js
species

528244
30
node2013.members.0.js
species

57
1231242
genus

node2015.members.0.js
species
2594003
57

genus
1612157
13

1084
13
species

1150469
13
node2018.members.0.js
strain

genus
1263978
2

species
2
1263979

node2021.members.0.js
strain
1401328
2

2705399
24
genus

species
node2023.members.0.js
13
2602015

11
2602016
species
node2024.members.0.js

40
168934
genus
node2025.members.0.js
1

species
220697
18

strain
node2027.members.0.js
18
1123366

node2028.members.0.js
species
2048283
16

5
1891279
species
node2029.members.0.js

genus
1182780
8

no rank
2685734
8

8
1288970
species
node2032.members.0.js

1081
14
genus

node2034.members.0.js
species
1085
4

34018
10
species

strain
node2036.members.0.js
10
414684

genus
9
171436

species
171437
9

9
1110502
strain
node2039.members.0.js

genus
22
204447

2233999
22
node2041.members.0.js
species

7
node2042.members.0.js
genus
13134
42

55518
5
node2043.members.0.js
species

species
13
84159

13
342108
strain
node2045.members.0.js

17
2617991
no rank
node2046.members.0.js
1

node2047.members.0.js
species
1639348
7

1663591
9
node2048.members.0.js
species

genus
15
1543704

1612173
15
node2050.members.0.js
species

1543705
22
genus

species
22
28077

22
1441467
strain
node2053.members.0.js

2478349
21
genus

2220096
21
node2055.members.0.js
species

433
273
node2056.members.0.js
family
9

2603324
50
genus

1969806
50
node2058.members.0.js
species

19
genus
node2059.members.0.js
33
434

151157
1
subgenus

species
node2061.members.0.js
1
435

431306
1
node2062.members.0.js
species

446692
6
node2063.members.0.js
species

species
node2064.members.0.js
2
146474

no rank
2628570
1

2592655
1
node2066.members.0.js
species

438
3
node2067.members.0.js
species

genus
1223423
2

661191
2
node2069.members.0.js
species

4
1649499
genus

no rank
4
2637697

species
node2072.members.0.js
4
2558361

genus
153497
2

2
153496
species
node2074.members.0.js

genus
1602345
1

node2076.members.0.js
species
1510841
1

genus
6
441

species
4
442

strain
node2079.members.0.js
2
290633

1288313
2
node2080.members.0.js
strain

318683
2
node2081.members.0.js
species

genus
320496
12

species
node2083.members.0.js
12
320497

genus
91914
4

species
4
91915

1231624
4
node2086.members.0.js
strain

522
17
node2087.members.0.js
genus
14

species
524
3

node2089.members.0.js
strain
349163
3

no rank
1
41293

species
node2091.members.0.js
1
1909293

29
93
genus

29
94
species
node2093.members.0.js

125216
63
node2094.members.0.js
genus
29

2617492
3
no rank

node2096.members.0.js
species
2018065
3

species
node2097.members.0.js
5
207340

species
node2098.members.0.js
26
257708

4
genus
node2099.members.0.js
23
1434011

436
1
species

node2101.members.0.js
strain
714995
1

5
28448
6
node2102.members.0.js
species

1296990
1
node2103.members.0.js
strain

node2104.members.0.js
species
265959
2

species
node2105.members.0.js
1
65958

species
node2106.members.0.js
5
265960

1177712
4
species

4
634177
strain
node2108.members.0.js

genus
7
364409

species
node2110.members.0.js
7
364410

89583
10
genus

10
33996
species

10
272568
strain
node2113.members.0.js

order
255473
1

family
1
255474

208215
1
genus

species
1
208216

1
314260
strain
node2118.members.0.js

subphylum
700
68525

4
node2120.members.0.js
class
28221
604

413
29
order
node2121.members.0.js
3

5
228
80811
suborder
node2122.members.0.js

family
node2123.members.0.js
82
31
2

83461
24
genus

node2125.members.0.js
species
184914
19
11

1144275
8
node2126.members.0.js
strain

3
5
35
species
node2127.members.0.js

1189310
2
node2128.members.0.js
strain

6
56
32
genus
node2129.members.0.js

species
node2130.members.0.js
7
1297742

node2131.members.0.js
species
34
19
10

9
246197
strain
node2132.members.0.js

83455
24
species

strain
node2134.members.0.js
24
1278073

family
54
1524215

54
161492
genus
node2136.members.0.js
18

node2137.members.0.js
species
161493
19
2

14
290397
strain
node2138.members.0.js

455488
3
node2139.members.0.js
strain

no rank
2620896
17

species
node2141.members.0.js
7
447217

404589
10
node2142.members.0.js
species

12
76
39
family
node2143.members.0.js

44
16
genus

species
16
83453

16
1294270
strain
node2146.members.0.js

genus
6
42

node2148.members.0.js
species
43
6

30
47
genus

species
node2150.members.0.js
30
48

40
12
genus

species
41
12

378806
12
node2153.members.0.js
strain

family
1524213
11

11
1524214
genus

node2156.members.0.js
species
1391653
11

224462
21
suborder

family
224464
21

21
162027
genus

21
80816
species

502025
21
node2161.members.0.js
strain

5
node2162.members.0.js
suborder
80812
161

family
1524216
11

11
1524217
genus

11
1391654
species
node2165.members.0.js

no rank
215910
22

22
1649470
genus

22
888845
species
node2168.members.0.js

family
107
49

genus
39643
97

species
node2171.members.0.js
97
56
59

node2172.members.0.js
strain
448385
25

strain
node2173.members.0.js
13
1254432

genus
50
10

10
52
species
node2175.members.0.js

1055686
16
family

genus
1055688
16

16
927083
species
node2178.members.0.js

order
213462
11

family
213468
8

genus
43773
4

316277
4
species

strain
node2183.members.0.js
4
56780

2
2357
genus

species
2358
2

2
706587
strain
node2186.members.0.js

60892
2
genus

2
60893
species

880072
2
node2189.members.0.js
strain

3
213465
family

1
361106
genus

1
361111
species

1
980445
strain
node2193.members.0.js

2
29526
genus

species
119484
2

335543
2
node2196.members.0.js
strain

33
order
node2197.members.0.js
34
1779134

2099666
1
no rank

node2199.members.0.js
species
2600177
1

5
453227
order

5
453228
family

453229
5
genus

453230
5
species

strain
node2204.members.0.js
5
644282

69541
55
order

family
18
213421

genus
15
18

29542
5
node2208.members.0.js
species

species
29543
8

node2210.members.0.js
strain
338966
8

2
2620299
no rank

1842532
2
node2212.members.0.js
species

genus
3
890

2614637
1
no rank

node2215.members.0.js
species
1823759
1

species
node2216.members.0.js
2
1603606

213422
37
family

28231
37
node2218.members.0.js
genus
10

225194
2
species

2
404380
strain
node2220.members.0.js

species
313985
3

node2222.members.0.js
strain
398767
3

1
28232
species

strain
node2224.members.0.js
1
269799

species
node2225.members.0.js
3
345632

35554
3
node2226.members.0.js
species

no rank
2627627
13

species
node2228.members.0.js
6
2597769

4
443143
species
node2229.members.0.js

3
443144
species
node2230.members.0.js

1203471
2
species

strain
node2232.members.0.js
2
316067

213118
33
order

213121
8
family

893
1
genus

1
1986146
species
node2236.members.0.js

genus
427922
5

427923
5
species

strain
node2239.members.0.js
5
589865

1
109168
genus

species
84980
1

node2242.members.0.js
strain
177439
1

53318
1
genus

65555
1
species

1
1167006
strain
node2245.members.0.js

25
213119
family

2299
10
genus

node2248.members.0.js
species
947919
4

5
571177
species
node2249.members.0.js

species
node2250.members.0.js
1
83564

genus
12
896

1
181663
species

node2253.members.0.js
strain
96561
1

node2254.members.0.js
species
897
11

genus
2
218207

218208
2
node2256.members.0.js
species

2295
1
genus

species
1
2296

node2259.members.0.js
strain
177437
1

2
node2260.members.0.js
order
213115
49

43
194924
family

genus
15
2035811

9
2661612
no rank

node2264.members.0.js
species
2678688
9

species
182210
6

strain
node2266.members.0.js
6
643562

2
genus
node2267.members.0.js
23
872

901
1
node2268.members.0.js
species

58180
1
species

207559
1
node2270.members.0.js
strain

876
2
node2271.members.0.js
species

node2272.members.0.js
species
241368
2

species
node2273.members.0.js
3
296842

2593640
2
no rank

2583823
1
node2275.members.0.js
species

species
node2276.members.0.js
1
631220

1
184917
species

node2278.members.0.js
strain
573370
1

881
4
node2279.members.0.js
species
2

node2280.members.0.js
strain
883
2

345370
5
species

5
694327
subspecies
node2282.members.0.js

2582840
5
genus

species
873
5

subspecies
5
1511600

690850
5
node2286.members.0.js
strain

family
4
213116

genus
898
4

4
899
species

node2290.members.0.js
strain
525897
4

class
96
29547

94
213849
order

node2293.members.0.js
family
72293
8
2

202746
1
genus

node2295.members.0.js
no rank
2623549
1

genus
4
209

217
1
node2297.members.0.js
species

1
210
2
node2298.members.0.js
species

node2299.members.0.js
strain
1055530
1

species
node2300.members.0.js
1
104628

genus
1
286130

species
148813
1

709032
1
node2303.members.0.js
strain

438853
1
no rank

1
269260
genus

species
1
269261

strain
node2307.members.0.js
1
749222

85
72294
family

6
57665
genus

species
1
194424

node2311.members.0.js
strain
1193502
1

5
2618290
no rank

node2313.members.0.js
species
1581011
5

7
60
194
genus
node2314.members.0.js

1
1244531
species
node2315.members.0.js

no rank
2
2593542

1500960
1
node2317.members.0.js
species

1
1660076
species
node2318.members.0.js

204
3
node2319.members.0.js
species

195
2
node2320.members.0.js
species

3
827
species

3
1032069
strain
node2322.members.0.js

9
200
species
node2323.members.0.js

3
201
species
node2324.members.0.js

5
node2325.members.0.js
species
199
14

9
360104
strain
node2326.members.0.js

3
4
198
species
node2327.members.0.js

subspecies
91353
1

1031752
1
node2329.members.0.js
strain

species
4
76517

node2331.members.0.js
strain
360107
4

3
197
species
node2332.members.0.js

196
5
species

1507806
5
node2334.members.0.js
subspecies

3
no rank
node2335.members.0.js
19
2321108

genus
7
2321115

7
663364
species
node2337.members.0.js

genus
9
28196

2
197482
species
node2339.members.0.js

2
28197
species
node2340.members.0.js

species
2
603050

node2342.members.0.js
strain
1032238
2

species
node2343.members.0.js
2
913109

node2344.members.0.js
species
28200
1

order
235899
2

224467
2
family

191301
1
genus

291048
1
species

391592
1
node2349.members.0.js
strain

191291
1
genus

2632186
1
no rank

1
2579250
species
node2352.members.0.js

class
2
580370

order
580371
2

family
580372
2

genus
377315
2

1921086
2
node2357.members.0.js
species

5
2008785
class

5
119069
order

family
206349
5

70774
5
genus

5
297
species
node2362.members.0.js

node2363.members.0.js
class
28216
55465
1361

33809
14
no rank

node2365.members.0.js
species
1904640
12

species
node2366.members.0.js
2
543913

29
206389
299
node2367.members.0.js
order

2008795
17
family

17
73029
genus

no rank
12
2633351

species
node2371.members.0.js
12
2231055

5
259537
species

159087
5
node2373.members.0.js
strain

family
75787
74

13
551759
genus

species
551760
13

76114
13
node2377.members.0.js
strain

genus
1911577
11

node2379.members.0.js
species
1735038
11

1
4
75788
no rank
node2380.members.0.js

3
1898103
species
node2381.members.0.js

18
genus
node2382.members.0.js
46
146937

2609269
12
no rank

12
1765049
species
node2384.members.0.js

16
146939
species

node2386.members.0.js
strain
640081
16

4
family
node2387.members.0.js
179
2008794

no rank
2080468
13

2080469
13
node2389.members.0.js
species

genus
node2390.members.0.js
54
33057
7

species
node2391.members.0.js
2
96773

node2392.members.0.js
species
1134435
13

29
2609274
no rank

species
node2394.members.0.js
16
85643

13
2005884
species
node2395.members.0.js

59405
2
species

node2397.members.0.js
strain
44139
2

1
2184083
species
node2398.members.0.js

10
108
12960
genus
node2399.members.0.js

node2400.members.0.js
species
41977
8

node2401.members.0.js
no rank
2629479
79
13

5
62928
species
node2402.members.0.js

node2403.members.0.js
species
748247
9

species
node2404.members.0.js
9
198107

21
356837
species
node2405.members.0.js

species
node2406.members.0.js
18
2067960

4
2027405
species
node2407.members.0.js

11
418699
species
node2408.members.0.js

33
order
node2409.members.0.js
1260
32003

64
32011
1048
node2410.members.0.js
family

9
359407
genus

species
5
1055487

strain
node2413.members.0.js
5
666681

species
359408
4

583345
4
node2415.members.0.js
strain

13
16
genus

species
node2417.members.0.js
2
2588534

no rank
2630143
11

1662285
11
node2419.members.0.js
species

404
109
genus

species
405
109

node2422.members.0.js
strain
265072
109

544
node2423.members.0.js
genus
81682
839

no rank
123
2634027

species
node2425.members.0.js
123
887061

species
266009
172

172
582744
strain
node2427.members.0.js

genus
node2428.members.0.js
14
1679002
1

12
2588536
species
node2429.members.0.js

node2430.members.0.js
species
1581680
1

family
24
90627

96
2
genus

2
370405
species

395494
2
node2434.members.0.js
strain

genus
314343
9

9
63745
species

580332
9
node2437.members.0.js
strain

935200
1
genus

species
1
649841

1163617
1
node2440.members.0.js
strain

genus
1443590
1

node2442.members.0.js
species
1188319
1

11
1778653
genus

no rank
2637784
4

node2445.members.0.js
species
1985873
4

node2446.members.0.js
species
171865
7

19
206379
family

5
35798
genus

1231
4
species

strain
node2450.members.0.js
4
323848

species
1
35799

1
1266925
strain
node2452.members.0.js

4
genus
node2453.members.0.js
14
914

species
2
916

strain
node2455.members.0.js
2
335283

species
915
6

strain
node2457.members.0.js
6
228410

44577
2
node2458.members.0.js
species

family
24
2008790

1938335
10
genus

10
1914471
species
node2461.members.0.js

14
919
genus

14
36861
species

14
292415
strain
node2464.members.0.js

112
2008793
family
node2465.members.0.js
3

1054211
8
genus

8
748811
species

1223802
8
node2468.members.0.js
strain

genus
378210
69

no rank
69
2639971

species
node2471.members.0.js
69
1842540

157591
7
genus

157592
7
node2473.members.0.js
species

2211107
16
no rank

2496847
10
node2475.members.0.js
species

node2476.members.0.js
species
2211108
6

genus
311181
9

no rank
2609272
9

species
node2479.members.0.js
9
1981880

no rank
119066
26

327159
1
genus

species
327160
1

node2483.members.0.js
strain
522306
1

8
33055
genus

species
994692
8

8
1208919
strain
node2486.members.0.js

17
2705999
family

genus
2706000
17

2591109
17
node2489.members.0.js
species

9
node2490.members.0.js
order
206351
560

family
node2491.members.0.js
152
1499392
3

8
187
genus

no rank
8
2620219

1938604
8
node2494.members.0.js
species

885864
16
genus

16
2619593
no rank

16
1906741
species
node2497.members.0.js

node2498.members.0.js
genus
407217
18
2

16
2628611
no rank

species
node2500.members.0.js
8
1537400

8
1590041
species
node2501.members.0.js

568394
10
genus

2642558
10
no rank

species
node2504.members.0.js
10
748280

56
90153
no rank

32014
3
genus

no rank
3
235634

3
2496266
species
node2508.members.0.js

16
535
53
node2509.members.0.js
genus

species
node2510.members.0.js
16
1108595

node2511.members.0.js
species
536
9

12
2641838
no rank
node2512.members.0.js
2

node2513.members.0.js
species
2605945
4

node2514.members.0.js
species
2202142
1

2059672
5
node2515.members.0.js
species

genus
21
57739

21
2684990
no rank

species
node2518.members.0.js
21
1192162

4
57479
genus

57480
4
node2520.members.0.js
species

16
168470
genus

168471
16
node2522.members.0.js
species
14

557598
2
node2523.members.0.js
strain

7
481
399
node2524.members.0.js
family

genus
1654931
5

species
node2526.members.0.js
5
2290923

1
421605
no rank

node2528.members.0.js
species
2052837
1

genus
37
59

node2530.members.0.js
species
63
35

no rank
2627922
2

96942
2
node2532.members.0.js
species

genus
1055692
9

node2534.members.0.js
species
682798
9

genus
538
15

species
node2536.members.0.js
15
539

482
325
node2537.members.0.js
genus
114

487
1
node2538.members.0.js
species

species
node2539.members.0.js
56
488

node2540.members.0.js
species
483
1

1
493
species
node2541.members.0.js

71
28449
species
node2542.members.0.js

1853278
7
node2543.members.0.js
species

4
28091
species
node2544.members.0.js

4
486
species
node2545.members.0.js

18
2623750
no rank

species
node2547.members.0.js
7
655307

641148
11
species

641149
11
node2549.members.0.js
strain

species
node2550.members.0.js
30
484

node2551.members.0.js
species
495
18
15

node2552.members.0.js
subspecies
88719
3

3395
51945
80840
order
node2553.members.0.js

1174
28210
119060
family
node2554.members.0.js

genus
23
44013

species
node2556.members.0.js
4
556054

species
node2557.members.0.js
2
1743168

1835254
6
node2558.members.0.js
species

1
8
576610
species
node2559.members.0.js

7
452638
strain
node2560.members.0.js

species
node2561.members.0.js
2
576611

node2562.members.0.js
species
2527775
1

genus
7
240411

node2564.members.0.js
species
2594795
7

51
348
1822464
genus
node2565.members.0.js

node2566.members.0.js
species
134536
5

21
948107
species

node2568.members.0.js
strain
754502
21

species
252970
1

1229205
1
node2570.members.0.js
strain

15
node2571.members.0.js
species
75105
21

strain
node2572.members.0.js
6
1323664

node2573.members.0.js
species
169427
14

36873
6
species

266265
6
node2575.members.0.js
strain

2026199
4
node2576.members.0.js
species

node2577.members.0.js
species
60548
7

node2578.members.0.js
species
169430
5

311230
4
node2579.members.0.js
species

species
node2580.members.0.js
8
2211211

species
23
148447

391038
23
node2582.members.0.js
strain

11
1761016
species
node2583.members.0.js

11
420953
species
node2584.members.0.js

species
node2585.members.0.js
87
134537

species
261302
12

node2587.members.0.js
strain
398527
12

2
node2588.members.0.js
no rank
2615204
57

node2589.members.0.js
species
640511
18

node2590.members.0.js
species
2547399
8

11
2571748
species
node2591.members.0.js

1926494
7
node2592.members.0.js
species

species
node2593.members.0.js
11
2571747

genus
15
1827195

15
2646786
no rank

15
2705547
species
node2596.members.0.js

genus
47670
136

node2598.members.0.js
species
47671
136

93217
151
node2599.members.0.js
genus
39

species
node2600.members.0.js
13
93219

species
node2601.members.0.js
4
656178

species
node2602.members.0.js
28
445709

1891094
4
node2603.members.0.js
species

species
node2604.members.0.js
5
93218

species
node2605.members.0.js
7
93222

8
573737
species
node2606.members.0.js

93220
20
node2607.members.0.js
species

no rank
2624094
5

species
node2609.members.0.js
5
2518599

node2610.members.0.js
species
656179
4

species
node2611.members.0.js
14
93221

genus
1810868
2

species
node2613.members.0.js
2
1553431

1048
node2614.members.0.js
genus
48736
7727

190721
530
node2615.members.0.js
species

species
node2616.members.0.js
115
105219

329
5989
node2617.members.0.js
species
1819

428406
4066
node2618.members.0.js
strain

node2619.members.0.js
strain
402626
104

node2620.members.0.js
species
305
43
39

node2621.members.0.js
strain
859655
4

2
209769
no rank

node2623.members.0.js
species
1944648
2

2571159
4
genus

species
412963
4

strain
node2626.members.0.js
4
882378

2680021
3
genus

species
node2628.members.0.js
3
2268024

136
genus
node2629.members.0.js
1081
106589

1796606
5
node2630.members.0.js
species

68895
26
node2631.members.0.js
species

no rank
2640874
9

9
876364
species
node2633.members.0.js

248026
12
species

264198
12
node2635.members.0.js
strain

node2636.members.0.js
species
82633
44

species
82541
49

49
1267562
strain
node2638.members.0.js

90
91
164546
species
node2639.members.0.js

node2640.members.0.js
strain
977880
1

119219
633
node2641.members.0.js
species

151783
18
node2642.members.0.js
species

19
106590
31
node2643.members.0.js
species

strain
node2644.members.0.js
10
1042878

node2645.members.0.js
strain
381666
2

species
node2646.members.0.js
27
96344

node2647.members.0.js
genus
32008
17539
3040

node2648.members.0.js
species
41899
17

node2649.members.0.js
species
758793
9

node2650.members.0.js
species
28095
41
34

7
32009
no rank
node2651.members.0.js

4957
species group
node2652.members.0.js
14265
87882

1503055
7
node2653.members.0.js
species

species
node2654.members.0.js
18
1503054

node2655.members.0.js
species
179879
2

node2656.members.0.js
species
265293
4

87883
63
node2657.members.0.js
species

species
node2658.members.0.js
8
60550

species
node2659.members.0.js
26
482957

9073
488447
species
node2660.members.0.js

node2661.members.0.js
species
60552
2

species
3
152480

strain
node2663.members.0.js
2
398577

339670
1
node2664.members.0.js
strain

species
node2665.members.0.js
3
488732

species
node2666.members.0.js
1
488446

488731
6
node2667.members.0.js
species

node2668.members.0.js
species
1207504
2

17
species
node2669.members.0.js
27
292

1009846
9
node2670.members.0.js
strain

node2671.members.0.js
strain
1395570
1

1
488729
species
node2672.members.0.js

18
19
101571
species
node2673.members.0.js

node2674.members.0.js
strain
1249668
1

95486
23
node2675.members.0.js
species
22

strain
node2676.members.0.js
1
216591

node2677.members.0.js
species
95485
20

337
16
node2678.members.0.js
species

2
85
2613784
no rank
node2679.members.0.js

species
node2680.members.0.js
3
2217913

19
1705310
species
node2681.members.0.js

758782
10
node2682.members.0.js
species

640512
8
node2683.members.0.js
species

node2684.members.0.js
species
1795043
1

1740163
1
node2685.members.0.js
species

species
node2686.members.0.js
23
1855726

1637862
6
node2687.members.0.js
species

1637869
1
node2688.members.0.js
species

node2689.members.0.js
species
2571746
11

111527
66
node2690.members.0.js
species group
27

species
node2691.members.0.js
1
13373

1385591
6
node2692.members.0.js
species

57975
12
node2693.members.0.js
species

species
node2694.members.0.js
1
1385592

node2695.members.0.js
species
1637831
1

4
342113
species
node2696.members.0.js

node2697.members.0.js
species
28450
14
11

node2698.members.0.js
strain
1249475
2

strain
node2699.members.0.js
1
1435365

80864
16200
node2700.members.0.js
family
2589

106
219181
genus
node2701.members.0.js
1

2109914
48
node2702.members.0.js
species

no rank
57
2645081

node2704.members.0.js
species
1658672
57

genus
93
1649468

93
2641902
no rank

93
2109913
species
node2707.members.0.js

179
node2708.members.0.js
genus
12916
1197

80868
20
node2709.members.0.js
species

73
node2710.members.0.js
no rank
2684926
757

88
2478662
species
node2711.members.0.js

species
node2712.members.0.js
132
1842533

species
node2713.members.0.js
63
1858609

358220
330
node2714.members.0.js
species

71
232721
species
node2715.members.0.js

721785
11
species

strain
node2717.members.0.js
11
535289

species
91
80867

80870
91
node2719.members.0.js
subspecies
90

1
643561
strain
node2720.members.0.js

node2721.members.0.js
species
80869
35

species
node2722.members.0.js
104
553814

node2723.members.0.js
genus
2490452
96
8

53
1458426
species
node2724.members.0.js

1458425
35
node2725.members.0.js
species

5097
genus
node2726.members.0.js
7001
80865

180282
716
node2727.members.0.js
species

198
no rank
node2728.members.0.js
661
2613839

240
742013
species
node2729.members.0.js

species
node2730.members.0.js
215
1920191

species
node2731.members.0.js
8
1170710

80866
527
node2732.members.0.js
species
277

strain
node2733.members.0.js
250
398578

1436289
6
genus

6
1436290
species

strain
node2736.members.0.js
6
946483

genus
77
665874

no rank
2626134
77

node2739.members.0.js
species
1678129
20

species
node2740.members.0.js
57
1678128

433
47420
genus
node2741.members.0.js
39

node2742.members.0.js
species
47421
33

node2743.members.0.js
species
1763535
26

30
2610897
335
node2744.members.0.js
no rank

54
795665
species
node2745.members.0.js

2565558
28
node2746.members.0.js
species

node2747.members.0.js
species
434010
51

61
2651974
species
node2748.members.0.js

75
1842537
species
node2749.members.0.js

species
node2750.members.0.js
36
2184519

genus
225
364316

species
225
364317

225
391735
strain
node2753.members.0.js

10
28065
259
node2754.members.0.js
genus

species
node2755.members.0.js
29
1484693

no rank
node2756.members.0.js
23
2627954
1

node2757.members.0.js
species
2527691
22

node2758.members.0.js
species
81479
20

57
192843
species

node2760.members.0.js
strain
338969
57

59
1842727
species
node2761.members.0.js

61
2509614
species
node2762.members.0.js

283
1057
node2763.members.0.js
genus
122

37
32013
species

strain
node2765.members.0.js
37
1219032

species
node2766.members.0.js
50
1082851

node2767.members.0.js
species
225992
68

node2768.members.0.js
species
363952
9

2638500
54
no rank

species
node2770.members.0.js
54
2597701

119
285
species
node2771.members.0.js
59

1392005
45
node2772.members.0.js
strain

strain
node2773.members.0.js
15
1191062

598
225991
species
node2774.members.0.js

genus
201096
83

83
179636
species
node2776.members.0.js
46

strain
node2777.members.0.js
25
596154

596153
12
node2778.members.0.js
strain

genus
35
281915

no rank
35
2685271

35
1844971
species
node2781.members.0.js

577
genus
node2782.members.0.js
2209
34072

97
436515
species
node2783.members.0.js

220
species
node2784.members.0.js
420
34073

595537
78
node2785.members.0.js
strain

47
1246301
strain
node2786.members.0.js

node2787.members.0.js
strain
543728
75

266
1115
663243
no rank
node2788.members.0.js

node2789.members.0.js
species
2126319
363

662548
49
node2790.members.0.js
species

21
282217
species
node2791.members.0.js

species
node2792.members.0.js
110
1795631

30
434014
species
node2793.members.0.js

1034889
112
node2794.members.0.js
species

species
node2795.members.0.js
49
434009

79
434008
species
node2796.members.0.js

207745
36
node2797.members.0.js
species

174951
198
genus

75
node2799.members.0.js
species
94132
198

123
365046
strain
node2800.members.0.js

238749
18
genus

node2802.members.0.js
species
1546149
18

52972
321
node2803.members.0.js
genus
10

216465
92
species

92
365044
strain
node2805.members.0.js

76
no rank
node2806.members.0.js
219
2638319

296591
143
node2807.members.0.js
species

68
352450
genus

2109915
68
node2809.members.0.js
species

232523
53
genus

species
node2811.members.0.js
53
80880

genus
76
2678886

76
2116657
species
node2813.members.0.js

4
no rank
node2814.members.0.js
103
80841

node2815.members.0.js
species
864051
82

1469502
17
node2816.members.0.js
species

48
family
node2817.members.0.js
660
506

genus
10
305976

10
2640016
no rank

node2820.members.0.js
species
2028345
2

species
node2821.members.0.js
8
1007105

genus
12
257820

species
node2823.members.0.js
12
206506

1
106146
genus

no rank
1
2652175

2652177
1
node2826.members.0.js
species

62
node2827.members.0.js
genus
517
247

14
463025
species
node2828.members.0.js

species
node2829.members.0.js
8
463014

10
123899
species
node2830.members.0.js

15
1416803
species
node2831.members.0.js

9
103855
species
node2832.members.0.js

node2833.members.0.js
no rank
2630031
43
1

node2834.members.0.js
species
1697043
19

1746199
19
node2835.members.0.js
species

2163011
4
node2836.members.0.js
species

species
node2837.members.0.js
2
518

species
node2838.members.0.js
13
1416806

1331258
19
node2839.members.0.js
species

94624
18
node2840.members.0.js
species

node2841.members.0.js
species
35814
16

species
node2842.members.0.js
6
463040

12
521
species
node2843.members.0.js

no rank
83496
27

node2845.members.0.js
species
2593958
27

genus
359336
18

species
18
75697

18
1437824
strain
node2848.members.0.js

10
290425
genus

302406
1
species

strain
node2851.members.0.js
1
1247726

species
310575
9

1036672
9
node2853.members.0.js
strain

node2854.members.0.js
genus
507
36
1

node2855.members.0.js
species
511
34

species
node2856.members.0.js
1
323284

211
222
genus
node2857.members.0.js
47

no rank
2626865
43

node2859.members.0.js
species
2282475
18

node2860.members.0.js
species
1758194
12

13
1881016
species
node2861.members.0.js

19
32002
species
node2862.members.0.js

72557
8
node2863.members.0.js
species

node2864.members.0.js
species
217204
10

species
node2865.members.0.js
23
217203

61
85698
species
node2866.members.0.js
49

strain
node2867.members.0.js
12
762376

genus
152267
26

no rank
19
2626614

2488560
19
node2870.members.0.js
species

1940612
7
node2871.members.0.js
species

14
1921582
genus

node2873.members.0.js
species
1851544
14

143
family
node2874.members.0.js
2045
75682

29
29580
130
node2875.members.0.js
genus

35
55508
species
node2876.members.0.js
19

node2877.members.0.js
strain
1349767
16

node2878.members.0.js
species
368607
4

2610881
62
no rank

1644131
20
node2880.members.0.js
species

9
375286
species
node2881.members.0.js

1
1938606
species
node2882.members.0.js

node2883.members.0.js
species
2590869
10

species
node2884.members.0.js
20
2497863

2
1236179
species
node2885.members.0.js

genus
29
303379

204773
27
node2887.members.0.js
species

species
node2888.members.0.js
2
1809410

genus
23
401469

no rank
node2890.members.0.js
21
2630295
6

8
2058624
species
node2891.members.0.js

7
2058625
species
node2892.members.0.js

node2893.members.0.js
species
401471
2

26
963
172
node2894.members.0.js
genus

40
2014887
species
node2895.members.0.js

node2896.members.0.js
species
964
27

80842
28
node2897.members.0.js
species

no rank
2624150
10

2025949
10
node2899.members.0.js
species

species
341045
10

1262470
10
node2901.members.0.js
strain

species
node2902.members.0.js
31
863372

2
node2903.members.0.js
genus
202907
50

27
node2904.members.0.js
species
158899
33

strain
node2905.members.0.js
6
1005048

species
node2906.members.0.js
8
279058

node2907.members.0.js
species
279113
7

genus
1344552
10

no rank
10
2617509

species
node2910.members.0.js
10
2601898

165
149698
1488
node2911.members.0.js
genus

species
node2912.members.0.js
24
2072590

21
871742
species
node2913.members.0.js

node2914.members.0.js
species
2045208
56

node2915.members.0.js
species
321983
25

864828
27
node2916.members.0.js
species

node2917.members.0.js
species
321984
44

node2918.members.0.js
species
945844
911

node2919.members.0.js
species
1141883
80

2609279
103
no rank

species
node2921.members.0.js
66
1707785

37
1678028
species
node2922.members.0.js

node2923.members.0.js
species
321985
32

family
4
995019

4
40544
genus

2584944
4
node2926.members.0.js
species

152
224471
1328
node2927.members.0.js
no rank

genus
92793
238

species
node2929.members.0.js
238
1296669

318147
74
genus

no rank
2642959
74

74
1768242
species
node2932.members.0.js

genus
74
93681

node2934.members.0.js
species
76731
74

88
58
genus

34029
58
species

node2937.members.0.js
strain
395495
58

47
116
316612
genus
node2938.members.0.js

species
105560
34

34
420662
strain
node2940.members.0.js

15
node2941.members.0.js
no rank
2633235
35

species
node2942.members.0.js
20
2082386

64
644355
genus

64
392593
species
node2944.members.0.js

75
28067
genus

species
28068
75

strain
node2947.members.0.js
75
983917

65047
67
genus

no rank
2648776
67

67
1658665
species
node2950.members.0.js

54066
85
genus

no rank
2646274
85

species
node2953.members.0.js
85
2697032

genus
212743
42

node2955.members.0.js
species
946333
42

genus
129
34102

species
34103
129

129
639200
subspecies
node2958.members.0.js

species
node2959.members.0.js
57
413882

32
97
32012
genus
node2960.members.0.js

5
30
2625466
no rank
node2961.members.0.js

species
node2962.members.0.js
25
1050370

species
node2963.members.0.js
14
426114

species
node2964.members.0.js
21
926

378
32066
phylum

class
378
203490

203491
378
node2967.members.0.js
order
4

family
199
1129771

2755140
6
genus

species
node2970.members.0.js
6
157692

genus
32068
5

5
826
species

526218
5
node2973.members.0.js
strain

69
genus
node2974.members.0.js
186
32067

node2975.members.0.js
species
157688
1

no rank
26
2633022

node2977.members.0.js
species
712357
26

554406
15
node2978.members.0.js
species

39
109328
species
node2979.members.0.js

species
1
40542

1
523794
strain
node2981.members.0.js

35
157687
species
node2982.members.0.js

genus
2
168808

node2984.members.0.js
species
187101
2

family
175
203492

genus
node2986.members.0.js
175
848
54

node2987.members.0.js
species
851
111
24

subspecies
node2988.members.0.js
67
76857

76856
2
node2989.members.0.js
subspecies

subspecies
node2990.members.0.js
9
155615
1

8
1307427
strain
node2991.members.0.js

9
76859
subspecies
node2992.members.0.js

node2993.members.0.js
species
1583098
4
2

2
1307444
strain
node2994.members.0.js

species
2
850

2
469616
strain
node2996.members.0.js

node2997.members.0.js
species
861
4

phylum
51
203691

203692
51
class

3
1643686
order

family
3
143786

genus
3
29521

1
159
species
node3003.members.0.js

1287055
1
node3004.members.0.js
species

species
1
84377

1
1045858
strain
node3006.members.0.js

136
9
order

137
6
family

genus
157
2

species
81028
2

node3011.members.0.js
strain
906968
2

399320
4
genus

species
1131703
2

strain
node3014.members.0.js
2
158189

species
1131707
2

strain
node3016.members.0.js
2
158190

3
1643685
family

1
138
genus

species
1
140

strain
node3020.members.0.js
1
1313295

genus
node3021.members.0.js
2
64895
1

1476873
1
node3022.members.0.js
species

1643688
39
order

170
39
family

171
39
node3025.members.0.js
genus
2

species
173
1

1
766041
no rank
node3027.members.0.js

node3028.members.0.js
species
1137606
1

species
node3029.members.0.js
28
2564040

3
172
species

3
145259
no rank
node3031.members.0.js

408139
2
node3032.members.0.js
species

species
node3033.members.0.js
2
174

phylum
200783
9

class
187857
9

32069
9
order

224027
8
family

8
212790
genus

species
8
309806

204536
8
node3040.members.0.js
strain

64898
1
family

168657
1
genus

1
2622382
no rank
node3043.members.0.js

2
no rank
node3044.members.0.js
10
48479

species
node3045.members.0.js
4
795324

558689
4
node3046.members.0.js
species

phylum
200938
1

class
1
118001

order
1
189769

family
189770
1

genus
1
393029

936456
1
species

strain
node3053.members.0.js
1
653733

clade
42804
1783270

node3055.members.0.js
clade
68336
42766
16

phylum
1
1936987

no rank
1
2489366

1
2489367
genus

1
1457365
species
node3059.members.0.js

1090
26
phylum

26
191410
class

order
191411
26

191412
26
family

6
256319
genus

species
2
1097

node3066.members.0.js
strain
194439
2

1
274537
species
node3067.members.0.js

species
3
274539

node3069.members.0.js
strain
517417
3

genus
5
1101

2632826
5
no rank

1868325
2
node3072.members.0.js
species

3
281093
species
node3073.members.0.js

no rank
13
274493

genus
1091
9

3
337090
species

strain
node3077.members.0.js
3
340177

species
1092
3

290315
3
node3079.members.0.js
strain

species
1096
3

290317
3
node3081.members.0.js
strain

1099
4
genus

1100
4
species

4
319225
strain
node3084.members.0.js

2
100715
genus

100716
2
species

2
517418
strain
node3087.members.0.js

node3088.members.0.js
phylum
976
42720
380

order
25
1100069

1
25
563843
family
node3090.members.0.js

no rank
node3091.members.0.js
10
1196022
4

4
1779382
species
node3092.members.0.js

2
2026787
species
node3093.members.0.js

genus
146918
13

species
node3095.members.0.js
13
146919

genus
1
29548

node3097.members.0.js
species
29549
1

class
768503
38615

38615
768507
order
node3099.members.0.js
116

129
1853232
1235
node3100.members.0.js
family

genus
node3101.members.0.js
70
323449
5

30
2648980
no rank

node3103.members.0.js
species
2694930
6

11
2571030
species
node3104.members.0.js

species
node3105.members.0.js
13
2694929

400092
15
node3106.members.0.js
species

node3107.members.0.js
species
323450
5

15
388950
species
node3108.members.0.js

genus
88
1649474

88
2646046
no rank

species
node3111.members.0.js
88
2698458

40
1379908
533
node3112.members.0.js
genus

5
no rank
node3113.members.0.js
330
2639626

1379910
243
node3114.members.0.js
species

82
1379909
species
node3115.members.0.js

512763
163
node3116.members.0.js
species

82
89966
415
node3117.members.0.js
genus

node3118.members.0.js
species
2319843
14

2502781
21
node3119.members.0.js
species

1411621
20
node3120.members.0.js
species

65
1446467
species

65
1227739
strain
node3122.members.0.js

species
node3123.members.0.js
8
1850093

2615202
205
node3124.members.0.js
no rank
30

node3125.members.0.js
species
2615203
13

23
2496028
species
node3126.members.0.js

15
2584940
species
node3127.members.0.js

node3128.members.0.js
species
1484118
15

12
1385663
species
node3129.members.0.js

species
node3130.members.0.js
45
1356852

node3131.members.0.js
species
1385664
14

2697562
14
node3132.members.0.js
species

1484116
24
node3133.members.0.js
species

1124781
13
no rank

1433993
13
genus

2321403
13
node3136.members.0.js
species

200667
12
family

no rank
340671
3

node3139.members.0.js
species
1257021
3

genus
6
869806

species
6
1006

strain
node3142.members.0.js
6
643867

genus
446458
1

node3144.members.0.js
species
1267423
1

genus
2
59739

2
2637820
no rank

1191459
2
node3147.members.0.js
species

203
family
node3148.members.0.js
37210
89373

37
105
genus

2631759
37
no rank

species
node3151.members.0.js
13
2268026

2259595
24
node3152.members.0.js
species

genus
25
120831

species
10
94254

471854
10
node3155.members.0.js
strain

15
2625061
no rank

species
node3157.members.0.js
15
538966

genus
5
2676060

998844
5
node3159.members.0.js
species

1
978
genus

species
1
985

strain
node3162.members.0.js
1
269798

19
455076
genus

19
2704464
no rank

node3165.members.0.js
species
2704465
19

9
861914
125
node3166.members.0.js
genus

species
651143
71

node3168.members.0.js
strain
1166018
71

no rank
45
2620963

node3170.members.0.js
species
1834519
45

1203
node3171.members.0.js
genus
107
36795

1178516
152
node3172.members.0.js
species

679
2621999
no rank
node3173.members.0.js
21

237
2666025
species
node3174.members.0.js

species
node3175.members.0.js
421
2520506

392
2057025
species
node3176.members.0.js

253
1379870
species
node3177.members.0.js

species
node3178.members.0.js
34024
1211326

92
564064
species
node3179.members.0.js

family
563798
29

genus
390846
16

species
node3182.members.0.js
3
1807691

species
node3183.members.0.js
1
1795355

4
2621165
no rank

2591634
4
node3185.members.0.js
species

390884
8
species

strain
node3187.members.0.js
8
926556

2
246875
genus

species
node3189.members.0.js
2
388413

11
280472
genus

species
280473
11

11
758820
strain
node3192.members.0.js

1118
117743
class

9
200644
1118
node3194.members.0.js
order

1099
49546
family
node3195.members.0.js
166

genus
111500
2

111501
2
species

886377
2
node3198.members.0.js
strain

10
node3199.members.0.js
genus
308865
42

1
1756149
species
node3200.members.0.js

6
2685307
no rank

node3202.members.0.js
species
2583851
2

4
2575699
species
node3203.members.0.js

species
node3204.members.0.js
19
172045

node3205.members.0.js
species
1756150
2

4
1117645
species
node3206.members.0.js

4
291183
genus

3
2647285
no rank

node3209.members.0.js
species
2057808
3

1486034
1
node3210.members.0.js
species

3
1013
genus

species
node3212.members.0.js
3
1014

3
252306
genus

3
252307
species

313596
3
node3215.members.0.js
strain

genus
59734
4

node3217.members.0.js
species
247
4

genus
252356
10

node3219.members.0.js
species
1178778
1

2
9
2615042
no rank
node3220.members.0.js

species
node3221.members.0.js
1
1644130

313603
4
node3222.members.0.js
species

species
node3223.members.0.js
2
2496865

genus
336276
2

639310
2
node3225.members.0.js
species

6
225842
genus

no rank
2644710
4

2
2686363
species
node3228.members.0.js

node3229.members.0.js
species
1798225
2

320324
2
species

1347342
2
node3231.members.0.js
strain

genus
2
1649495

2622645
2
no rank

1936081
2
node3234.members.0.js
species

13
104267
genus

1850252
5
node3236.members.0.js
species

species
node3237.members.0.js
3
584609

no rank
2635139
5

species
node3239.members.0.js
5
2358479

genus
358023
1

species
node3241.members.0.js
1
1622118

1016
120
node3242.members.0.js
genus
38

1019
36
node3243.members.0.js
species

327575
11
node3244.members.0.js
species

2708117
2
node3245.members.0.js
species

node3246.members.0.js
species
28188
2

species
node3247.members.0.js
3
45243

2
1848904
species
node3248.members.0.js

12
1017
species
node3249.members.0.js

no rank
14
2640652

7
1316596
species
node3251.members.0.js

7
2545799
species
node3252.members.0.js

2
2700084
genus

node3254.members.0.js
species
1850246
2

genus
4
286104

no rank
2615021
4

1249933
2
node3257.members.0.js
species

1936080
2
node3258.members.0.js
species

genus
2045416
2

no rank
2631961
2

2529032
2
node3261.members.0.js
species

genus
7
2058174

no rank
2631190
2

node3264.members.0.js
species
2585771
2

species
node3265.members.0.js
5
2058175

417127
1
genus

1
398743
species

1
655815
strain
node3268.members.0.js

261827
4
genus

no rank
2615009
4

4
2686366
species
node3271.members.0.js

527198
4
genus

2643887
4
no rank

4
2027857
species
node3274.members.0.js

28
501783
genus

node3276.members.0.js
species
237258
28

34084
7
genus

species
node3278.members.0.js
7
34085

9
237
116
node3279.members.0.js
genus

2
1763534
species
node3280.members.0.js

5
1355330
species
node3281.members.0.js

species
1
55197

1
1034807
strain
node3283.members.0.js

node3284.members.0.js
species
2201181
2

5
96345
species
node3285.members.0.js

683124
2
node3286.members.0.js
species

8
986
11
node3287.members.0.js
species

376686
3
node3288.members.0.js
strain

1
196869
43
node3289.members.0.js
no rank

4
2249356
species
node3290.members.0.js

species
node3291.members.0.js
2
2478552

node3292.members.0.js
species
2294119
5

1179672
8
node3293.members.0.js
species

23
935222
species
node3294.members.0.js

2175091
6
node3295.members.0.js
species

2183896
1
node3296.members.0.js
species

species
node3297.members.0.js
3
1678728

1
2518177
species
node3298.members.0.js

species
node3299.members.0.js
20
1306519

node3300.members.0.js
species
459526
2

species
node3301.members.0.js
2
1617283

species
312277
1

strain
node3303.members.0.js
1
1094466

genus
2
244698

2
2615025
no rank

species
node3306.members.0.js
2
1336796

4
290174
genus

no rank
4
2627091

species
node3309.members.0.js
2
1714848

2
1714849
species
node3310.members.0.js

no rank
37
61432

node3312.members.0.js
species
531844
28

2584122
3
node3313.members.0.js
species

species
node3314.members.0.js
4
2583587

1871037
2
node3315.members.0.js
species

143222
2
genus

node3317.members.0.js
species
143223
2

4
13
76831
genus
node3318.members.0.js

species
node3319.members.0.js
1
702745

species
256
4

strain
node3321.members.0.js
4
929704

node3322.members.0.js
species
480520
4

59732
436
node3323.members.0.js
genus
75

42
no rank
node3324.members.0.js
153
2593645

10
2487065
species
node3325.members.0.js

1871047
2
node3326.members.0.js
species

species
node3327.members.0.js
2
2594269

1
1721091
species
node3328.members.0.js

node3329.members.0.js
species
2713414
4

species
node3330.members.0.js
21
2487071

node3331.members.0.js
species
2478663
7

node3332.members.0.js
species
878220
2

7
2487072
species
node3333.members.0.js

2487073
22
node3334.members.0.js
species

2039166
24
node3335.members.0.js
species

2547600
5
node3336.members.0.js
species

2
2015076
species
node3337.members.0.js

node3338.members.0.js
species
2487063
2

node3339.members.0.js
species
1241982
2

node3340.members.0.js
species
253
12

558152
1
node3341.members.0.js
species

species
node3342.members.0.js
3
266749

6
651561
species
node3343.members.0.js

4
1265445
species
node3344.members.0.js

1241981
1
node3345.members.0.js
species

species
node3346.members.0.js
7
1324352

species
node3347.members.0.js
9
246

node3348.members.0.js
species
536441
32

species
node3349.members.0.js
12
1241979

12
1241978
species
node3350.members.0.js

node3351.members.0.js
species
254
22

250
33
node3352.members.0.js
species

1493872
12
node3353.members.0.js
species

421525
32
node3354.members.0.js
species

112234
3
node3355.members.0.js
species

4
266748
species
node3356.members.0.js

1
2497456
species
node3357.members.0.js

15
292691
genus

1486245
7
species

1229726
7
node3360.members.0.js
strain

species
411153
4

4
411154
strain
node3362.members.0.js

2126553
4
node3363.members.0.js
species

genus
1
393005

no rank
1
2614803

1
2069432
species
node3366.members.0.js

genus
379070
3

3
2625242
no rank

754429
3
node3369.members.0.js
species

153265
4
genus

3
2615031
no rank

3
2494375
species
node3372.members.0.js

101385
1
species

strain
node3374.members.0.js
1
746697

genus
59735
4

1585976
4
node3376.members.0.js
species

1
112040
genus

63186
1
node3378.members.0.js
species

104264
3
genus

59600
3
species

strain
node3381.members.0.js
3
688270

genus
4
1287916

1761453
4
node3383.members.0.js
species

1862153
3
genus

1703343
3
node3385.members.0.js
species

genus
3
221065

species
node3387.members.0.js
3
1218801

1
genus
node3388.members.0.js
10
52959

3
996801
species
node3389.members.0.js

196858
6
no rank

754397
2
node3391.members.0.js
species

1
2686361
species
node3392.members.0.js

node3393.members.0.js
species
1896175
1

2
1336804
species
node3394.members.0.js

1209327
1
genus

node3396.members.0.js
species
1803846
1

no rank
1
313602

genus
1
1940138

1415657
1
node3399.members.0.js
species

family
2
246874

genus
267986
2

species
2
253245

node3403.members.0.js
strain
926562
2

family
5
1755828

genus
5
1755829

node3406.members.0.js
species
242600
5

family
2
1853230

332102
2
genus

species
191579
2

2
755732
strain
node3410.members.0.js

1937959
7
class

order
7
1936988

family
1937961
7

genus
2349
7

species
7
2350

7
760192
strain
node3416.members.0.js

class
1953
117747

200666
1953
order

84566
1953
node3419.members.0.js
family
15

no rank
84568
9

node3421.members.0.js
species
1986952
9

49
423349
genus
node3422.members.0.js
3

node3423.members.0.js
species
1550579
3

node3424.members.0.js
species
1234841
5

node3425.members.0.js
species
652787
6

no rank
11
2617802

node3427.members.0.js
species
2305508
10

node3428.members.0.js
species
1300914
1

node3429.members.0.js
species
398053
13

node3430.members.0.js
species
862126
2

node3431.members.0.js
species
2027860
6

genus
1649482
1

species
1
151895

node3434.members.0.js
strain
762903
1

376469
8
genus

no rank
8
2632301

8
2592345
species
node3437.members.0.js

28453
1838
node3438.members.0.js
genus
128

no rank
node3439.members.0.js
1063
2609468
12

node3440.members.0.js
species
2662364
12

species
node3441.members.0.js
8
2003121

species
node3442.members.0.js
5
1538644

node3443.members.0.js
species
2557994
1026

23
797291
species
node3444.members.0.js

species
node3445.members.0.js
2
561061

190
649196
species
node3446.members.0.js

1010
13
node3447.members.0.js
species

species
node3448.members.0.js
2
259

species
node3449.members.0.js
417
371142

84567
33
node3450.members.0.js
genus
1

species
node3451.members.0.js
1
363852

no rank
2628915
18

2
2201271
species
node3453.members.0.js

species
node3454.members.0.js
6
2578106

2482728
5
node3455.members.0.js
species

2605747
1
node3456.members.0.js
species

species
node3457.members.0.js
4
1727164

node3458.members.0.js
species
332999
2

984
6
species

node3460.members.0.js
strain
485917
6

2
430522
species
node3461.members.0.js

node3462.members.0.js
species
188932
3

504
200643
class

order
11
1970189

family
1471398
10

no rank
2
1952944

species
node3467.members.0.js
2
2681766

1471399
8
genus

no rank
8
2624132

8
2706887
species
node3470.members.0.js

1970190
1
family

1
1970191
genus

species
node3473.members.0.js
1
1307839

24
node3474.members.0.js
order
171549
493

171552
184
node3475.members.0.js
family
2

genus
1
577309

species
1
454155

strain
node3478.members.0.js
1
762982

181
838
genus
node3479.members.0.js
25

species
node3480.members.0.js
38
28132

589436
3
species

1236517
3
node3482.members.0.js
strain

node3483.members.0.js
species
28129
45
42

node3484.members.0.js
strain
767031
3

12
28135
species
node3485.members.0.js

76123
4
node3486.members.0.js
species

no rank
2638335
6

species
652716
6

node3489.members.0.js
strain
575614
6

node3490.members.0.js
species
1177574
14

52227
15
species

908937
15
node3492.members.0.js
strain

species
839
5

strain
node3494.members.0.js
5
264731

14
28131
species
node3495.members.0.js

20
171550
family

20
239759
genus

species
node3498.members.0.js
5
2585119

6
328813
species

subspecies
node3500.members.0.js
6
2585117

species
node3501.members.0.js
1
2585118

4
2364787
species
node3502.members.0.js

3
2608932
no rank

species
node3504.members.0.js
3
2662363

214856
1
species

679935
1
node3506.members.0.js
strain

25
171551
family

genus
836
19

837
4
node3509.members.0.js
species

28123
15
species

879243
15
node3511.members.0.js
strain

307628
6
genus

species
node3513.members.0.js
6
1642646

family
9
2005473

genus
9
1918540

no rank
2
2622126

2
2530390
species
node3517.members.0.js

7
1796646
species
node3518.members.0.js

family
60
2005525

3
node3520.members.0.js
genus
375288
4

823
1
node3521.members.0.js
species

genus
56
195950

2620507
55
no rank

node3524.members.0.js
species
712710
55

28112
1
node3525.members.0.js
species

2005520
5
family

5
294702
genus

1642647
5
node3528.members.0.js
species

1853231
3
family

genus
3
283168

species
node3531.members.0.js
3
28118

7
2005523
family

genus
346096
7

species
7
185300

694427
7
node3535.members.0.js
strain

family
147
815

66
node3537.members.0.js
genus
816
147

820
2
node3538.members.0.js
species

2
290053
species

2
693979
strain
node3540.members.0.js

3
28116
species
node3541.members.0.js

node3542.members.0.js
species
357276
21

species
node3543.members.0.js
10
47678

1796613
2
node3544.members.0.js
species

node3545.members.0.js
species
818
7

species
node3546.members.0.js
2
28119

node3547.members.0.js
species
28113
5

node3548.members.0.js
species
817
2

3
371601
species
node3549.members.0.js

16
821
species
node3550.members.0.js
12

435590
4
node3551.members.0.js
strain

246787
6
node3552.members.0.js
species

2
185291
no rank

2
1400053
species
node3554.members.0.js

family
2005519
7

397864
7
genus

species
397865
7

node3558.members.0.js
strain
880074
7

1853228
118
class

1853229
118
order

7
563835
118
node3561.members.0.js
family

1
1860196
genus

1
661488
species
node3563.members.0.js

13
649460
genus

477680
13
node3565.members.0.js
species

genus
7
2698688

2315862
7
node3567.members.0.js
species

15
1769012
genus

no rank
2636138
15

node3570.members.0.js
species
2341117
3

species
node3571.members.0.js
4
2545455

node3572.members.0.js
species
1850526
8

genus
node3573.members.0.js
30
79328
1

species
node3574.members.0.js
6
79329
1

node3575.members.0.js
strain
485918
5

2619133
23
no rank

species
node3577.members.0.js
9
2604421

species
node3578.members.0.js
6
2033437

species
node3579.members.0.js
8
2703787

398041
30
genus

661481
7
node3581.members.0.js
species

2639558
23
no rank

species
node3583.members.0.js
23
2502779

genus
5
1004301

no rank
5
2621068

2676868
5
node3586.members.0.js
species

4
1874621
genus

1813871
4
node3588.members.0.js
species

genus
5
354354

species
5
354356

node3591.members.0.js
strain
700598
5

genus
1
379899

1176587
1
node3593.members.0.js
species

phylum
1134404
3

3
795747
class

order
795748
3

2
1334117
family

genus
1134403
2

species
1134405
2

node3600.members.0.js
strain
1191523
2

1
795749
family

795750
1
genus

1
591197
species

945713
1
node3604.members.0.js
strain

phylum
11
65842

204430
11
class

order
11
218872

11
204431
family

genus
832
11

833
11
species

834
11
subspecies

59374
11
node3612.members.0.js
strain

27
142182
phylum

class
219685
27

order
219686
27

1
family
node3616.members.0.js
27
219687

genus
13
173479

6
173480
species

379066
6
node3619.members.0.js
strain

species
node3620.members.0.js
7
1379270

1706036
13
genus

species
node3622.members.0.js
13
861299

clade
594
1783257

503
203682
phylum
node3624.members.0.js
34

10
237
203683
class
node3625.members.0.js

90
2691355
order

family
1914233
90

2731450
22
genus

2598579
22
node3629.members.0.js
species

1
2051044
genus

692036
1
node3631.members.0.js
species

11
genus
node3632.members.0.js
67
113

no rank
2622970
1

1
1630693
species
node3634.members.0.js

114
38
node3635.members.0.js
species

17
1210884
species
node3636.members.0.js

2691356
45
order

5
1763524
45
node3638.members.0.js
family

5
127
genus

species
5
128

575540
5
node3641.members.0.js
strain

genus
22
1511635

node3643.members.0.js
species
406548
22

1763521
10
genus

node3645.members.0.js
species
1387353
10

3
466152
genus

466153
3
species

886293
3
node3648.members.0.js
strain

4
node3649.members.0.js
order
112
60

6
family
node3650.members.0.js
51
126

genus
2714594
2

node3652.members.0.js
species
2527984
2

genus
1649490
4

species
119
4

strain
node3655.members.0.js
4
756272

10
2714737
genus

species
node3657.members.0.js
10
2527989

1649453
3
genus

3
2634125
no rank

2608982
3
node3660.members.0.js
species

genus
node3661.members.0.js
5
1649480
4

2528019
1
node3662.members.0.js
species

genus
118
21

21
2648843
no rank

10
1632864
species
node3665.members.0.js

11
1636152
species
node3666.members.0.js

5
466154
no rank

node3668.members.0.js
species
2603660
1

node3669.members.0.js
species
2053591
4

order
32
2691354

9
2691358
family

9
1676125
genus

node3673.members.0.js
species
1331910
9

10
2691359
family

no rank
4
2691521

4
2528024
species
node3676.members.0.js

genus
6
1400386

species
node3678.members.0.js
6
1400387

family
2691357
13

genus
6
265488

6
265606
species

243090
6
node3682.members.0.js
strain

genus
4
1579506

980254
4
node3684.members.0.js
species

123
3
genus

no rank
2639138
2

1632865
2
node3687.members.0.js
species

125
1
species

node3689.members.0.js
strain
530564
1

node3690.members.0.js
no rank
473814
216
31

2527963
7
node3691.members.0.js
species

5
2528026
species
node3692.members.0.js

species
node3693.members.0.js
2
2527995

species
node3694.members.0.js
10
2527976

1
2026780
species
node3695.members.0.js

species
node3696.members.0.js
9
2527998

1
2528007
species
node3697.members.0.js

2527979
5
node3698.members.0.js
species

2527974
27
node3699.members.0.js
species

1
1930273
species
node3700.members.0.js

4
2527975
species
node3701.members.0.js

node3702.members.0.js
species
2527992
1

species
node3703.members.0.js
4
2528021

node3704.members.0.js
species
2527983
6

2528008
1
node3705.members.0.js
species

2527982
2
node3706.members.0.js
species

2527972
4
node3707.members.0.js
species

8
2528018
species
node3708.members.0.js

species
node3709.members.0.js
2
2528029

3
2527997
species
node3710.members.0.js

13
2528009
species
node3711.members.0.js

1
2527968
species
node3712.members.0.js

1
2527994
species
node3713.members.0.js

2
2528010
species
node3714.members.0.js

2527980
6
node3715.members.0.js
species

species
node3716.members.0.js
3
2528017

node3717.members.0.js
species
1930277
1

species
node3718.members.0.js
50
2528023

2
2528030
species
node3719.members.0.js

3
2528025
species
node3720.members.0.js

class
666505
16

1546154
3
no rank

3
2528035
species
node3723.members.0.js

1
2483366
order

family
2483367
1

genus
1
2690160

node3727.members.0.js
species
1851148
1

666506
12
order

family
666507
12

666508
12
genus

species
12
547188

node3732.members.0.js
strain
1142394
12

phylum
134625
11

class
1921781
11

11
1921782
order

family
11
1921783

genus
1921784
11

11
1307763
species
node3738.members.0.js

phylum
3
204428

3
204429
class

51291
1
order

family
1
809

1
1113537
no rank

genus
1
810

83559
1
node3745.members.0.js
species

1963360
2
order

92714
2
family

genus
71666
2

species
71667
2

strain
node3750.members.0.js
2
716544

phylum
2
256845

class
1313211
2

278082
2
order

1674876
2
no rank

2
2094242
species
node3755.members.0.js

phylum
74201
75

21
203494
class

48461
21
order

2
1647988
family

genus
239934
2

node3761.members.0.js
species
239935
2

2
19
203557
family
node3762.members.0.js

genus
11
2735

2736
8
species

strain
node3765.members.0.js
8
240016

no rank
2625155
3

3
1882831
species
node3767.members.0.js

6
1348508
genus

no rank
2711230
6

6
2711231
species
node3770.members.0.js

6
417295
no rank

species
node3772.members.0.js
4
2488809

2
1637999
species
node3773.members.0.js

class
414999
48

order
415000
48

5
134623
48
node3776.members.0.js
family

278955
9
no rank

species
node3778.members.0.js
9
794903

1961799
9
genus

1838286
9
node3780.members.0.js
species

genus
2028344
2

2
1796921
species
node3782.members.0.js

178440
19
genus

10
2649488
no rank

node3785.members.0.js
species
1882749
10

9
107709
species

9
452637
strain
node3787.members.0.js

2576890
4
genus

node3789.members.0.js
species
2576891
4

phylum
4
508458

class
649775
4

order
4
649776

4
649777
family

genus
1
336260

1
336261
species

strain
node3796.members.0.js
1
580340

1
81411
genus

81412
1
species

node3799.members.0.js
strain
584708
1

genus
508459
2

1197717
2
node3801.members.0.js
species

40117
23
phylum

class
23
203693

order
23
189778

189779
23
family

genus
1234
21

42253
3
node3807.members.0.js
species

11
1325564
species
node3808.members.0.js

330214
7
node3809.members.0.js
species

2
28261
genus

species
2
28262

2
289376
strain
node3812.members.0.js

1
2498710
clade

phylum
1
67814

1
693071
class

1
693072
order

family
1
693073

genus
1
693074

species
693075
1

node3820.members.0.js
strain
511051
1

443
1783272
164495
node3821.members.0.js
clade

phylum
946
1297

class
node3823.members.0.js
946
188787
2

118964
868
order

family
5
332247

332248
5
genus

species
5
332249

strain
node3828.members.0.js
5
649638

family
863
183710

130
genus
node3830.members.0.js
863
1298

309887
21
species

21
709986
strain
node3832.members.0.js

12
1299
species
node3833.members.0.js

node3834.members.0.js
species
1211322
19

node3835.members.0.js
species
1182571
26

68909
5
species

5
319795
strain
node3837.members.0.js

124
980427
183
node3838.members.0.js
species

node3839.members.0.js
strain
1288484
59

1182568
4
node3840.members.0.js
species

node3841.members.0.js
species
1309411
3

species
11
502394

11
745776
strain
node3843.members.0.js

25
2623546
no rank

node3845.members.0.js
species
2080419
8

2489213
7
node3846.members.0.js
species

2652443
10
node3847.members.0.js
species

432329
4
species

4
937777
strain
node3849.members.0.js

11
1768108
species
node3850.members.0.js

species
55148
4

strain
node3852.members.0.js
4
693977

2202254
7
node3853.members.0.js
species

node3854.members.0.js
species
317577
391

7
310783
species

546414
7
node3856.members.0.js
strain

order
68933
76

1
node3858.members.0.js
family
188786
76

genus
34
65551

4
277
species

strain
node3861.members.0.js
4
504728

52022
30
species

30
526227
strain
node3863.members.0.js

270
36
node3864.members.0.js
genus
14

species
node3865.members.0.js
2
2483360

species
node3866.members.0.js
2
56956

4
274
species
node3867.members.0.js
3

1
762633
strain
node3868.members.0.js

species
56957
1

751945
1
node3870.members.0.js
strain

species
node3871.members.0.js
1
456163

12
37636
species

node3873.members.0.js
strain
743525
12

186191
2
genus

186192
2
species

strain
node3876.members.0.js
2
869210

3
208447
genus

species
187137
3

strain
node3879.members.0.js
3
670487

39
544448
phylum

class
39
31969

order
26
2085

family
26
2092

genus
1
2129

species
1
134821

1
38504
no rank
node3886.members.0.js

1
genus
node3887.members.0.js
25
2093

1
171284
species
node3888.members.0.js

species group
1
656088

species
node3890.members.0.js
1
2102

node3891.members.0.js
species
2123
1

species
171632
4

strain
node3893.members.0.js
4
1415773

538220
8
node3894.members.0.js
species

node3895.members.0.js
species
29562
1

1
2118
species

267748
1
node3897.members.0.js
strain

86660
2
node3898.members.0.js
species

2
2099
species
node3899.members.0.js

1
171281
species
node3900.members.0.js

2094
1
node3901.members.0.js
species

node3902.members.0.js
species
29559
1

186328
8
order

family
2131
8

1
genus
node3905.members.0.js
8
2132

216935
2
species

node3907.members.0.js
strain
1276246
2

216944
2
species

strain
node3909.members.0.js
2
1276257

node3910.members.0.js
species
216946
1

1
2637901
no rank

216964
1
node3912.members.0.js
species

species
node3913.members.0.js
1
216938

order
186329
5

family
5
2146

4
2147
genus

3
38986
species

strain
node3918.members.0.js
3
1318466

1
2148
species
node3919.members.0.js

33926
1
genus

species group
1
85620

species
1
229545

1
322098
strain
node3923.members.0.js

200795
42
node3924.members.0.js
phylum
1

2682225
6
class

order
6
2682226

2682227
6
family

2682228
6
genus

node3929.members.0.js
species
2601677
6

class
475962
4

order
4
475963

family
4
475964

233191
4
genus

species
4
133453

926550
4
node3935.members.0.js
strain

2
292625
class

2
292629
order

2
292628
family

genus
1
233189

1
167964
species

926569
1
node3941.members.0.js
strain

1
1649478
genus

1
913107
species
node3943.members.0.js

class
32061
14

order
14
32064

11
1508595
suborder

1508635
11
family

120961
11
node3948.members.0.js
genus
2

2609473
1
no rank

357808
1
node3950.members.0.js
species

8
120962
species

8
383372
strain
node3952.members.0.js

suborder
1508594
3

3
1106
family

genus
1107
3

species
node3956.members.0.js
3
1108

1382928
6
class

1382929
6
order

family
1382930
6

6
1988031
genus

6
1806508
species
node3961.members.0.js

class
189775
3

subclass
2
85000

order
2
85001

suborder
2
255728

family
85002
2

genus
2
2056

2
2057
species

479434
2
node3969.members.0.js
strain

order
189776
1

1
189777
family

499
1
genus

500
1
species

node3974.members.0.js
strain
309801
1

301297
6
class

genus
670486
6

species
4
552810

4
552811
strain
node3978.members.0.js

species
node3979.members.0.js
2
1839801

384
1798711
clade

1117
384
node3981.members.0.js
phylum
5

4
307596
class

order
307595
4

family
1890422
4

genus
33071
4

4
33072
species

251221
4
node3987.members.0.js
strain

1955042
1
order

1
1955043
family

genus
1188227
1

species
1
1188228

1188229
1
node3992.members.0.js
strain

order
84
1890424

42
1890426
family

genus
36
1129

5
33070
species

node3997.members.0.js
strain
1917166
5

31
2626047
no rank
node3998.members.0.js
5

1
29410
species
node3999.members.0.js

32051
3
node4000.members.0.js
species

node4001.members.0.js
species
1916956
6

4
585425
species
node4002.members.0.js

node4003.members.0.js
species
321327
2

species
node4004.members.0.js
2
316279

node4005.members.0.js
species
2511069
3

node4006.members.0.js
species
166314
3

321332
2
node4007.members.0.js
species

genus
6
167375

59930
2
species

292564
2
node4010.members.0.js
strain

2627006
4
no rank

1851505
4
node4012.members.0.js
species

family
7
1890431

genus
7
217161

1173032
7
species

1173020
7
node4016.members.0.js
strain

family
8
1213

genus
8
1218

8
1219
species

1
74546
strain
node4020.members.0.js

node4021.members.0.js
strain
167542
4

node4022.members.0.js
strain
74547
3

4
1890436
family

1152
4
genus

no rank
2593292
4

4
82654
species
node4026.members.0.js

family
1890429
5

155977
5
genus

species
5
155978

node4030.members.0.js
strain
329726
5

2303730
8
family

170610
8
genus

1209493
8
species

strain
node4034.members.0.js
8
1641165

10
1890438
family

genus
10
47251

2
node4037.members.0.js
species
1184
5

strain
node4038.members.0.js
3
1973484

2650499
5
no rank

1752064
4
node4040.members.0.js
species

1080068
1
node4041.members.0.js
species

8
1161
78
node4042.members.0.js
order

4
1219117
no rank

1940762
4
node4044.members.0.js
species

1182
17
family

17
1203
genus

2618749
17
no rank

1137095
17
node4048.members.0.js
species

25
1162
family

5
1177
22
node4050.members.0.js
genus

17
2593658
no rank
node4051.members.0.js
3

1
76335
species
node4052.members.0.js

node4053.members.0.js
species
28072
5

8
2005458
species
node4054.members.0.js

genus
1163
3

no rank
2619674
2

2490939
1
node4057.members.0.js
species

1647413
1
node4058.members.0.js
species

1165
1
species

1
272123
strain
node4060.members.0.js

family
20
2661849

20
1186
genus

species
938406
6

1973478
6
node4064.members.0.js
strain

2
1973486
species

node4066.members.0.js
strain
1973488
2

2619626
6
no rank

4
1337936
species
node4068.members.0.js

node4069.members.0.js
species
1954171
2

32054
6
species

strain
node4071.members.0.js
6
1170562

1
1892263
family

genus
1
1190

no rank
494603
1

node4075.members.0.js
species
2005456
1

3
1892259
family

159191
3
genus

3
70799
species
node4078.members.0.js

order
1890505
53

53
1890528
family

54298
53
genus

species
54299
53

strain
node4083.members.0.js
53
251229

order
52604
5

family
5
1890498

genus
node4086.members.0.js
5
102115
4

102116
1
species

node4088.members.0.js
strain
111780
1

no rank
1
1983111

718217
1
species

1
1228987
forma specialis
node4091.members.0.js

node4092.members.0.js
subclass
1301283
153
1

order
90
1150

family
35
1892252

54304
27
genus

1160
27
species

388467
27
node4097.members.0.js
strain

genus
2
1205

2
1206
species

2
203124
strain
node4100.members.0.js

44471
6
genus

2642155
6
no rank

node4103.members.0.js
species
1173027
6

44887
42
no rank

42
864702
species
node4105.members.0.js

13
1892254
family

genus
1158
7

species
7
482564

7
179408
strain
node4109.members.0.js

genus
1155738
6

2
species
node4111.members.0.js
6
1155739

1454205
3
node4112.members.0.js
strain

1
1458985
strain
node4113.members.0.js

1118
62
order

family
1890449
7

1125
7
genus

no rank
3
2643300

1967666
3
node4118.members.0.js
species

1
node4119.members.0.js
species
1126
2

449468
1
node4120.members.0.js
strain

2
513223
species

1638788
2
node4122.members.0.js
strain

family
45
1890464

genus
268175
22

no rank
22
2648896

node4126.members.0.js
species
2005460
22

22
102231
genus

22
2623012
no rank

22
1173026
species
node4129.members.0.js

669357
1
genus

1
669359
species

strain
node4132.members.0.js
1
113355

family
1890452
10

genus
10
102234

species
379064
10

node4136.members.0.js
strain
755178
10

602
node4137.members.0.js
phylum
1239
16868

class
243
909932

order
1843489
195

family
31977
195

9
genus
node4141.members.0.js
192
29465

9
39777
species
node4142.members.0.js

39778
3
node4143.members.0.js
species

node4144.members.0.js
species
29466
168
148

strain
node4145.members.0.js
20
1316254

3
248315
species
node4146.members.0.js

1
node4147.members.0.js
genus
39948
3

node4148.members.0.js
species
39950
2

order
909929
48

11
1843490
family

genus
2093783
5

5
1930071
species
node4152.members.0.js

genus
6
365348

species
3
365349

strain
node4155.members.0.js
3
1192197

2629460
3
no rank

node4157.members.0.js
species
484770
3

37
1843491
family

genus
970
23

species
69823
1

node4161.members.0.js
strain
546271
1

971
2
species

subspecies
114197
2

2
927704
strain
node4164.members.0.js

no rank
node4165.members.0.js
20
2637378
3

node4166.members.0.js
species
712528
2

713030
7
node4167.members.0.js
species

species
node4168.members.0.js
8
1884263

14
158846
genus

node4170.members.0.js
species
158847
1

13
437897
species
node4171.members.0.js

2
526524
class

order
526525
2

family
128827
2

no rank
1
544447

2109692
1
node4176.members.0.js
species

genus
1647
1

node4178.members.0.js
species
1648
1

node4179.members.0.js
class
91061
15152
84

12632
1385
order
node4180.members.0.js
180

36
node4181.members.0.js
family
186822
509

76632
9
genus

species
377615
9

node4184.members.0.js
strain
717605
9

456492
3
genus

node4186.members.0.js
species
2583377
3

node4187.members.0.js
genus
44249
327
40

160799
1
node4188.members.0.js
species

node4189.members.0.js
species
248903
3

node4190.members.0.js
species
59843
3

2
61624
5
node4191.members.0.js
species

strain
node4192.members.0.js
2
1116391

strain
node4193.members.0.js
1
997761

1
2044880
species group

species
483937
1

1073571
1
node4196.members.0.js
strain

1462996
5
node4197.members.0.js
species

1126833
1
node4198.members.0.js
species

species
node4199.members.0.js
2
189425

node4200.members.0.js
species
1338368
1

185978
106
node4201.members.0.js
no rank
3

9
1536772
species
node4202.members.0.js

species
node4203.members.0.js
5
2704463

1695218
6
node4204.members.0.js
species

node4205.members.0.js
species
1536771
4

5
2704462
species
node4206.members.0.js

species
node4207.members.0.js
2
1536773

2023772
37
node4208.members.0.js
species

node4209.members.0.js
species
2211212
1

2660554
18
node4210.members.0.js
species

1870819
2
node4211.members.0.js
species

species
node4212.members.0.js
4
1536774

1980674
2
node4213.members.0.js
species

species
node4214.members.0.js
3
1536775

3
481743
species
node4215.members.0.js

2565926
2
node4216.members.0.js
species

species
node4217.members.0.js
4
49283

node4218.members.0.js
species
1473112
4

2
1401
species
node4219.members.0.js

128574
4
node4220.members.0.js
species

2
528191
species
node4221.members.0.js

5
2707005
species
node4222.members.0.js

node4223.members.0.js
species
79263
3

2
169760
species
node4224.members.0.js

6
species
node4225.members.0.js
10
1406

349520
4
node4226.members.0.js
strain

node4227.members.0.js
species
414771
6

162209
103
node4228.members.0.js
species

species
node4229.members.0.js
2
189426

172713
3
node4230.members.0.js
species

species
node4231.members.0.js
2
1464

species
node4232.members.0.js
1
1619311

3
44250
species
node4233.members.0.js

365617
2
species

strain
node4235.members.0.js
2
1268072

159743
1
species

985665
1
node4237.members.0.js
strain

27
node4238.members.0.js
genus
55080
114

42
51101
species
node4239.members.0.js

1393
25
node4240.members.0.js
species
22

node4241.members.0.js
strain
1200792
1

node4242.members.0.js
strain
358681
2

species
node4243.members.0.js
5
1465

no rank
10
2684853

10
2496837
species
node4245.members.0.js

species
node4246.members.0.js
5
54913

genus
14
329857

species
node4248.members.0.js
10
2674991

4
2507935
species
node4249.members.0.js

no rank
85151
6

genus
6
55079

no rank
6
2642329

1450761
6
node4253.members.0.js
species

family
node4254.members.0.js
6134
90964
29

1279
6058
node4255.members.0.js
genus
1167

29379
37
node4256.members.0.js
species

species
node4257.members.0.js
931
1290
930

145391
1
node4258.members.0.js
subspecies

species
node4259.members.0.js
42
29385

node4260.members.0.js
species
1286
4

species
node4261.members.0.js
12
1288

1
46126
species
node4262.members.0.js

6
150056
species
node4263.members.0.js

3
46127
species
node4264.members.0.js

node4265.members.0.js
species
1292
1820

25
91994
no rank

25
2025492
species
node4267.members.0.js

186
29388
species
node4268.members.0.js
182

4
72758
subspecies
node4269.members.0.js

node4270.members.0.js
species
29382
165

28035
23
node4271.members.0.js
species

87
1280
species
node4272.members.0.js

species
node4273.members.0.js
8
29380

1
29378
species
node4274.members.0.js

64
node4275.members.0.js
species
45972
65

strain
node4276.members.0.js
1
1276282

1282
1427
node4277.members.0.js
species
1389

176280
9
node4278.members.0.js
strain

node4279.members.0.js
strain
1449752
29

1
308354
species
node4280.members.0.js

1283
45
node4281.members.0.js
species
43

node4282.members.0.js
strain
279808
2

2
1294
species
node4283.members.0.js

genus
3
227979

1461582
3
node4285.members.0.js
species

2005363
20
genus

1849491
20
node4287.members.0.js
species

1
69965
23
node4288.members.0.js
genus

species
node4289.members.0.js
7
69966

8
2619255
no rank

species
node4291.members.0.js
8
1898474

1855823
7
node4292.members.0.js
species

genus
45669
1

1
407035
species
node4294.members.0.js

family
9
186823

genus
29330
4

species
4
405212

subspecies
1388
4

strain
node4299.members.0.js
4
521098

genus
432330
2

1214604
1
node4301.members.0.js
species

1903704
1
node4302.members.0.js
species

1129704
3
node4303.members.0.js
genus

25
186820
family

3
13
1637
genus
node4305.members.0.js

7
1639
species
node4306.members.0.js

1640
1
node4307.members.0.js
species

node4308.members.0.js
species
1643
1

node4309.members.0.js
species
1641
1

2755
12
genus

12
2756
species
node4311.members.0.js

186817
5532
node4312.members.0.js
family
134

genus
13
84406

no rank
2620237
5

2
2419842
species
node4315.members.0.js

species
node4316.members.0.js
3
403957

2
163877
species
node4317.members.0.js

node4318.members.0.js
species
2017483
1

species
node4319.members.0.js
5
1482

genus
3
2685905

node4321.members.0.js
species
2502791
3

genus
2675233
1

1
152268
species
node4323.members.0.js

1906945
19
genus

node4325.members.0.js
species
1426
11

1295642
8
node4326.members.0.js
species

node4327.members.0.js
genus
1386
1301
107

2
33932
species
node4328.members.0.js

1
node4329.members.0.js
species
1478
9

8
1349754
strain
node4330.members.0.js

node4331.members.0.js
species
189381
8

185979
26
no rank

2
1581038
species
node4333.members.0.js

node4334.members.0.js
species
2660750
1

2567941
1
node4335.members.0.js
species

1
1565991
species
node4336.members.0.js

1
756828
species
node4337.members.0.js

node4338.members.0.js
species
1742359
2

species
node4339.members.0.js
2
486398

node4340.members.0.js
species
2587848
6

species
node4341.members.0.js
3
2014076

3
352858
species
node4342.members.0.js

node4343.members.0.js
species
666686
1

2682541
2
node4344.members.0.js
species

1705566
1
node4345.members.0.js
species

node4346.members.0.js
species
1467
1

node4347.members.0.js
species
1398
5

species group
653685
14

653388
1
species subgroup

1
260554
species
node4350.members.0.js

1
1423
species
node4351.members.0.js

5
species subgroup
node4352.members.0.js
12
1938374

492670
7
node4353.members.0.js
species

79883
9
node4354.members.0.js
species

node4355.members.0.js
species
1670641
2

species
node4356.members.0.js
6
1404

7
324767
species

strain
node4358.members.0.js
7
1367477

species
node4359.members.0.js
4
450367

node4360.members.0.js
species
561879
7

86661
1086
node4361.members.0.js
species group
61

species
node4362.members.0.js
3
1405

131
1396
1007
node4363.members.0.js
species

88
526969
strain
node4364.members.0.js

778
526973
strain
node4365.members.0.js

526986
4
node4366.members.0.js
strain

1
222523
strain
node4367.members.0.js

526983
5
node4368.members.0.js
strain

node4369.members.0.js
species
1428
7

species
node4370.members.0.js
3
580165

no rank
5
2750818

2217832
5
node4372.members.0.js
species

node4373.members.0.js
species
1408
3

species
node4374.members.0.js
5
1664069

genus
29331
1

species
1
1449

1
698758
strain
node4377.members.0.js

74385
5
genus

2625209
5
no rank

node4380.members.0.js
species
2663022
5

genus
2675229
5

1
264697
species
node4382.members.0.js

species
node4383.members.0.js
2
228899

species
node4384.members.0.js
2
421767

genus
2675231
14

node4386.members.0.js
species
129985
13

279826
1
node4387.members.0.js
species

1
175304
genus

species
node4389.members.0.js
1
1472767

genus
150247
4

node4391.members.0.js
species
198467
4

1
289201
genus

1
2628296
no rank

node4394.members.0.js
species
2692425
1

node4395.members.0.js
genus
400634
18
3

1
2636778
no rank

node4397.members.0.js
species
2070463
1

species
node4398.members.0.js
1
1145276

7
1421
species
node4399.members.0.js

6
2086577
species
node4400.members.0.js

node4401.members.0.js
genus
129337
76
40

129338
7
node4402.members.0.js
species

1505648
4
node4403.members.0.js
species group
1

3
1394
species
node4404.members.0.js

species
node4405.members.0.js
11
1422

13
no rank
node4406.members.0.js
14
2642459

391290
1
node4407.members.0.js
species

183
genus
node4408.members.0.js
3861
1329200

255247
3329
node4409.members.0.js
species

1221500
349
node4410.members.0.js
species

genus
351195
1

1
1230341
species
node4412.members.0.js

45667
2
genus

node4414.members.0.js
species
1570
2

1055323
6
genus

6
33936
species
node4416.members.0.js

genus
53
2675230

53
665099
species

53
1196031
strain
node4419.members.0.js

genus
3
2675232

node4421.members.0.js
species
1193713
3

2675234
10
genus

3
79885
species

node4424.members.0.js
strain
398511
3

4
86665
species
node4425.members.0.js

3
199441
species
node4426.members.0.js

no rank
539002
115

539742
68
no rank

4
68
33986
genus
node4429.members.0.js

11
2644629
no rank

9
360911
species
node4431.members.0.js

2051906
2
node4432.members.0.js
species

47
340146
species
node4433.members.0.js

332410
6
species

262543
6
node4435.members.0.js
strain

539738
47
no rank

node4437.members.0.js
genus
1378
47
5

node4438.members.0.js
species
1379
25

15
29391
species
node4439.members.0.js

84135
1
node4440.members.0.js
species

2624949
1
node4441.members.0.js
no rank

family
186821
4

663587
4
no rank

species
4
85683

439292
4
node4445.members.0.js
strain

family
node4446.members.0.js
98
186818
1

2
1649
genus

species
node4448.members.0.js
1
1650

1
2644826
no rank

species
node4450.members.0.js
1
1750719

648800
14
genus

node4452.members.0.js
species
76853
11

2637870
3
no rank

2048654
3
node4454.members.0.js
species

genus
1
1569

298596
1
node4456.members.0.js
species

genus
157226
5

1508404
5
node4458.members.0.js
species

genus
1
160795

node4460.members.0.js
species
51173
1

genus
651660
2

node4462.members.0.js
species
417367
2

genus
node4463.members.0.js
71
1372
19

1
1374
species
node4464.members.0.js

species
node4465.members.0.js
1
1038856

no rank
31
2662419

2213202
2
node4467.members.0.js
species

species
node4468.members.0.js
29
2058136

11
192421
species
node4469.members.0.js

node4470.members.0.js
species
414778
8

648802
1
genus

species
node4472.members.0.js
1
241244

26
186824
family

genus
22
2023

species
node4475.members.0.js
22
2026

2689589
4
genus

2490858
4
node4477.members.0.js
species

node4478.members.0.js
order
186826
2436
229

14
186827
family

14
1375
genus

species
node4481.members.0.js
9
51665

species
node4482.members.0.js
5
1377

family
39
81850

46254
2
genus

336988
2
species

node4486.members.0.js
strain
1045004
2

5
node4487.members.0.js
genus
1243
25

node4488.members.0.js
species
33968
1

species
node4489.members.0.js
6
33964

node4490.members.0.js
species
1245
8

1252
2
node4491.members.0.js
species

1
node4492.members.0.js
species
1244
2

1
1229756
strain
node4493.members.0.js

1
1246
species
node4494.members.0.js

46255
12
genus

node4496.members.0.js
species
155866
3

species
node4497.members.0.js
1
46256

1249
8
node4498.members.0.js
species

46
81852
family

1350
36
node4500.members.0.js
genus
26

160453
1
node4501.members.0.js
species

node4502.members.0.js
species
1352
1

node4503.members.0.js
species
1351
1

node4504.members.0.js
species
1353
2

44008
2
node4505.members.0.js
species

node4506.members.0.js
species
37734
3

3
51668
genus

species
node4508.members.0.js
1
290335

2
51669
species
node4509.members.0.js

7
2737
genus

5
519472
species
node4511.members.0.js

species
node4512.members.0.js
2
633807

family
148
33958

22
node4514.members.0.js
genus
1578
147

655183
4
species group

node4516.members.0.js
species
1582
4

82688
2
node4517.members.0.js
species

node4518.members.0.js
species
1599
9

6
1613
species
node4519.members.0.js

2
267363
species
node4520.members.0.js

11
28038
species
node4521.members.0.js

1
83683
species
node4522.members.0.js

species
293371
1

1423778
1
node4524.members.0.js
strain

1610
2
node4525.members.0.js
species

node4526.members.0.js
species
1847728
1

1618
5
node4527.members.0.js
species

species
node4528.members.0.js
1
1303590

1
304207
species
node4529.members.0.js

1590
5
node4530.members.0.js
species

node4531.members.0.js
species
1720083
1

species
node4532.members.0.js
3
1587

species
node4533.members.0.js
7
47770

23
109790
species
node4534.members.0.js

5
17
1598
species
node4535.members.0.js

12
491077
strain
node4536.members.0.js

node4537.members.0.js
species
147802
20

3
47715
species
node4538.members.0.js

genus
1253
1

187452
1
node4540.members.0.js
species

node4541.members.0.js
family
1300
1854
17

genus
node4542.members.0.js
1506
1301
840

9
45634
12
node4543.members.0.js
species

3
889201
strain
node4544.members.0.js

species
node4545.members.0.js
2
1311

25
257758
species

node4547.members.0.js
strain
1054460
25

1308
39
node4548.members.0.js
species

1
1340
species
node4549.members.0.js

2
2382163
species
node4550.members.0.js

species
node4551.members.0.js
1
1346

node4552.members.0.js
species
113107
8

33
1433513
species
node4553.members.0.js

2608887
96
node4554.members.0.js
no rank
11

4
1902136
species
node4555.members.0.js

species
node4556.members.0.js
3
2610896

species
node4557.members.0.js
2
1316411

species
node4558.members.0.js
2
1316408

species
node4559.members.0.js
3
1759399

712624
16
node4560.members.0.js
species

node4561.members.0.js
species
2598453
9

node4562.members.0.js
species
712633
34

2576376
12
node4563.members.0.js
species

1343
9
node4564.members.0.js
species

63
1313
species
node4565.members.0.js
59

node4566.members.0.js
strain
487214
4

species
node4567.members.0.js
53
1303
28

strain
node4568.members.0.js
2
927666

1077464
6
node4569.members.0.js
subspecies

1458253
9
node4570.members.0.js
subspecies

node4571.members.0.js
strain
655813
8

4
671232
species group

1328
4
node4573.members.0.js
species

1304
4
node4574.members.0.js
species

1
1341
species
node4575.members.0.js

1305
74
node4576.members.0.js
species
62

node4577.members.0.js
strain
388919
12

2
78535
species
node4578.members.0.js

node4579.members.0.js
species
1318
17
5

1114965
11
node4580.members.0.js
strain

strain
node4581.members.0.js
1
760570

node4582.members.0.js
species
1302
71

node4583.members.0.js
species
28037
149
135

node4584.members.0.js
strain
365659
14

7
genus
node4585.members.0.js
331
1357

150
species
node4586.members.0.js
314
1358

node4587.members.0.js
subspecies
1360
2

162
1359
subspecies
node4588.members.0.js
160

strain
node4589.members.0.js
2
1104322

node4590.members.0.js
species
1364
4
3

1
297352
strain
node4591.members.0.js

1366
6
node4592.members.0.js
species

family
186828
106

4
29393
genus

4
29394
species
node4595.members.0.js

5
1470540
genus

3
2621505
5
node4597.members.0.js
no rank

species
node4598.members.0.js
2
1903686

4
genus
node4599.members.0.js
97
2747

1
2748
species
node4600.members.0.js

40
147709
species

40
1266845
subspecies
node4602.members.0.js

42
node4603.members.0.js
no rank
257487
52

node4604.members.0.js
species
1564681
2

species
node4605.members.0.js
7
208596

2592355
1
node4606.members.0.js
species

class
1737404
87

order
1737405
86

family
1
1737406

species
1
45497

strain
node4611.members.0.js
1
1288971

4
family
node4612.members.0.js
85
1570339

1
1161127
genus

species
node4614.members.0.js
1
1852373

29
150022
genus

3
node4616.members.0.js
species
1260
29

15
525282
strain
node4617.members.0.js

strain
node4618.members.0.js
11
334413

genus
162289
18

node4620.members.0.js
species
54005
18

genus
node4621.members.0.js
33
165779
4

33034
25
species

strain
node4623.members.0.js
25
525919

1870984
4
node4624.members.0.js
species

1
1737407
no rank

genus
1582879
1

species
node4627.members.0.js
1
1852374

class
775
186801

68295
31
node4629.members.0.js
order
1

12
186814
family

genus
2
1754

node4632.members.0.js
species
2325
2

genus
1
140458

species
1
85874

1089553
1
node4635.members.0.js
strain

no rank
5
42857

genus
3
42837

3
42838
species

429009
3
node4639.members.0.js
strain

44260
2
genus

1525
1
node4641.members.0.js
species

species
node4642.members.0.js
1
55779

genus
249529
4

911092
4
species

subspecies
4
119072

strain
node4646.members.0.js
4
273068

543371
17
family

4
28895
genus
node4648.members.0.js
1

1
1517
species
node4649.members.0.js

47490
1
node4650.members.0.js
species

1
2622527
no rank

species
node4652.members.0.js
1
1550240

12
genus
node4653.members.0.js
13
44000

1
55205
species

1
632518
strain
node4655.members.0.js

family
543372
1

genus
252965
1

species
1
252966

697281
1
node4659.members.0.js
strain

6
53433
order

4
972
family

2330
4
genus

species
2331
4

node4664.members.0.js
strain
572479
4

no rank
387655
1

genus
1
1769008

1
1323375
species
node4667.members.0.js

family
53434
1

genus
1
42417

1
42422
species

strain
node4671.members.0.js
1
748449

21
186802
738
node4672.members.0.js
order

family
6
186806

2
33951
genus

species
2
33952

strain
node4676.members.0.js
2
931626

4
1730
genus

3
29322
species

633697
3
node4679.members.0.js
strain

1
53442
species
node4680.members.0.js

541000
97
family

1263
7
genus

1161942
4
node4683.members.0.js
species

1160721
2
node4684.members.0.js
species

species
1
1264

1
697329
strain
node4686.members.0.js

946234
2
genus

node4688.members.0.js
species
292800
2

2
1905344
genus

species
node4690.members.0.js
2
1550024

2
2305133
no rank

2
1572656
species
node4692.members.0.js

7
552397
no rank

node4694.members.0.js
species
29343
7

216851
77
genus

species
node4696.members.0.js
77
853

150
186803
family
node4697.members.0.js
19

genus
2719231
3

species
3
29370

1297793
3
node4700.members.0.js
strain

2316020
2
genus

species
33038
2

strain
node4703.members.0.js
2
411470

genus
1164882
4

617123
4
node4705.members.0.js
species

28050
15
genus

39485
15
species

515620
15
node4708.members.0.js
strain

572511
11
genus

1
33035
species
node4710.members.0.js

2648079
10
no rank

5
2479767
species
node4712.members.0.js

1912897
5
node4713.members.0.js
species

1
698776
genus

no rank
1
2643091

node4716.members.0.js
species
2497860
1

207244
3
genus

node4718.members.0.js
species
649756
3

genus
1843210
1

no rank
2642604
1

1
2696063
species
node4721.members.0.js

2569097
4
genus

4
39488
species
node4723.members.0.js

21
35
186928
no rank
node4724.members.0.js

3
712991
species
node4725.members.0.js

species
node4726.members.0.js
6
2109691

species
node4727.members.0.js
1
2594789

node4728.members.0.js
species
2109690
4

genus
31
841

166486
31
species

strain
node4731.members.0.js
31
536231

genus
1506553
21

17
89153
species

553973
17
node4734.members.0.js
strain

node4735.members.0.js
species
1871021
4

2
990719
family

990721
2
genus

626937
2
node4738.members.0.js
species

27
538999
no rank

family
543314
2

genus
2
86331

2
114527
species
node4742.members.0.js

genus
2717089
2

species
node4744.members.0.js
2
2086584

543347
7
family

genus
7
178898

node4747.members.0.js
species
178899
7

6
1918454
genus

6
2610894
no rank

node4750.members.0.js
species
2610895
6

539000
10
family

genus
73918
10

73919
5
species

strain
node4754.members.0.js
5
644966

5
2619059
no rank

2293838
5
node4756.members.0.js
species

family
543349
1

genus
2733
1

1
2734
species

strain
node4760.members.0.js
1
292459

3
39779
no rank

3
2109688
species
node4762.members.0.js

380
31979
family

genus
114627
2

208226
2
species

2
293826
strain
node4766.members.0.js

1
1649459
genus

154046
1
species

742737
1
node4769.members.0.js
strain

15
genus
node4770.members.0.js
376
1485

281
species
node4771.members.0.js
287
1491

36826
1
no rank

498214
1
node4773.members.0.js
strain

node4774.members.0.js
strain
1415774
5

1
no rank
node4775.members.0.js
10
2614128

species
node4776.members.0.js
4
2212991

node4777.members.0.js
species
1042156
4

node4778.members.0.js
species
2507159
1

species
node4779.members.0.js
4
46867

1493
2
species

573061
2
node4781.members.0.js
strain

node4782.members.0.js
species
1561
3

node4783.members.0.js
species
84022
3

node4784.members.0.js
species
36745
3
1

1335640
2
strain

node4786.members.0.js
no rank
931276
2

species
node4787.members.0.js
4
1534

1
1548
species
node4788.members.0.js

species
node4789.members.0.js
1
1216932

node4790.members.0.js
species
1502
4

1519
20
node4791.members.0.js
species

species
node4792.members.0.js
4
1520

1488
2
node4793.members.0.js
species

1513
9
node4794.members.0.js
species

1492
1
node4795.members.0.js
species

2
169679
species
node4796.members.0.js

1
1542
species

strain
node4798.members.0.js
1
386415

1
1848399
genus

1
2633479
no rank

2599308
1
node4801.members.0.js
species

family
node4802.members.0.js
13
216572
8

459786
5
genus

351091
5
species

693746
5
node4805.members.0.js
strain

family
19
186804

1870884
15
genus

13
15
1496
species
node4808.members.0.js

2
499174
strain
node4809.members.0.js

2
1849828
genus

1505
2
node4811.members.0.js
species

1
2743582
genus

species
node4813.members.0.js
1
89152

genus
1
44259

1
143361
species

1
546269
strain
node4816.members.0.js

68298
2
family

862
1
genus

863
1
species

370885
1
subspecies

335541
1
node4821.members.0.js
strain

genus
1
129001

species
1
86170

643648
1
node4824.members.0.js
strain

12
186807
family

genus
471826
1

1
471827
species

node4828.members.0.js
strain
477974
1

51196
5
genus

species
51197
5

645991
5
node4831.members.0.js
strain

2
79206
genus

885581
2
species

646529
2
node4834.members.0.js
strain

genus
36853
1

species
142877
1

node4837.members.0.js
strain
871968
1

1
56112
genus
node4838.members.0.js

genus
2
1562

2
59610
species

2
349161
strain
node4841.members.0.js

family
5
2304686

genus
236752
1

node4844.members.0.js
species
236753
1

genus
3
2304692

1
288965
species

1
720554
strain
node4847.members.0.js

2
1515
species
node4848.members.0.js

genus
2304693
1

1
84032
species
node4850.members.0.js

1676648
7
class

1676649
7
order

family
1676650
7

7
1676651
genus

node4855.members.0.js
species
1555112
7

424
node4856.members.0.js
phylum
201174
145768

class
52
1497346

52
588673
order

320583
52
family

191494
52
genus

species
191495
52

469383
52
node4862.members.0.js
strain

84998
85
class

33
1643822
order

2
node4865.members.0.js
family
1643826
33

genus
11
84111

node4867.members.0.js
species
84112
11
6

strain
node4868.members.0.js
5
479437

genus
7
644652

species
node4870.members.0.js
2
1335613

471189
5
species

node4872.members.0.js
strain
657308
5

1
79603
genus

1
79604
species
node4874.members.0.js

genus
84108
3

node4876.members.0.js
species
84110
3

genus
2005386
6

species
node4878.members.0.js
6
1870985

447020
1
genus

446660
1
species

1384484
1
node4881.members.0.js
strain

genus
2
84162

species
2
84163

469378
2
node4884.members.0.js
strain

order
52
84999

1643824
34
node4886.members.0.js
family
4

24
133925
genus

species
node4888.members.0.js
6
604330

no rank
2638792
18

15
712411
species
node4890.members.0.js

node4891.members.0.js
species
2109685
3

6
1935188
genus

6
1871022
species
node4893.members.0.js

18
84107
family

genus
102106
17

17
74426
species
node4896.members.0.js

84113
1
no rank

species
node4898.members.0.js
1
1531429

12129
class
node4899.members.0.js
145020
1760

85004
164
order

3
family
node4901.members.0.js
164
31953

genus
54
2701

species
node4903.members.0.js
54
2702
52

553190
2
node4904.members.0.js
strain

node4905.members.0.js
genus
1678
107
8

species
5
158787

node4907.members.0.js
strain
1150461
5

species
node4908.members.0.js
10
35760

species
node4909.members.0.js
3
78344

1684
9
node4910.members.0.js
species

18
species
node4911.members.0.js
32
1680

strain
node4912.members.0.js
14
367928

1685
4
node4913.members.0.js
species

2
species
node4914.members.0.js
4
28025

subspecies
2
302911

strain
node4916.members.0.js
2
1167629

species
node4917.members.0.js
11
1689

8
1694
species
node4918.members.0.js
5

1447715
2
node4919.members.0.js
strain

subspecies
1690
1

1410605
1
node4921.members.0.js
strain

species
node4922.members.0.js
1
1681

1
1687
species
node4923.members.0.js

species
node4924.members.0.js
8
216816

1
1686
3
node4925.members.0.js
species

1
630129
2
node4926.members.0.js
subspecies

1447716
1
node4927.members.0.js
strain

76
2495578
order

family
76
85033

76
2078948
genus

1891644
76
node4931.members.0.js
species

order
622452
27

27
83778
family

genus
33981
27

species
27
131568

strain
node4936.members.0.js
27
266940

order
2037
473

5
node4938.members.0.js
family
2049
473

genus
1522056
17

node4940.members.0.js
species
1282737
8

2623556
9
no rank

2496867
9
node4942.members.0.js
species

40
2529408
genus
node4943.members.0.js
2

species
node4944.members.0.js
7
52773

3
181487
species

node4946.members.0.js
strain
888050
3

species
node4947.members.0.js
28
1660

genus
2050
9

9
2051
species

strain
node4950.members.0.js
9
548479

2740557
9
genus

9
178339
species
node4952.members.0.js

1
76833
genus

no rank
2609299
1

1
2495645
species
node4955.members.0.js

genus
2
1069494

node4957.members.0.js
species
1661
1

species
node4958.members.0.js
1
312285

139
node4959.members.0.js
genus
1654
390

12
1656
species
node4960.members.0.js

node4961.members.0.js
no rank
2609248
140
10

node4962.members.0.js
species
1851395
15

species
706438
41

node4964.members.0.js
strain
706439
41

2
2057743
species
node4965.members.0.js

5
2079536
species
node4966.members.0.js

species
6
649739

649743
6
node4968.members.0.js
strain

2057798
2
node4969.members.0.js
species

10
2321394
species
node4970.members.0.js

species
node4971.members.0.js
29
712122

2081702
17
node4972.members.0.js
species

3
2560010
species
node4973.members.0.js

14
544580
species
node4974.members.0.js

2057800
4
node4975.members.0.js
species

9
1655
species
node4976.members.0.js

species
node4977.members.0.js
41
52771

1659
3
node4978.members.0.js
species

16
111015
species
node4979.members.0.js

node4980.members.0.js
species
1960083
5

node4981.members.0.js
species
1912795
1

1
1852377
species
node4982.members.0.js

node4983.members.0.js
species
52774
5

order
322
1643684

85031
322
family

7
322
53460
genus
node4986.members.0.js

287
53461
species

479431
287
node4988.members.0.js
strain

24
1090615
species
node4989.members.0.js

no rank
2618968
4

4
1902245
species
node4991.members.0.js

8
1643683
order

family
85032
8

genus
8
28048

species
8
28049

8
351607
strain
node4996.members.0.js

order
node4997.members.0.js
17565
85009
233

family
node4998.members.0.js
3872
85015
465

genus
203
116071

75385
203
node5000.members.0.js
species

86795
152
genus

642780
152
node5002.members.0.js
species

2263
1839
genus
node5003.members.0.js
320

species
138
450734

node5005.members.0.js
strain
1300347
138

128
449461
species
node5006.members.0.js

species
node5007.members.0.js
107
402297

1295
2615069
no rank
node5008.members.0.js
276

species
node5009.members.0.js
95
2712223

species
node5010.members.0.js
80
2662361

2045452
70
node5011.members.0.js
species

117
2483798
species
node5012.members.0.js

node5013.members.0.js
species
2558918
92

species
node5014.members.0.js
48
2575373

2589074
81
node5015.members.0.js
species

28
2500546
species
node5016.members.0.js

node5017.members.0.js
species
196162
136

node5018.members.0.js
species
2582905
90

node5019.members.0.js
species
110319
182

node5020.members.0.js
species
2518370
123

2518371
122
node5021.members.0.js
species

node5022.members.0.js
species
1804624
30

genus
node5023.members.0.js
510
2040
54

1736691
151
node5024.members.0.js
species

species
node5025.members.0.js
75
2041

219314
86
species

86
585531
strain
node5027.members.0.js

144
2633570
no rank
node5028.members.0.js
23

40
2079793
species
node5029.members.0.js

node5030.members.0.js
species
2107713
36

45
2662028
species
node5031.members.0.js

1
genus
node5032.members.0.js
124
53387

node5033.members.0.js
species
546874
71

52
546871
species
node5034.members.0.js

genus
61
2044

species
node5036.members.0.js
61
2045

genus
23
117156

23
117157
species
node5038.members.0.js

genus
182639
71

species
182640
71

strain
node5041.members.0.js
71
479435

family
node5042.members.0.js
13460
31957
91

1912216
12298
node5043.members.0.js
genus
293

species
node5044.members.0.js
330
33011

69
node5045.members.0.js
species
33010
97

strain
node5046.members.0.js
28
1170318

1747
11578
node5047.members.0.js
species

4
1743
262
node5048.members.0.js
genus

node5049.members.0.js
species
1744
187

species
node5050.members.0.js
35
556499

species
node5051.members.0.js
36
119981

genus
1911549
45

45
2662260
no rank

node5054.members.0.js
species
2662261
45

69
1278221
genus

node5056.members.0.js
species
675864
69

96
1912215
genus
node5057.members.0.js
24

species
node5058.members.0.js
6
2057246

1749
48
node5059.members.0.js
species

node5060.members.0.js
species
1748
18

17
326
72763
genus
node5061.members.0.js

1610493
43
node5062.members.0.js
species

node5063.members.0.js
species
2161816
43

node5064.members.0.js
species
399497
80

41
1332264
species
node5065.members.0.js

no rank
2635419
102

102
1909732
species
node5067.members.0.js

7
node5068.members.0.js
genus
29404
213

no rank
node5069.members.0.js
89
2619695
3

40
2672569
species
node5070.members.0.js

species
node5071.members.0.js
46
2596828

630515
28
node5072.members.0.js
species

species
29405
89

89
1032480
strain
node5074.members.0.js

203133
1
genus

no rank
1
2633994

species
node5077.members.0.js
1
1871034

59
1912217
genus

species
node5079.members.0.js
59
1750

order
85014
18

family
18
85034

genus
283810
18

species
18
283811

446470
18
node5084.members.0.js
strain

no rank
52018
6

1650658
2
node5086.members.0.js
species

species
node5087.members.0.js
4
2487353

1684
node5088.members.0.js
order
85007
73456

family
5
316606

genus
5
286801

species
5
286802

strain
node5092.members.0.js
5
640132

92
85025
47215
node5093.members.0.js
family

46285
1827
genus
node5094.members.0.js
34804

191292
2
node5095.members.0.js
species

species
132919
9

node5097.members.0.js
strain
101510
9

21
1829
species
node5098.members.0.js

15
18
43767
species
node5099.members.0.js

strain
node5100.members.0.js
3
685727

1792
5369
192944
no rank
node5101.members.0.js

species
node5102.members.0.js
4
1564114

46
1653479
species
node5103.members.0.js

species
node5104.members.0.js
79
2054902

node5105.members.0.js
species
2594007
3

species
node5106.members.0.js
1876
1045808

935199
30
node5107.members.0.js
species

node5108.members.0.js
species
1990687
2

1807790
42
node5109.members.0.js
species

3
1302308
species
node5110.members.0.js

17
2499145
species
node5111.members.0.js

species
node5112.members.0.js
2
2663121

node5113.members.0.js
species
2490853
349

node5114.members.0.js
species
1653478
811

node5115.members.0.js
species
1727214
27

1
679318
species
node5116.members.0.js

2507582
10
node5117.members.0.js
species

247
1723645
species
node5118.members.0.js

17
1805827
species
node5119.members.0.js

species
node5120.members.0.js
11
2567884

17
node5121.members.0.js
species
37919
23

strain
node5122.members.0.js
6
632772

species
node5123.members.0.js
14
1500843

5338
1833
5579
node5124.members.0.js
species

148
234621
strain
node5125.members.0.js

1136179
43
node5126.members.0.js
strain

node5127.members.0.js
strain
1289591
50

1830
71
node5128.members.0.js
species

species
node5129.members.0.js
8
38310

5
node5130.members.0.js
species
103816
9

node5131.members.0.js
strain
1435356
4

334542
315
node5132.members.0.js
species

species
1828
43

1051973
31
node5134.members.0.js
strain

1443893
12
node5135.members.0.js
strain

genus
node5136.members.0.js
838
1817
35

no rank
2637762
21

node5138.members.0.js
species
2382165
13

8
1047172
species
node5139.members.0.js

30
35
37329
species
node5140.members.0.js

5
247156
strain
node5141.members.0.js

species
node5142.members.0.js
7
37332

node5143.members.0.js
species
1823
13

257277
9
node5144.members.0.js
species

species
node5145.members.0.js
353
1824

25
455432
species
node5146.members.0.js

species
9
37330

strain
node5148.members.0.js
9
1415166

node5149.members.0.js
species
2213200
21

273
135487
279
node5150.members.0.js
species

strain
node5151.members.0.js
6
1127134

37326
31
node5152.members.0.js
species
25

node5153.members.0.js
strain
1133849
6

family
13087
85026

1663
node5155.members.0.js
genus
2053
13087

67
2054
species
node5156.members.0.js

species
node5157.members.0.js
512
2055

63
84595
species

63
1112204
strain
node5159.members.0.js

1004901
42
node5160.members.0.js
species

node5161.members.0.js
no rank
2657482
10583
520

10
2597659
species
node5162.members.0.js

species
node5163.members.0.js
234
337191

node5164.members.0.js
species
2698900
9721

2676309
39
node5165.members.0.js
species

21
2059875
species
node5166.members.0.js

38
1737359
species
node5167.members.0.js

158898
15
node5168.members.0.js
species

species
node5169.members.0.js
23
84096

68
1136941
species
node5170.members.0.js

species
node5171.members.0.js
19
2420509

32
36822
species
node5172.members.0.js

743
3067
1762
family
node5173.members.0.js

genus
node5174.members.0.js
628
1763
80

1552759
22
node5175.members.0.js
species

29
204
2642494
no rank
node5176.members.0.js

species
node5177.members.0.js
65
2487344

node5178.members.0.js
species
189918
3

25
1682113
species
node5179.members.0.js

species
node5180.members.0.js
19
1547487

2587868
5
node5181.members.0.js
species

2
2051552
species
node5182.members.0.js

node5183.members.0.js
species
1561223
2

node5184.members.0.js
species
1920667
7

19
212767
species
node5185.members.0.js

1545728
11
node5186.members.0.js
species

node5187.members.0.js
species
1936029
17

7
node5188.members.0.js
species group
2249310
70

node5189.members.0.js
species
1784
4

2
722731
species
node5190.members.0.js

470076
8
node5191.members.0.js
species

220927
17
node5192.members.0.js
species

species
node5193.members.0.js
15
53376

species
node5194.members.0.js
12
292462

species
node5195.members.0.js
5
185642

species
node5196.members.0.js
2
1789

3
39689
species
node5197.members.0.js

species
node5198.members.0.js
17
44010

1781
13
node5199.members.0.js
species

species
node5200.members.0.js
2
386911

2
10
77643
species group
node5201.members.0.js

node5202.members.0.js
species
78331
7
2

1205677
2
node5203.members.0.js
strain

node5204.members.0.js
strain
1205674
3

species
1773
1

strain
node5206.members.0.js
1
1097669

species
node5207.members.0.js
17
43348

29311
4
species

node5209.members.0.js
strain
1202450
4

459858
8
node5210.members.0.js
species

4
1769
species
node5211.members.0.js

node5212.members.0.js
species
482462
54

node5213.members.0.js
species group
120793
42
9

species
339268
3

3
1041522
strain
node5215.members.0.js

species
node5216.members.0.js
7
560555

1
species
node5217.members.0.js
4
1767

3
1203599
subspecies

3
1138871
strain
node5219.members.0.js

4
222805
species
node5220.members.0.js

node5221.members.0.js
species
701042
6

9
1764
species

3
1770
subspecies
node5223.members.0.js

3
439334
4
node5224.members.0.js
subspecies

node5225.members.0.js
strain
1229671
1

strain
node5226.members.0.js
2
243243

4
2094119
species
node5227.members.0.js

species
node5228.members.0.js
2
398694

species
node5229.members.0.js
7
1775

species
node5230.members.0.js
6
1768

169765
7
node5231.members.0.js
species

species
node5232.members.0.js
9
590652

species
node5233.members.0.js
41
1389713

247
1595
1866885
genus
node5234.members.0.js

13
1771
species
node5235.members.0.js

9
1799
species
node5236.members.0.js

306
319707
species
node5237.members.0.js

82
species
node5238.members.0.js
93
1804

278137
6
node5239.members.0.js
strain

5
350054
strain
node5240.members.0.js

11
444597
species
node5241.members.0.js

node5242.members.0.js
species
28047
10

node5243.members.0.js
species
1534348
11

node5244.members.0.js
species
39691
8

species
node5245.members.0.js
101
146017

species
node5246.members.0.js
26
212765

126673
43
node5247.members.0.js
species

node5248.members.0.js
species
1797
19

species
node5249.members.0.js
16
36813

node5250.members.0.js
species
1286180
26

39
1791
species
node5251.members.0.js

node5252.members.0.js
species
85693
2

node5253.members.0.js
species
319706
100

35
1286181
species
node5254.members.0.js

node5255.members.0.js
species
1534349
13

species
node5256.members.0.js
28
1772
26

node5257.members.0.js
strain
1214915
2

39694
2
node5258.members.0.js
species

species
36814
17

17
710685
strain
node5260.members.0.js

16
39695
species
node5261.members.0.js

node5262.members.0.js
species
53462
15

node5263.members.0.js
species
39688
18

758802
6
node5264.members.0.js
species

species
node5265.members.0.js
32
67081

species
node5266.members.0.js
21
134601

species
46351
19

node5268.members.0.js
strain
1122247
19

node5269.members.0.js
species
1431246
54

10
110539
species

10
350058
strain
node5271.members.0.js

7
1249101
species
node5272.members.0.js

species
9
1800

9
710421
strain
node5274.members.0.js

25
216929
species
node5275.members.0.js

39692
16
node5276.members.0.js
species

species
node5277.members.0.js
15
1793

1810
26
species

strain
node5279.members.0.js
26
1354275

25
1792
species
node5280.members.0.js

2
1794
species
node5281.members.0.js

77
1766
species
node5282.members.0.js

27
370526
species
node5283.members.0.js

35
1073531
genus

1118379
3
node5285.members.0.js
species

13
1788
species
node5286.members.0.js

node5287.members.0.js
species
29314
7

875328
12
node5288.members.0.js
species

697025
5
genus

5
639313
species

node5291.members.0.js
strain
443218
5

2126281
8
genus

node5293.members.0.js
species
1069220
8

4
53
670516
genus
node5294.members.0.js

species
node5295.members.0.js
20
36809
18

1962118
2
node5296.members.0.js
subspecies

4
1774
species
node5297.members.0.js

83262
16
node5298.members.0.js
species

species
node5299.members.0.js
9
404941

40
85028
family

node5301.members.0.js
genus
2060
40
5

node5302.members.0.js
species
57704
18

13
17
2061
species
node5303.members.0.js

node5304.members.0.js
strain
521096
4

5417
85029
family

37914
5417
node5306.members.0.js
genus
1456

234
139021
species
node5307.members.0.js

2617939
2635
node5308.members.0.js
no rank
148

2440
712270
species
node5309.members.0.js

species
node5310.members.0.js
47
912801

546160
1030
node5311.members.0.js
species

species
node5312.members.0.js
62
499555

family
node5313.members.0.js
2909
1653
25

genus
node5314.members.0.js
2884
1716
514

152794
9
species

9
196164
strain
node5316.members.0.js

species
node5317.members.0.js
22
38301

node5318.members.0.js
species
161899
125

43990
108
node5319.members.0.js
species

441500
14
node5320.members.0.js
species

7
1718
10
node5321.members.0.js
species

strain
node5322.members.0.js
3
340322

species
11
42817

strain
node5324.members.0.js
11
1348662

9
1072256
species
node5325.members.0.js

11
1705
species
node5326.members.0.js

4
1737425
species
node5327.members.0.js

node5328.members.0.js
species
43771
18
16

strain
node5329.members.0.js
2
1267754

node5330.members.0.js
species
1471400
4

30
43770
species
node5331.members.0.js

3
1697
species
node5332.members.0.js

28028
9
node5333.members.0.js
species

8
191610
species
node5334.members.0.js

31
39
38289
species
node5335.members.0.js

strain
node5336.members.0.js
8
306537

1719
2
node5337.members.0.js
species

3
1408191
species

node5339.members.0.js
strain
931089
3

15
1223514
species

node5341.members.0.js
strain
1223515
15

161896
6
node5342.members.0.js
species

1050174
8
node5343.members.0.js
species

species
node5344.members.0.js
12
156976

4
161895
species
node5345.members.0.js

38302
22
node5346.members.0.js
species

38305
8
species

node5348.members.0.js
strain
1224164
8

1230998
22
species

strain
node5350.members.0.js
22
1437875

node5351.members.0.js
species
2594913
510

2
89154
species

node5353.members.0.js
strain
1200352
2

203263
5
species

1431546
5
node5355.members.0.js
strain

species
38288
89

585529
89
node5357.members.0.js
strain

species
node5358.members.0.js
123
146827

16
187491
species
node5359.members.0.js

14
191493
species

14
1437874
strain
node5361.members.0.js

species
3
1032851

node5363.members.0.js
strain
863239
3

species
4
161879

node5365.members.0.js
strain
645127
4

3
2080757
species
node5366.members.0.js

node5367.members.0.js
species
1862358
1

2
1652495
species
node5368.members.0.js

species
4
575200

node5370.members.0.js
strain
1224163
4

species
1121358
18

node5372.members.0.js
strain
558173
18

1404244
5
species

5
1404245
strain
node5374.members.0.js

species
349751
7

node5376.members.0.js
strain
1224162
7

species
20
225326

1121362
20
node5378.members.0.js
strain

401472
190
node5379.members.0.js
species

11
2488819
species
node5380.members.0.js

160386
3
species

3
1285583
strain
node5382.members.0.js

6
65058
species
node5383.members.0.js

38
156978
species
node5384.members.0.js

species
258224
5

662755
5
node5386.members.0.js
strain

species
16
169292

548476
16
node5388.members.0.js
strain

11
1727
species

858619
11
node5390.members.0.js
strain

node5391.members.0.js
species
1725
508

136857
6
node5392.members.0.js
species

species
node5393.members.0.js
14
1717
13

strain
node5394.members.0.js
1
698971

species
node5395.members.0.js
3
2079234

species
1
1231000

1
1408189
strain
node5397.members.0.js

no rank
2624378
64

2675219
2
node5399.members.0.js
species

2675218
8
node5400.members.0.js
species

node5401.members.0.js
species
1487956
14

node5402.members.0.js
species
2079535
12

node5403.members.0.js
species
2080740
6

species
node5404.members.0.js
13
702967

species
node5405.members.0.js
4
2675216

node5406.members.0.js
species
2487892
5

132
43768
species
node5407.members.0.js

32
697024
no rank

17
741759
genus

no rank
17
2645312

17
2597660
species
node5411.members.0.js

genus
15
1847725

species
node5413.members.0.js
15
1528099

8
85012
312
node5414.members.0.js
order

node5415.members.0.js
family
2012
79
7

2019
17
genus

species
17
2020

node5418.members.0.js
strain
471852
17

2
55
1988
genus
node5419.members.0.js

2626254
29
no rank

node5421.members.0.js
species
2591108
29

24
1411117
species
node5422.members.0.js

127
2004
family

76
83681
genus

no rank
node5425.members.0.js
76
2593643
1

species
node5426.members.0.js
33
2656914

42
1909395
species
node5427.members.0.js

1
genus
node5428.members.0.js
51
2000

no rank
25
2632669

25
2202249
species
node5430.members.0.js

25
2001
species

25
479432
strain
node5432.members.0.js

family
node5433.members.0.js
98
83676
9

83677
13
genus

species
2021
13

strain
node5436.members.0.js
13
269800

genus
11
104204

11
2635841
no rank

2498135
11
node5439.members.0.js
species

2
node5440.members.0.js
genus
2013
65

species
17
53437

17
1205910
strain
node5442.members.0.js

43
2014
species
node5443.members.0.js

3
280236
species

strain
node5445.members.0.js
3
1235441

order
30
2039638

family
30
2162846

2039639
9
genus

1884916
6
node5449.members.0.js
species

node5450.members.0.js
species
1884634
1

node5451.members.0.js
species
1884915
2

622681
21
genus

4
573600
species
node5453.members.0.js

6
1884907
species
node5454.members.0.js

species
node5455.members.0.js
6
1884904

species
node5456.members.0.js
5
1884914

order
85008
696

node5458.members.0.js
family
28056
696
47

1873
374
node5459.members.0.js
genus
88

1
51
2617518
no rank
node5460.members.0.js

2039870
8
node5461.members.0.js
species

node5462.members.0.js
species
2675222
17

2583243
12
node5463.members.0.js
species

13
2201999
species
node5464.members.0.js

299152
17
node5465.members.0.js
species

291594
5
node5466.members.0.js
species

13
47872
species
node5467.members.0.js

node5468.members.0.js
species
47858
18

node5469.members.0.js
species
709883
24

47850
7
node5470.members.0.js
species

14
1881
species
node5471.members.0.js

species
node5472.members.0.js
9
47865

node5473.members.0.js
species
356851
7

node5474.members.0.js
species
1877
11

species
node5475.members.0.js
13
261654

12
299146
species
node5476.members.0.js

species
node5477.members.0.js
8
479978

species
node5478.members.0.js
17
307121

node5479.members.0.js
species
1914461
18

2
356852
species
node5480.members.0.js

285665
14
node5481.members.0.js
species

species
node5482.members.0.js
26
47857

20
genus
node5483.members.0.js
192
1865

species
11
1866

strain
node5485.members.0.js
11
512565

113562
20
node5486.members.0.js
species

species
22
196914

1246995
22
node5488.members.0.js
strain

species
19
1867

node5490.members.0.js
strain
457423
19

33
2626549
100
node5491.members.0.js
no rank

species
node5492.members.0.js
17
649831

50
946334
species
node5493.members.0.js

genus
84593
10

10
1003110
species

10
263358
strain
node5496.members.0.js

168694
14
genus

species
168695
4

node5499.members.0.js
strain
369723
4

10
168697
species

node5501.members.0.js
strain
391037
10

673534
59
genus

36
node5503.members.0.js
no rank
2631981
59

2024580
14
node5504.members.0.js
species

2108470
5
node5505.members.0.js
species

node5506.members.0.js
species
2071627
4

414714
25
order

family
414877
25

414878
25
genus

304895
25
species

479433
25
node5511.members.0.js
strain

order
85010
908

family
node5513.members.0.js
908
2070
71

7
node5514.members.0.js
genus
1851
65

species
1852
12

node5516.members.0.js
strain
471857
12

species
40989
9

node5518.members.0.js
strain
882082
9

species
9
40990

9
928724
strain
node5520.members.0.js

5
2634184
no rank

2528243
5
node5522.members.0.js
species

20
632569
species

882083
20
node5524.members.0.js
strain

3
40988
species

3
882081
strain
node5526.members.0.js

genus
30
43356

43357
30
species

node5529.members.0.js
strain
1449976
30

19
142577
genus

species
node5531.members.0.js
19
530584

1847
248
node5532.members.0.js
genus
8

node5533.members.0.js
species
2074
33

35
240495
species

strain
node5535.members.0.js
35
675635

47
2619320
172
node5536.members.0.js
no rank

1
1688404
species
node5537.members.0.js

62
445576
species
node5538.members.0.js

species
node5539.members.0.js
14
1641402

node5540.members.0.js
species
1690815
48

genus
17
2029

node5542.members.0.js
species
860235
17

18
40566
35
node5543.members.0.js
genus

9
17
42197
species
node5544.members.0.js

8
103721
subspecies
node5545.members.0.js

genus
26
1137960

211114
26
node5547.members.0.js
species

35
genus
node5548.members.0.js
230
1813

31
33910
species
node5549.members.0.js

208439
2
node5550.members.0.js
species

30
1814
species

30
1068978
strain
node5552.members.0.js

1804986
22
node5553.members.0.js
species

22
31958
species
node5554.members.0.js

1
2618356
61
node5555.members.0.js
no rank

24
2653857
species
node5556.members.0.js

node5557.members.0.js
species
1896961
18

node5558.members.0.js
species
1911175
18

129921
27
node5559.members.0.js
species

genus
57
2071

103733
30
node5561.members.0.js
species

species
103731
27

strain
node5563.members.0.js
27
1179773

genus
25
1835

species
node5565.members.0.js
9
2665642

species
16
1836

node5567.members.0.js
strain
405948
16

genus
17
674734

2636053
17
no rank

species
node5570.members.0.js
17
1653480

16
genus
node5571.members.0.js
35
65496

species
node5572.members.0.js
9
340345

1470176
9
node5573.members.0.js
species

no rank
1
2644606

node5575.members.0.js
species
1612551
1

165301
33
genus

1586287
33
node5577.members.0.js
species

1643682
245
order

52
245
85030
family
node5579.members.0.js

38501
84
genus

species
84
138336

1146883
84
node5582.members.0.js
strain

genus
1860
51

1861
51
species

node5585.members.0.js
strain
526225
51

genus
88138
58

477641
58
node5587.members.0.js
species

85013
127
node5588.members.0.js
order
2

116
74712
family

1854
104
node5590.members.0.js
genus
13

species
node5591.members.0.js
5
106370

2632575
32
no rank

node5593.members.0.js
species
298653
19

node5594.members.0.js
species
710111
13

species
node5595.members.0.js
31
298654

species
10
1859

10
326424
strain
node5597.members.0.js

node5598.members.0.js
species
2714109
13

genus
1434010
12

no rank
12
2629395

12
1907575
species
node5601.members.0.js

no rank
9
1920255

1882833
9
node5603.members.0.js
species

16
1643818
no rank

16
147067
genus

2006
16
species

node5607.members.0.js
strain
469371
16

order
2681
85011

2062
2681
node5609.members.0.js
family
90

65
2063
genus

2066
42
species

strain
node5612.members.0.js
42
452652

node5613.members.0.js
species
1894
3

no rank
20
2633591

20
2018025
species
node5615.members.0.js

16
228398
genus

species
node5617.members.0.js
16
2126346

722
2510
1883
genus
node5618.members.0.js

species
68202
12

12
1303692
strain
node5620.members.0.js

2
1642299
species
node5621.members.0.js

species
node5622.members.0.js
10
1077946

species
node5623.members.0.js
10
1960

node5624.members.0.js
species
1888
34

node5625.members.0.js
species
66425
11

species
6
33903

strain
node5627.members.0.js
6
227882

6
58346
species
node5628.members.0.js

1477431
8
species group

species
3
1902

3
100226
strain
node5631.members.0.js

1886
5
node5632.members.0.js
species

1916
2
node5633.members.0.js
species
1

1
457428
strain
node5634.members.0.js

2548456
9
node5635.members.0.js
species

285450
9
species

subspecies
149682
9

strain
node5638.members.0.js
9
1352936

node5639.members.0.js
species
68209
9

node5640.members.0.js
species
1977088
10

species group
629295
32

species subgroup
1482558
11

species
node5643.members.0.js
11
1908
6

5
1172567
strain
node5644.members.0.js

8
1482561
species subgroup

species
node5646.members.0.js
8
1892

1482596
13
species subgroup

species
13
1911

2
node5649.members.0.js
subspecies
67263
13

11
455632
strain
node5650.members.0.js

30
29303
species

node5652.members.0.js
strain
1003195
30

species
node5653.members.0.js
30
47763

node5654.members.0.js
species
362257
16

48665
2
node5655.members.0.js
species

species
1914
16

58340
16
node5657.members.0.js
subspecies

9
1969
22
node5658.members.0.js
species

strain
node5659.members.0.js
13
1079985

species
node5660.members.0.js
15
1932

42684
16
species

1214242
16
node5662.members.0.js
strain

1905
27
node5663.members.0.js
species

species
2
1169025

591167
2
node5665.members.0.js
strain

40318
6
node5666.members.0.js
species

25
1038928
species

1038929
25
node5668.members.0.js
strain

species
node5669.members.0.js
7
146923

2
67260
species
node5670.members.0.js

node5671.members.0.js
species
38300
14

species
68246
21

node5673.members.0.js
subspecies
284034
21

2049881
9
node5674.members.0.js
species

8
68223
species
node5675.members.0.js

node5676.members.0.js
species
47716
8

species
node5677.members.0.js
4
1901

13
67581
species

node5679.members.0.js
strain
665577
13

9
68280
species

strain
node5681.members.0.js
9
653045

10
1907
species
node5682.members.0.js

83656
26
node5683.members.0.js
species

3
1355015
species
node5684.members.0.js

1950
14
species

subspecies
55158
14

316280
14
node5687.members.0.js
strain

68570
15
node5688.members.0.js
species

1927
19
node5689.members.0.js
species

species
node5690.members.0.js
8
1690221

11
1967
species
node5691.members.0.js

3
1906
species

node5693.members.0.js
strain
1319510
3

node5694.members.0.js
species
408015
5

species
node5695.members.0.js
13
66871

species
node5696.members.0.js
18
68270

13
1783515
species
node5697.members.0.js

67345
9
node5698.members.0.js
species

node5699.members.0.js
species
1928
4

species
node5700.members.0.js
9
1437453

14
33899
species
node5701.members.0.js

species
node5702.members.0.js
9
1616117

species
1
1971

node5704.members.0.js
strain
316284
1

node5705.members.0.js
species
68203
8

species
node5706.members.0.js
13
36818

species
348043
10

strain
node5708.members.0.js
10
1214101

node5709.members.0.js
species
68175
3

68249
14
node5710.members.0.js
species

379067
14
species

strain
node5712.members.0.js
14
749414

species
node5713.members.0.js
4
132473

node5714.members.0.js
species
2496836
6

67267
18
node5715.members.0.js
species

species
node5716.members.0.js
11
1535768

node5717.members.0.js
species
67304
18

node5718.members.0.js
species
1885
13

7
node5719.members.0.js
species
1912
12

2
subspecies
node5720.members.0.js
4
311982

2
1133850
strain
node5721.members.0.js

264445
1
node5722.members.0.js
subspecies

132
no rank
node5723.members.0.js
679
2593676

species
node5724.members.0.js
29
1841249

6
1262452
species
node5725.members.0.js

species
node5726.members.0.js
2
1961713

node5727.members.0.js
species
1882757
12

species
node5728.members.0.js
3
1522758

8
2692233
species
node5729.members.0.js

7
2585716
species
node5730.members.0.js

species
node5731.members.0.js
8
2203210

node5732.members.0.js
species
2072505
11

10
1736046
species
node5733.members.0.js

species
node5734.members.0.js
10
2305220

species
node5735.members.0.js
2
355249

3
1661694
species
node5736.members.0.js

species
node5737.members.0.js
5
2563602

node5738.members.0.js
species
2305221
7

node5739.members.0.js
species
234612
8

node5740.members.0.js
species
1881022
2

10
1849967
species
node5741.members.0.js

species
node5742.members.0.js
8
2184053

species
node5743.members.0.js
3
2662065

1
2594455
species
node5744.members.0.js

species
node5745.members.0.js
5
2705439

species
node5746.members.0.js
15
2175864

species
node5747.members.0.js
6
206662

species
node5748.members.0.js
2
1436085

8
646637
species
node5749.members.0.js

1495638
1
node5750.members.0.js
species

465541
1
node5751.members.0.js
species

species
node5752.members.0.js
3
1265601

species
node5753.members.0.js
9
2059884

node5754.members.0.js
species
1561022
2

species
node5755.members.0.js
13
1649184

1
1914992
species
node5756.members.0.js

node5757.members.0.js
species
317660
1

species
node5758.members.0.js
4
1436084

1812480
15
node5759.members.0.js
species

species
node5760.members.0.js
21
2676871

1964449
6
node5761.members.0.js
species

species
node5762.members.0.js
10
2203205

node5763.members.0.js
species
2692235
1

species
node5764.members.0.js
1
2094021

species
node5765.members.0.js
5
862751

2684468
13
node5766.members.0.js
species

5
1851167
species
node5767.members.0.js

node5768.members.0.js
species
2135430
15

node5769.members.0.js
species
1972846
1

species
node5770.members.0.js
2
2202000

node5771.members.0.js
species
1837283
6

10
2686304
species
node5772.members.0.js

species
node5773.members.0.js
7
2083284

node5774.members.0.js
species
2662397
34

2653200
19
node5775.members.0.js
species

23
2695266
species
node5776.members.0.js

node5777.members.0.js
species
2005885
5

node5778.members.0.js
species
1938841
5

species
node5779.members.0.js
14
2136401

species
node5780.members.0.js
7
349971

node5781.members.0.js
species
1442032
6

species
node5782.members.0.js
3
1984801

node5783.members.0.js
species
1476754
1

2692234
11
node5784.members.0.js
species

2153485
4
node5785.members.0.js
species

2282738
11
node5786.members.0.js
species

species
node5787.members.0.js
16
1855352

1725411
7
node5788.members.0.js
species

node5789.members.0.js
species
2203204
8

444103
8
node5790.members.0.js
species

3
2306165
species
node5791.members.0.js

18
2582831
species
node5792.members.0.js

1935
10
node5793.members.0.js
species

188770
3
node5794.members.0.js
species

node5795.members.0.js
species
2174846
6

9
1827580
species
node5796.members.0.js

1889
7
node5797.members.0.js
species

species
node5798.members.0.js
19
73044

18
116188
species
node5799.members.0.js

species
1
68192

node5801.members.0.js
subspecies
477245
1

node5802.members.0.js
species
1890
10

species
1930
25

node5804.members.0.js
strain
680198
25

3
68214
species
node5805.members.0.js

285570
14
node5806.members.0.js
species

species
node5807.members.0.js
60
54571

species
node5808.members.0.js
5
2599401

node5809.members.0.js
species
28894
14

9
species
node5810.members.0.js
17
193462

1352941
8
node5811.members.0.js
strain

node5812.members.0.js
species
553510
9

1893
16
node5813.members.0.js
species

45398
23
node5814.members.0.js
species

67258
2
node5815.members.0.js
species

node5816.members.0.js
species
1915
12

species
node5817.members.0.js
6
1940

order
72
1217098

family
72
1217100

281472
72
node5820.members.0.js
genus
18

species
node5821.members.0.js
23
419479

no rank
31
2624397

31
1798224
species
node5823.members.0.js

35660
85006
order
node5824.members.0.js
2602

85016
230
node5825.members.0.js
family
2

genus
1926259
22

no rank
2649176
22

node5828.members.0.js
species
2654547
22

10
665568
genus

species
node5830.members.0.js
10
545619

1707
179
node5831.members.0.js
genus
20

1711
21
species

21
446466
strain
node5833.members.0.js

node5834.members.0.js
species
1708
10

node5835.members.0.js
no rank
2620175
97
6

19
2591145
species
node5836.members.0.js

node5837.members.0.js
species
2704467
30

species
node5838.members.0.js
15
2654191

2003551
27
node5839.members.0.js
species

2566013
22
node5840.members.0.js
species

9
11
species

node5842.members.0.js
strain
593907
9

162491
17
genus

no rank
17
2619021

17
1179673
species
node5845.members.0.js

133
85021
3173
node5846.members.0.js
family

node5847.members.0.js
genus
53457
2435
180

node5848.members.0.js
species
53458
299

node5849.members.0.js
species
262209
164

857417
1792
node5850.members.0.js
species

265976
188
node5851.members.0.js
genus
26

767452
33
node5852.members.0.js
species

no rank
2643101
21

2593973
21
node5854.members.0.js
species

1078471
58
node5855.members.0.js
species

node5856.members.0.js
species
1758689
29

species
247333
21

21
1123251
strain
node5858.members.0.js

367298
49
genus

species
node5860.members.0.js
49
443156

genus
267408
43

no rank
43
2663846

43
1658671
species
node5863.members.0.js

6
genus
node5864.members.0.js
234
125287

no rank
node5865.members.0.js
151
2615080
2

node5866.members.0.js
species
2283195
47

node5867.members.0.js
species
2508882
62

40
2594265
species
node5868.members.0.js

species
node5869.members.0.js
77
1288636

43
53357
genus

53358
43
node5871.members.0.js
species
42

1
710696
strain
node5872.members.0.js

genus
99479
48

no rank
2643346
48

node5875.members.0.js
species
1813880
48

no rank
19
577468

genus
754249
19

species
node5878.members.0.js
19
1133546

9677
85023
family
node5879.members.0.js
1485

genus
node5880.members.0.js
12
1705353
4

6
708131
species
node5881.members.0.js

no rank
2623385
2

species
node5883.members.0.js
2
1987356

12
1759331
genus

species
node5885.members.0.js
12
1619308

genus
6
1434032

node5887.members.0.js
species
1159327
6

genus
25
1433997

no rank
2643261
25

2683590
25
node5890.members.0.js
species

node5891.members.0.js
genus
69578
52
11

node5892.members.0.js
species
670052
14

no rank
node5893.members.0.js
17
2649013
4

species
node5894.members.0.js
13
1978566

species
node5895.members.0.js
10
2220095

genus
518733
73

73
412690
species
node5897.members.0.js

2680004
43
genus

node5899.members.0.js
species
2419774
43

genus
76634
12

no rank
12
2685235

2079792
12
node5902.members.0.js
species

20
genus
node5903.members.0.js
242
46352

node5904.members.0.js
species
684552
141

node5905.members.0.js
species
399736
47

no rank
2615065
34

34
2070347
species
node5907.members.0.js

447237
71
genus

2626248
71
no rank

species
node5910.members.0.js
32
1446794

39
1795630
species
node5911.members.0.js

110932
161
node5912.members.0.js
genus
15

82
1575
species
node5913.members.0.js
66

subspecies
31966
4

node5915.members.0.js
strain
1389489
4

subspecies
59736
12

node5917.members.0.js
strain
281090
12

37
64
2663824
no rank
node5918.members.0.js

species
node5919.members.0.js
27
1798223

43
genus
node5920.members.0.js
116
2034

2035
19
species

19
138532
no rank
node5922.members.0.js

node5923.members.0.js
species
69373
14

257496
40
node5924.members.0.js
no rank
7

2070337
12
node5925.members.0.js
species

species
node5926.members.0.js
11
1561023

species
node5927.members.0.js
10
1905847

genus
124
1573

species
node5929.members.0.js
124
28447
38

17
33013
20
node5930.members.0.js
subspecies

strain
node5931.members.0.js
3
443906

node5932.members.0.js
subspecies
1874630
12

subspecies
node5933.members.0.js
15
33014

subspecies
node5934.members.0.js
28
31964

31963
11
node5935.members.0.js
subspecies

2258
33882
6359
node5936.members.0.js
genus

species
node5937.members.0.js
96
1072463

node5938.members.0.js
species
2541726
36

1
300019
species
node5939.members.0.js

species
node5940.members.0.js
71
743009

node5941.members.0.js
species
273677
49

species
node5942.members.0.js
18
936337

84292
322
node5943.members.0.js
species

904291
138
node5944.members.0.js
species

370764
81
node5945.members.0.js
species

359
no rank
node5946.members.0.js
2197
2609290

species
node5947.members.0.js
95
2489212

2614638
84
node5948.members.0.js
species

2103230
52
node5949.members.0.js
species

species
node5950.members.0.js
44
912630

node5951.members.0.js
species
2268461
56

node5952.members.0.js
species
2567934
62

node5953.members.0.js
species
1714373
142

species
node5954.members.0.js
94
2483401

2070348
7
node5955.members.0.js
species

node5956.members.0.js
species
1795053
114

2048898
185
node5957.members.0.js
species

2014534
177
node5958.members.0.js
species

species
node5959.members.0.js
20
1916917

node5960.members.0.js
species
2603598
103

species
node5961.members.0.js
4
1696072

64
2606451
species
node5962.members.0.js

367477
110
node5963.members.0.js
species

314
1938334
species
node5964.members.0.js

2614639
50
node5965.members.0.js
species

1906742
61
node5966.members.0.js
species

node5967.members.0.js
species
104336
296

node5968.members.0.js
species
36805
122

node5969.members.0.js
species
162426
156

species
node5970.members.0.js
8
1526412

291
82380
species
node5971.members.0.js

node5972.members.0.js
species
199592
77

49
2509458
species
node5973.members.0.js

2033
93
species

node5975.members.0.js
strain
979556
93

69
node5976.members.0.js
genus
33886
263

110937
23
node5977.members.0.js
species
21

1328866
2
node5978.members.0.js
strain

6
59737
species
node5979.members.0.js

node5980.members.0.js
species
33887
17

node5981.members.0.js
species
145458
1

node5982.members.0.js
species
1671680
21

species
node5983.members.0.js
9
33888

no rank
node5984.members.0.js
117
2609250
22

2609258
3
node5985.members.0.js
species

2609254
4
node5986.members.0.js
species

node5987.members.0.js
species
2609252
39

15
2609253
species
node5988.members.0.js

node5989.members.0.js
species
2609255
16

node5990.members.0.js
species
2609257
18

64
190323
genus
node5991.members.0.js
30

node5992.members.0.js
species
150123
11

9
23
2624265
no rank
node5993.members.0.js

species
node5994.members.0.js
9
2480625

2583822
5
node5995.members.0.js
species

genus
1434018
22

no rank
22
2621488

node5998.members.0.js
species
2592654
22

genus
427753
26

no rank
26
2635918

2599293
19
node6001.members.0.js
species

7
2282656
species
node6002.members.0.js

2
genus
node6003.members.0.js
103
55968

41
2621730
no rank

node6005.members.0.js
species
1935379
41

1784719
60
node6006.members.0.js
species

6
1655488
no rank

clade
6
1655489

genus
1
529881

no rank
1
2617988

node6011.members.0.js
species
1855377
1

genus
5
529883

node6013.members.0.js
species
232537
1

species
node6014.members.0.js
4
535712

genus
16
337004

279828
16
node6016.members.0.js
species

1195526
26
genus

node6018.members.0.js
species
2419771
26

28
1230698
genus

674079
28
node6020.members.0.js
species

32
33877
307
node6021.members.0.js
genus

species
node6022.members.0.js
47
453304

24
2509455
species
node6023.members.0.js

2639701
140
node6024.members.0.js
no rank
8

2498704
54
node6025.members.0.js
species

25
2080742
species
node6026.members.0.js

node6027.members.0.js
species
2585717
14

39
2592652
species
node6028.members.0.js

589382
64
node6029.members.0.js
species

genus
23
235888

23
2632331
no rank

species
node6032.members.0.js
17
2603292

species
node6033.members.0.js
3
2508880

2079791
3
node6034.members.0.js
species

family
85019
850

1696
850
node6036.members.0.js
genus
112

1703
103
node6037.members.0.js
species

273384
46
node6038.members.0.js
species

518
2614124
no rank

518
2575923
species
node6040.members.0.js

48
629680
species
node6041.members.0.js

node6042.members.0.js
species
1136497
23

303
85020
family
node6043.members.0.js
3

75
36739
genus
node6044.members.0.js
14

species
node6045.members.0.js
2
36740

40
1630135
species
node6046.members.0.js

1667168
19
node6047.members.0.js
species

43668
210
node6048.members.0.js
genus
42

node6049.members.0.js
species
2017485
13

556288
18
node6050.members.0.js
species

species
22
43669

node6052.members.0.js
strain
446465
22

node6053.members.0.js
species
1331682
38

node6054.members.0.js
species
2017484
21

56
2623841
no rank
node6055.members.0.js
3

node6056.members.0.js
species
2571029
36

17
1903186
species
node6057.members.0.js

genus
15
472568

15
472569
species
node6059.members.0.js

387
node6060.members.0.js
family
1268
13339

11
genus
node6061.members.0.js
202
32207

37
172042
species
node6062.members.0.js

node6063.members.0.js
species
43675
111
79

node6064.members.0.js
strain
680646
32

43
2047
species
node6065.members.0.js
30

node6066.members.0.js
strain
762948
13

24
596707
genus

37927
24
node6068.members.0.js
species

2078575
9
genus

9
1618207
species
node6070.members.0.js

genus
49
169133

2632435
49
node6072.members.0.js
no rank

2
1742992
genus

species
43663
2

290340
2
node6075.members.0.js
strain

116
1691
57493
genus
node6076.members.0.js

1275
538
node6077.members.0.js
species

no rank
2649579
22

22
1702043
species
node6079.members.0.js

node6080.members.0.js
species
71999
869

species
node6081.members.0.js
20
388357

species
node6082.members.0.js
70
1049583

species
node6083.members.0.js
3
72000

446860
53
node6084.members.0.js
species

genus
370735
57

no rank
57
2626589

2058657
57
node6087.members.0.js
species

74
1742989
genus
node6088.members.0.js
9

5
1933880
species
node6089.members.0.js

species
node6090.members.0.js
14
162496

37929
10
node6091.members.0.js
species

species
34
256701

strain
node6093.members.0.js
34
861360

no rank
2
2627139

2
1155384
species
node6095.members.0.js

3789
genus
node6096.members.0.js
10416
1269

node6097.members.0.js
no rank
2620948
197
3

404583
35
node6098.members.0.js
species

species
node6099.members.0.js
73
1179670

species
node6100.members.0.js
20
161213

66
404582
species
node6101.members.0.js

6430
1270
species
node6102.members.0.js

genus
1160973
5

no rank
5
2634694

5
2170745
species
node6105.members.0.js

genus
18
57494

18
2629769
no rank

2600320
18
node6108.members.0.js
species

1742993
119
node6109.members.0.js
genus
10

species
85085
6

node6111.members.0.js
strain
452863
6

361575
50
species

930171
50
node6113.members.0.js
strain

10
33
2647000
no rank
node6114.members.0.js

2590775
5
node6115.members.0.js
species

2590785
6
node6116.members.0.js
species

2590774
4
node6117.members.0.js
species

node6118.members.0.js
species
2697569
8

10
728066
species
node6119.members.0.js

species
node6120.members.0.js
10
121292

genus
1868332
7

7
556325
species
node6122.members.0.js

1645
6
genus

node6124.members.0.js
species
1646
6

1663
273
node6125.members.0.js
genus
20

no rank
node6126.members.0.js
188
235627
27

2600159
9
node6127.members.0.js
species

node6128.members.0.js
species
1704044
14

11
290399
species
node6129.members.0.js

species
node6130.members.0.js
10
2578107

node6131.members.0.js
species
1357915
16

2020486
13
node6132.members.0.js
species

1849032
22
node6133.members.0.js
species

species
node6134.members.0.js
2
2184581

28
2565366
species
node6135.members.0.js

species
node6136.members.0.js
7
2575374

12
1806905
species
node6137.members.0.js

1494608
2
node6138.members.0.js
species

3
347213
species
node6139.members.0.js

1652545
10
node6140.members.0.js
species

2079227
2
node6141.members.0.js
species

13
656366
species
node6142.members.0.js

node6143.members.0.js
species
2211210
10

42
37928
species
node6144.members.0.js

1
125316
43
node6145.members.0.js
family

14
84756
genus

14
84757
species

strain
node6148.members.0.js
14
471853

947525
28
genus

28
2626367
no rank

species
node6151.members.0.js
28
2171623

family
node6152.members.0.js
4365
85017
69

2820
genus
node6153.members.0.js
4196
157920

no rank
2624466
23

1980001
23
node6155.members.0.js
species

species
node6156.members.0.js
1353
1710

genus
33
186188

12
2509459
species
node6158.members.0.js

21
186189
species

446471
21
node6160.members.0.js
strain

genus
52
254250

22
139208
species

22
743718
strain
node6163.members.0.js

372663
30
species

30
1300344
strain
node6165.members.0.js

genus
15
289244

no rank
2626399
15

species
node6168.members.0.js
15
2509457

family
911
145357

genus
57495
26

species
node6171.members.0.js
26
1274

43
745364
genus

node6173.members.0.js
species
571913
43

genus
842
57499

species
1276
842

842
478801
strain
node6176.members.0.js

family
85018
12

genus
1184606
7

100225
7
node6179.members.0.js
species

genus
5
1862

node6181.members.0.js
species
1863
5

2
1331736
60
node6182.members.0.js
family

1331737
35
genus

35
2624404
no rank

2675754
35
node6185.members.0.js
species

23
626119
genus

23
2648503
no rank

species
node6188.members.0.js
23
2603206

family
145360
18

18
60919
genus

18
60920
species

446469
18
node6192.members.0.js
strain

family
58
145358

58
154116
genus

58
2626815
no rank

species
node6196.members.0.js
23
2589797

2483799
20
node6197.members.0.js
species

node6198.members.0.js
species
2585135
15

622450
4
order

4
622451
family

1849
4
genus

species
node6202.members.0.js
4
414996

class
84992
68

68
84993
order
node6204.members.0.js
2

633392
33
family

no rank
2562118
33

33
2664374
species
node6207.members.0.js

family
5
84994

genus
5
53634

species
53635
5

strain
node6211.members.0.js
5
525909

family
2448023
28

genus
28
682522

species
28
467094

node6215.members.0.js
strain
1313172
28

39
908620
class
node6216.members.0.js
1

908621
11
order

family
11
908622

genus
908623
11

no rank
11
2649358

species
node6221.members.0.js
11
1608957

order
8
1747768

8
1747769
family

genus
1747770
8

1670831
8
node6225.members.0.js
species

19
1755823
order

1755824
19
family

1755825
19
genus

node6229.members.0.js
species
1670830
19

1
1752188
no rank

node6231.members.0.js
species
1848754
1

class
79
84995

order
79
84996

34
84997
family

34
42255
genus
node6235.members.0.js
2

5
2051957
species
node6236.members.0.js

7
42256
species
node6237.members.0.js

17
20
49319
species
node6238.members.0.js

266117
3
node6239.members.0.js
strain

family
45
2600303

genus
2600304
45

45
496014
species
node6242.members.0.js

phylum
5
67819

1663419
4
class

4
1663425
order

4
1663426
family

4
1005038
genus

1005039
4
species

strain
node6249.members.0.js
4
661478

class
1077257
1

1077263
1
order

1
1077264
family

genus
1077265
1

454171
1
species

strain
node6255.members.0.js
1
1303518

83
57723
phylum

56
204432
class

order
14
332160

family
14
332161

14
332162
genus

14
332163
species

node6262.members.0.js
isolate
234267
14

204433
42
order

204434
42
family

genus
658061
13

species
13
658062

node6267.members.0.js
isolate
204669
13

genus
388463
3

2637509
3
no rank

3
2703788
species
node6270.members.0.js

genus
2608728
1

species
node6272.members.0.js
1
2211140

genus
33973
2

species
2
33075

240015
2
node6275.members.0.js
strain

392733
10
genus

species
870903
1

node6278.members.0.js
strain
401053
1

species
node6279.members.0.js
4
1592106

5
392734
species

5
926566
strain
node6281.members.0.js

940557
13
genus

no rank
2621151
6

species
node6284.members.0.js
6
2602070

1
940614
species

node6286.members.0.js
strain
682795
1

940615
6
species

6
1198114
strain
node6288.members.0.js

1813735
25
class

2211325
25
family

genus
25
2004797

species
node6292.members.0.js
25
1855912

class
1562566
2

2
458032
genus

2
458033
species

node6296.members.0.js
strain
981222
2

4
200940
phylum

4
67799
class

4
188710
order

family
188711
4

genus
241192
4

171695
4
species

strain
node6303.members.0.js
4
667014

no rank
2323
32

1783234
32
clade

95901
3
clade

no rank
1104668
3

2066483
3
node6308.members.0.js
species

1
67810
phylum

genus
1
2250122

2026885
1
node6311.members.0.js
species

node6312.members.0.js
phylum
95818
27
10

node6313.members.0.js
no rank
1895827
15
13

2
2572088
species
node6314.members.0.js

genus
2
1331051

2
1332188
species
node6316.members.0.js

clade
1
1783273

363464
1
phylum

no rank
1
1046947

node6320.members.0.js
species
1540872
1

200918
19
phylum

class
19
188708

order
2
1643947

family
1643949
2

160798
2
node6325.members.0.js
genus

2419
17
order

1643950
11
family

genus
2422
2

node6329.members.0.js
species
93466
2

genus
9
2420

46541
5
node6331.members.0.js
species

2421
4
species

node6333.members.0.js
strain
484019
4

family
188709
6

genus
6
1643951

57487
2
species

node6337.members.0.js
strain
1123384
2

species
119394
4

strain
node6339.members.0.js
4
688269

superkingdom
2157
304

1935183
1
clade

phylum
1
1655434

1
2732160
genus

2594042
1
node6344.members.0.js
species

node6345.members.0.js
clade
1783275
76
3

651137
61
phylum

2
1643678
class

1033996
2
order

2
1033997
family

genus
2
497726

2
497727
species

node6352.members.0.js
isolate
1237085
2

31932
2
order

338190
2
family

genus
node6355.members.0.js
2
338191
1

1
1510466
species

node6357.members.0.js
strain
1229908
1

57
651142
no rank

53
1825023
genus
node6359.members.0.js
1

46
1603555
species
node6360.members.0.js

6
1846278
species
node6361.members.0.js

genus
1078904
4

species
node6363.members.0.js
4
1903276

phylum
28889
12

183924
12
class

2
2266
order

family
2267
2

genus
1
2270

2271
1
species

768679
1
node6370.members.0.js
strain

genus
2276
1

species
1
70771

node6373.members.0.js
strain
444157
1

order
2281
10

10
118883
family

12914
1
genus

species
1
312539

1
619593
strain
node6378.members.0.js

genus
2
69655

111955
2
species

strain
node6381.members.0.js
2
273063

2
41674
genus

species
node6383.members.0.js
2
41675

genus
2
41980

79601
2
species

node6386.members.0.js
strain
1293036
2

2284
3
genus

no rank
3
2641160

species
node6389.members.0.js
3
1891280

227
28890
phylum

1
183988
class

68985
1
order

183713
1
family

2319
1
genus

1
2684913
no rank
node6395.members.0.js

clade
2283796
4

4
183967
class

order
4
1235850

family
4
1577788

4
1712382
no rank

4
1535962
species
node6401.members.0.js

class
183968
11

11
2258
order

1
node6404.members.0.js
family
2259
11

2263
7
genus

2627626
5
no rank

species
node6407.members.0.js
1
2598455

4
122420
species
node6408.members.0.js

node6409.members.0.js
species
71997
2

2260
2
genus

node6411.members.0.js
species
2261
1

1
53953
species

1
70601
strain
node6413.members.0.js

genus
1
83867

species
1
971279

strain
node6416.members.0.js
1
1343739

13
2283794
clade

class
183925
6

order
2158
6

6
2159
family

2160
3
genus

node6422.members.0.js
species
118062
1

2
868131
species
node6423.members.0.js

2
2172
genus

2
2638681
no rank

2
1609968
species
node6426.members.0.js

genus
145260
1

species
1
145262

187420
1
node6429.members.0.js
strain

class
183939
7

2182
7
order

family
2183
2

genus
2184
2

39152
2
species

strain
node6435.members.0.js
2
426368

family
5
196117

genus
5
196118

species
2
67760

node6439.members.0.js
strain
573063
2

3
83171
species

node6441.members.0.js
strain
573064
3

class
1
183980

order
2231
1

family
1
2232

genus
1
54260

species
1
54261

node6447.members.0.js
strain
589924
1

2290931
197
clade

183963
174
node6449.members.0.js
class
5

55
2235
order

22
2236
family

genus
1075398
2

2
2648404
no rank

node6454.members.0.js
species
2599399
2

genus
5
332246

5
413810
species

795797
5
node6457.members.0.js
strain

genus
1
1656823

1
1604004
species
node6459.members.0.js

3
1843185
genus

3
2583824
species
node6461.members.0.js

1070314
3
genus

no rank
2643768
3

2
2650975
species
node6464.members.0.js

1
2505977
species
node6465.members.0.js

2
1980514
genus

node6467.members.0.js
species
1873524
2

genus
6
2239

2
1407499
species
node6469.members.0.js

node6470.members.0.js
species
2242
3

1
2668073
no rank

species
node6472.members.0.js
1
751944

31
1963268
family

9
1542963
genus

1457250
9
node6475.members.0.js
species

2237
3
genus

2238
1
species

272569
1
node6478.members.0.js
strain

2
1932004
species
node6479.members.0.js

genus
3
146825

146826
1
species

node6482.members.0.js
strain
519442
1

430914
2
species

2
1033806
strain
node6484.members.0.js

203135
10
node6485.members.0.js
genus
1

species
node6486.members.0.js
2
57705

no rank
2610901
7

node6488.members.0.js
species
2610902
7

1073987
3
genus

3
2648975
no rank

node6491.members.0.js
species
1932360
3

3
63743
genus

species
416273
1

node6494.members.0.js
strain
268739
1

2
2257
species

2
348780
strain
node6496.members.0.js

no rank
2
38063

2
1071085
species
node6498.members.0.js

1644055
76
order

2116545
3
no rank

2678343
3
genus

species
node6502.members.0.js
3
2055893

1
33
1644056
family
node6503.members.0.js

genus
376170
16

6
9
2617521
no rank
node6505.members.0.js

node6506.members.0.js
species
1547898
1

2
1547899
species
node6507.members.0.js

7
1816183
species
node6508.members.0.js

genus
node6509.members.0.js
10
2251
6

node6510.members.0.js
species
114529
2

node6511.members.0.js
species
35746
1

2246
1
species

1
309800
strain
node6513.members.0.js

genus
6
1073986

node6515.members.0.js
species
699433
6

no rank
2678336
3

species
node6517.members.0.js
3
756883

family
1963271
37

genus
3
1450140

1073996
3
node6520.members.0.js
species

node6521.members.0.js
genus
56688
29
1

no rank
11
2642239

node6523.members.0.js
species
2497325
7

species
node6524.members.0.js
4
634157

node6525.members.0.js
species
337243
9

species
node6526.members.0.js
4
29284

2247
4
species

4
416348
strain
node6528.members.0.js

1644057
5
genus

5
755307
species
node6530.members.0.js

order
1644060
38

2
family
node6532.members.0.js
38
1644061

121871
4
genus

node6534.members.0.js
species
588898
2

species
2
62320

2
543526
strain
node6536.members.0.js

genus
332951
6

species
6
387341

strain
node6539.members.0.js
6
797302

genus
1
253106

node6541.members.0.js
species
2044521
1

genus
203193
2

species
229731
2

2
358396
strain
node6544.members.0.js

genus
3
387342

species
387343
3

node6547.members.0.js
strain
797210
3

genus
63742
1

species
13769
1

node6550.members.0.js
strain
547559
1

2
29287
genus

species
29288
2

node6553.members.0.js
strain
694430
2

genus
8
353799

353800
8
species

797299
8
node6556.members.0.js
strain

genus
9
88723

species
1
69525

797303
1
node6559.members.0.js
strain

1
88724
species
node6560.members.0.js

no rank
2622230
7

species
node6562.members.0.js
7
406552

224756
23
class

order
7
94695

2206
7
family

genus
2
196136

39669
2
species

2
679901
strain
node6568.members.0.js

2207
2
genus

node6570.members.0.js
species
2210
1

species
1
38027

1434118
1
node6572.members.0.js
strain

genus
3
2175

39664
3
node6574.members.0.js
species

14
2191
order

9
2194
family

genus
45989
9

species
9
2198

strain
node6579.members.0.js
9
368407

1198451
5
family

499551
2
genus

2
263906
species

node6583.members.0.js
strain
882090
2

genus
395331
3

species
3
358766

3
456442
strain
node6586.members.0.js

order
2
570264

2
570265
family

570266
2
genus

species
2
570267

304371
2
node6591.members.0.js
strain

489
10239
superkingdom

clade
2732004
6

kingdom
6
2732005

1
2732008
phylum

2732529
1
class

1
2732559
order

10508
1
family

10509
1
genus

species
1
1146873

serotype
node6601.members.0.js
1
931972

2732007
5
phylum

class
2732525
2

2
2732527
order

2
10240
family

10241
2
subfamily

genus
1
10282

species
1
99000

no rank
node6609.members.0.js
1
132475

genus
10257
1

species
node6611.members.0.js
1
129727

2732523
3
class

2732524
3
order

10501
3
family

3
181086
genus

no rank
358403
3

species
node6617.members.0.js
3
251749

clade
2559587
107

106
2732396
kingdom

2497569
2
phylum

subphylum
2
2497571

2
2497576
class

order
2
1980410

2
1980415
family

genus
2
1980517

1980521
2
node6626.members.0.js
species

103
2732408
phylum

class
2732506
103

76804
1
order

1
2499399
suborder

11118
1
family

subfamily
1
2501931

genus
694013
1

subgenus
2509487
1

species
node6635.members.0.js
1
694014

order
464095
102

102
699189
family

no rank
102
1111709

1911104
102
node6639.members.0.js
species

1
2732407
phylum

2732499
1
class

2732503
1
order

family
2732892
1

186768
1
genus

no rank
1
343865

1
274584
species
node6646.members.0.js

2732397
1
kingdom

1
2732409
phylum

class
2732514
1

order
1
2169561

family
186534
1

genus
10639
1

no rank
515319
1

species
node6654.members.0.js
1
515444

1
10442
family

558016
1
genus

10455
1
node6657.members.0.js
species

4
12429
no rank

4
2204151
no rank

4
51368
no rank

genus
2060084
4

3
2107708
species
node6662.members.0.js

1
2107707
species
node6663.members.0.js

2
2731342
clade

kingdom
2
2732092

phylum
2
2732415

2732421
2
class

order
2
2732533

151340
2
family

2169595
2
subfamily

333922
2
genus

2
337051
species

node6673.members.0.js
serotype
333923
2

family
1
196937

249585
1
genus

node6676.members.0.js
species
342409
1

clade
368
2731341

368
2731360
clade

366
2731618
phylum

class
2731619
366

366
28883
order

10662
7
family

subfamily
4
857479

genus
2732974
4

node6685.members.0.js
species
393598
4

2
1921525
genus
node6686.members.0.js

subfamily
1636616
1

genus
1636617
1

1190451
1
node6689.members.0.js
species

family
1
2560065

subfamily
1
2560070

node6692.members.0.js
genus
1917990
1

family
2731643
5

1
2731650
subfamily

genus
542837
1

no rank
979725
1

node6697.members.0.js
species
1141139
1

4
2731966
genus

species
4
2733944

4
1283079
no rank
node6700.members.0.js

family
10744
3

196895
1
no rank

species
node6703.members.0.js
1
469660

2169641
2
genus

no rank
2529391
2

species
node6706.members.0.js
2
490912

4
10699
350
node6707.members.0.js
family

genus
2560096
2

2
2560484
species

node6710.members.0.js
no rank
1887641
2

no rank
node6711.members.0.js
11
196894
1

node6712.members.0.js
species
1821559
2

node6713.members.0.js
species
416419
1

species
node6714.members.0.js
2
1566990

1327959
5
node6715.members.0.js
species

1623278
1
node6716.members.0.js
genus

subfamily
1
2169599

2169622
1
node6718.members.0.js
genus

16
23
1623305
genus
node6719.members.0.js

7
2050979
no rank

node6721.members.0.js
species
1262535
1

1262537
6
node6722.members.0.js
species

node6723.members.0.js
subfamily
2169601
8
6

1
2169635
genus

2169730
1
species

1
1821549
no rank
node6726.members.0.js

2169636
1
genus

species
1
2170042

1887656
1
node6729.members.0.js
no rank

186764
9
node6730.members.0.js
genus
1

species
node6731.members.0.js
5
1032892

species
node6732.members.0.js
3
1097754

2560192
2
genus

2560506
2
species

no rank
node6735.members.0.js
2
1887651

genus
2733211
1

species
1
2734276

no rank
node6738.members.0.js
1
1821561

node6739.members.0.js
genus
1982251
271
224

species
1982269
8

376758
8
node6741.members.0.js
no rank

1982258
3
species

no rank
node6743.members.0.js
3
1655016

1
1982277
species

node6745.members.0.js
no rank
1235647
1

1982304
1
species

node6747.members.0.js
no rank
1655019
1

species
1
1982283

1500814
1
node6749.members.0.js
no rank

1982271
2
species

no rank
node6751.members.0.js
2
504501

species
2
1982268

2
1229787
no rank
node6753.members.0.js

species
1
1982284

1235653
1
node6755.members.0.js
no rank

species
1
1982306

1
1655021
no rank
node6757.members.0.js

1
1982291
species

node6759.members.0.js
no rank
1500820
1

species
4
1982282

no rank
node6761.members.0.js
4
1500813

3
1982273
species

3
1500798
no rank
node6763.members.0.js

14
2079398
no rank

node6765.members.0.js
species
1747271
6

2
1654780
species
node6766.members.0.js

node6767.members.0.js
species
1690805
6

species
1
1982292

1500821
1
node6769.members.0.js
no rank

species
1982254
4

1655012
4
node6771.members.0.js
no rank

subfamily
1
1982876

genus
1982898
1

species
1
1983119

1
1089137
no rank
node6775.members.0.js

genus
2
2560142

2560650
2
species

node6778.members.0.js
no rank
1969841
2

genus
1623303
8

2267560
8
no rank

species
node6781.members.0.js
8
1385385

1982113
1
genus

species
1
1982148

1
1486472
no rank
node6784.members.0.js

2560144
2
genus

2560501
2
species

no rank
node6787.members.0.js
2
2047872

2
2560106
genus
node6788.members.0.js
1

1
2560487
species

node6790.members.0.js
no rank
1838063
1

genus
1
1623306

no rank
node6792.members.0.js
1
2562717

phylum
2
2731361

class
2731363
2

order
2
548681

family
10292
2

1
10293
subfamily

genus
10294
1

species
node6799.members.0.js
1
35243

10374
1
subfamily

1
548687
genus

138184
1
node6802.members.0.js
species
