# Supplementary material for: Microbiota composition data of imago and larval stage of the anhydrobiotic midge
Source: Data Brief. 2020 Nov 18;33:106527. doi: 10.1016/j.dib.2020.106527 (PMC7689402; doi:10.1016/j.dib.2020.106527)
Supplement: Supplementary file 6 [file mmc6.zip › mmc6/file_for_krona_visualization_of_larvae.fig3.html]

Javascript must be enabled to view this page.

members
magnitude
magnitudeUnassigned
count
unassigned
taxon
rank

Pv11\_5.0\_lane78\_R1.fq.sorted.unmapped.filtered.from.r

176911
node0.members.0.js
207381

139
superkingdom
2157

35
clade
1783275

phylum
29
28889

29
class
183924

order
6
114380

2272
6
family

56635
6
genus

229980
species
6

strain
node9.members.0.js
6
1198449

3
order
871006

871007
1
family

105850
1
genus

1
species
242703

666510
1
node14.members.0.js
strain

255472
family
2

2
genus
200414

200415
2
species

strain
2
node18.members.0.js
1056495

2281
20
order

118883
20
family

12914
genus
14

312539
9
species

619593
9
node23.members.0.js
strain

species
node24.members.0.js
2
41673

3
node25.members.0.js
species
282676

3
genus
2284

no rank
1
3
node27.members.0.js
2641160

node28.members.0.js
1
species
2663019

species
node29.members.0.js
1
1891280

69655
2
genus

69656
species
2
node31.members.0.js

41674
genus
1

41675
species
node33.members.0.js
1

651137
phylum
6

1643678
class
1

1
order
1033996

family
1
1033997

1826864
genus
1

1798806
species
node39.members.0.js
1

651142
no rank
1

genus
1
1078904

1078905
node42.members.0.js
1
species

order
4
31932

338190
family
4

genus
4
338191

1580092
species
4
node46.members.0.js

1935183
clade
3

3
phylum
1655434

2732160
3
genus

node50.members.0.js
3
species
2594042

28890
101
phylum

class
1
183980

1
order
2231

2232
1
family

genus
1
2233

1316941
species
1

387631
node57.members.0.js
1
strain

clade
8
2283796

class
8
183967

1235850
order
2

1577788
family
2

1607807
1
genus

1
node63.members.0.js
species
1577791

1
genus
1080709

1
node65.members.0.js
species
1406512

6
order
2301

family
1
46659

1
genus
2302

1
species
50339

273116
strain
1
node70.members.0.js

90142
family
2

genus
node72.members.0.js
2
74968

family
3
46630

46631
genus
3

82076
3
species

263820
strain
node76.members.0.js
3

2290931
clade
46

183963
class
21

3
order
1644060

3
family
1644061

253106
genus
1

745377
1
node82.members.0.js
species

2256
genus
2

species
2
44930

797304
strain
2
node85.members.0.js

2235
11
order

38063
no rank
1

1071085
1
node88.members.0.js
species

no rank
3
1963280

genus
3
2732368

1710541
species
3
node91.members.0.js

2236
family
4

1
genus
1070314

2643768
1
no rank

2505977
1
node95.members.0.js
species

genus
1
1656823

1604004
1
node97.members.0.js
species

1075398
1
genus

2648404
1
no rank

2599399
species
1
node100.members.0.js

1
genus
2239

1
no rank
2668073

node103.members.0.js
1
species
751944

1963268
family
3

genus
1
146825

146826
1
species

519442
node107.members.0.js
1
strain

2144190
no rank
1

1679096
species
node109.members.0.js
1

genus
1
node110.members.0.js
203135

1644055
order
7

3
family
1644056

1
genus
293431

293091
species
node114.members.0.js
1

genus
1
node115.members.0.js
2
2251

35746
species
node116.members.0.js
1

1963271
4
family

1209988
1
genus

1048396
1
node119.members.0.js
species

56688
3
node120.members.0.js
1
genus

no rank
1
2642239

node122.members.0.js
1
species
2497325

29284
1
node123.members.0.js
species

224756
25
class

order
4
2191

88404
2
family

2
genus
2192

2
species
83984

410358
strain
node129.members.0.js
2

family
2
2194

230355
genus
1

54120
1
species

679926
1
node133.members.0.js
strain

45989
1
genus

83986
1
species

1
node136.members.0.js
strain
1201294

94695
order
21

2206
family
21

2207
19
genus

species
2
418008

strain
node141.members.0.js
2
1434110

2
species
2208
node142.members.0.js
4

2
node143.members.0.js
strain
1434107

38027
species
node144.members.0.js
1

1
no rank
2644672

1434099
node146.members.0.js
1
species

2215
species
2

node148.members.0.js
2
strain
1434123

2209
8
node149.members.0.js
species

species
1
2214

1
node151.members.0.js
strain
188937

genus
2
2175

2
species
2176

547558
strain
node154.members.0.js
2

clade
28
2283794

183939
15
class

2182
order
15

15
family
196117

196118
15
genus

2641196
1
no rank

node161.members.0.js
1
species
644281

2190
13
species

243232
13
node163.members.0.js
strain

73913
1
species

579137
strain
1
node165.members.0.js

183925
13
class

13
order
2158

13
family
2159

2160
4
genus

species
node170.members.0.js
1
118062

868131
species
1
node171.members.0.js

877455
2
node172.members.0.js
species

2172
8
genus

node174.members.0.js
2
species
294671

4
no rank
2638681

species
4
node176.members.0.js
1609968

species
node177.members.0.js
1
230361

83816
species
1

strain
1
node179.members.0.js
634498

2316
1
genus

1
node181.members.0.js
species
2317

183968
class
18

order
18
2258

family
18
2259

2263
genus
11

2265
5
species

523849
5
node187.members.0.js
strain

311400
1
species

strain
node189.members.0.js
1
69014

2627626
5
no rank

5
node191.members.0.js
species
1674923

2260
genus
7

53953
1
species

70601
node194.members.0.js
1
strain

6
node195.members.0.js
species
2261

no rank
node196.members.0.js
2634
28384

2
node197.members.0.js
27598
2119
superkingdom

4
phylum
200930

68337
class
4

191393
order
4

family
4
191394

2351
3
genus

2352
3
species

3
node204.members.0.js
strain
717231

53572
1
genus

197162
1
species

strain
1
node207.members.0.js
639282

57723
phylum
2

204432
2
class

1
order
332160

332161
family
1

332162
1
genus

1
species
332163

node214.members.0.js
1
isolate
234267

204433
order
1

1
family
204434

1
genus
392733

870903
1
species

401053
strain
1
node219.members.0.js

4
phylum
200918

class
4
188708

2419
3
order

1643950
3
family

2420
genus
3

species
2
2421

484019
strain
2
node226.members.0.js

species
1
node227.members.0.js
46541

order
1
1643947

1
family
1643949

1
genus
1511648

1006576
node231.members.0.js
1
species

1783257
clade
108

phylum
2
92
node233.members.0.js
203682

7
class
666505

1546154
1
no rank

species
node236.members.0.js
1
2528035

666506
2
order

666507
family
2

666508
2
genus

2
species
547188

1142394
strain
node241.members.0.js
2

2483366
order
4

2483367
family
4

2483368
4
genus

1941349
species
node245.members.0.js
3

node246.members.0.js
1
species
1940790

1
class
203683
42
node247.members.0.js

112
4
order

no rank
1
466154

2053591
1
node250.members.0.js
species

family
3
126

genus
3
118

3
no rank
2648843

species
1
node254.members.0.js
1632864

2
node255.members.0.js
species
1636152

2691354
32
order

family
7
2691357

7
genus
123

7
species
125

530564
strain
7
node260.members.0.js

2691359
family
25

2691521
no rank
25

2528024
species
25
node263.members.0.js

2691356
5
order

family
1
5
node265.members.0.js
1763524

1763521
2
genus

1387353
species
2
node267.members.0.js

genus
1
1511635

406548
node269.members.0.js
1
species

1
genus
466152

466153
1
species

886293
1
node272.members.0.js
strain

473814
node273.members.0.js
41
7
no rank

node274.members.0.js
1
species
2528023

species
1
node275.members.0.js
2527975

1
node276.members.0.js
species
2528021

species
node277.members.0.js
1
2527982

species
node278.members.0.js
4
2527976

node279.members.0.js
1
species
2528020

species
node280.members.0.js
1
2527963

species
2
node281.members.0.js
2527974

1930276
species
1
node282.members.0.js

1
node283.members.0.js
species
1930275

species
8
node284.members.0.js
2596890

2527994
species
7
node285.members.0.js

node286.members.0.js
4
species
2528009

species
node287.members.0.js
1
2527997

256845
phylum
2

1313211
class
2

278082
2
order

no rank
2
1674876

2
node292.members.0.js
species
2094242

204428
4
phylum

204429
4
class

3
order
1963360

92713
family
3

112987
1
genus

2643326
no rank
1

1353976
node299.members.0.js
1
species

83551
2
genus

83552
species
2

strain
node302.members.0.js
2
765952

51291
order
1

1
family
809

no rank
1
1113537

genus
1
810

1
species
83554

1050221
1
node308.members.0.js
strain

74201
phylum
10

class
1
414999

415001
order
1

family
1
415002

442430
1
genus

species
1
395922

1
node315.members.0.js
strain
583355

1955630
class
7

7
order
717963

717964
family
7

511745
7
genus

511746
7
species

481448
strain
node321.members.0.js
7

2
class
203494

order
2
48461

1647988
family
1

1
genus
239934

node326.members.0.js
1
species
1679444

1
family
203557

1
genus
2735

1
species
2736

240016
strain
node330.members.0.js
1

2323
2
no rank

1783234
clade
2

95901
1
clade

1
class
2497643

2497644
1
order

2497645
family
1

genus
1
1551504

673862
species
node338.members.0.js
1

95818
1
phylum

genus
1
1331051

species
node341.members.0.js
1
1332188

clade
8124
1783270

1
clade
68336
8119
node343.members.0.js

1
phylum
1090

class
1
191410

191411
order
1

191412
1
family

no rank
1
274493

genus
1
1099

1
species
34090

324925
1
node351.members.0.js
strain

phylum
2806
node352.members.0.js
8117
976

2954
class
117743

200644
2954
node354.members.0.js
2
order

49546
2896
node355.members.0.js
430
family

genus
13
286104

2615021
13
no rank

754409
5
node358.members.0.js
species

species
4
node359.members.0.js
1249933

1936080
species
4
node360.members.0.js

genus
8
527198

8
no rank
2643887

node363.members.0.js
8
species
2027857

237
351
node364.members.0.js
47
genus

1678728
species
9
node365.members.0.js

2547394
species
node366.members.0.js
10

1492737
18
node367.members.0.js
species

33
species
986
41
node368.members.0.js

376686
strain
node369.members.0.js
8

species
9
node370.members.0.js
2518177

1763534
species
node371.members.0.js
17

683124
species
10
node372.members.0.js

16
node373.members.0.js
species
459526

1355330
species
node374.members.0.js
1

1784713
1
node375.members.0.js
species

species
node376.members.0.js
9
2602769

1306519
species
node377.members.0.js
25

2201181
species
3
node378.members.0.js

species
1
312277

node380.members.0.js
1
strain
1094466

55197
12
species

1034807
strain
node382.members.0.js
12

1617283
species
17
node383.members.0.js

86
no rank
196869

species
node385.members.0.js
10
1179672

species
38
node386.members.0.js
2249356

node387.members.0.js
18
species
935222

2478552
node388.members.0.js
20
species

species
2
node389.members.0.js
2183896

species
8
node390.members.0.js
96345

996
node391.members.0.js
4
species

2172098
species
node392.members.0.js
5

75
node393.members.0.js
52959
genus
4

species
13
node394.members.0.js
996801

49
node395.members.0.js
196858
no rank
1

1
node396.members.0.js
species
2686361

4
node397.members.0.js
species
754397

1
node398.members.0.js
species
313598

species
7
node399.members.0.js
2058137

species
12
node400.members.0.js
1336804

species
15
node401.members.0.js
1896175

node402.members.0.js
8
species
1855336

9
node403.members.0.js
species
1774273

genus
444
node404.members.0.js
1129
59732

species
11
node405.members.0.js
112234

species
55
node406.members.0.js
250

node407.members.0.js
6
species
1241979

26
node408.members.0.js
species
1241981

1685010
species
node409.members.0.js
17

42
node410.members.0.js
species
1241978

1265445
species
13
node411.members.0.js

266749
species
5
node412.members.0.js

558152
node413.members.0.js
12
species

species
node414.members.0.js
6
246

2593645
node415.members.0.js
153
28
no rank

species
1
node416.members.0.js
1871047

2594269
3
node417.members.0.js
species

node418.members.0.js
26
species
2015076

2713414
species
1
node419.members.0.js

2547600
node420.members.0.js
6
species

node421.members.0.js
9
species
2487063

1721091
species
node422.members.0.js
1

878220
8
node423.members.0.js
species

2487073
node424.members.0.js
4
species

2487065
species
11
node425.members.0.js

2487071
species
7
node426.members.0.js

2487064
species
node427.members.0.js
26

2478663
14
node428.members.0.js
species

2039166
8
node429.members.0.js
species

108
node430.members.0.js
species
2497456

5
node431.members.0.js
species
254

253
164
node432.members.0.js
species

536441
node433.members.0.js
4
species

species
7
node434.members.0.js
421525

species
node435.members.0.js
9
1324352

node436.members.0.js
6
species
1124835

7
node437.members.0.js
species
266748

1493872
species
13
node438.members.0.js

1241982
species
node439.members.0.js
2

species
node440.members.0.js
14
651561

2
genus
2700084

node442.members.0.js
2
species
1850246

111500
18
genus

species
15
111501

886377
15
node445.members.0.js
strain

species
3
node446.members.0.js
516051

2
genus
59734

247
2
node448.members.0.js
species

1862153
1
genus

1
node450.members.0.js
species
1703343

2
genus
1209327

1803846
node452.members.0.js
2
species

389486
genus
5

5
no rank
2648869

1453352
node455.members.0.js
5
species

28250
6
genus

28251
species
6
node457.members.0.js

417127
genus
60

species
60
398743

strain
60
node460.members.0.js
655815

13
no rank
61432

1871037
node462.members.0.js
1
species

species
node463.members.0.js
10
2584122

1
node464.members.0.js
species
1150389

531844
species
node465.members.0.js
1

node466.members.0.js
6
326319
genus
5

species
1
326320

1300343
1
node468.members.0.js
strain

3
genus
358023

1622118
node470.members.0.js
3
species

node471.members.0.js
15
363408
genus
2

node472.members.0.js
2
species
331648

no rank
11
2615035

species
1
node474.members.0.js
2058134

1336802
node475.members.0.js
10
species

153265
7
genus

2615031
no rank
5

2494375
node478.members.0.js
5
species

101385
2
species

746697
strain
2
node480.members.0.js

22
genus
1518147

22
node482.members.0.js
species
1790137

genus
8
261827

1
no rank
2615009
node484.members.0.js
3

1
node485.members.0.js
species
2686365

species
1
node486.members.0.js
2686366

1736674
species
5
node487.members.0.js

genus
18
28
node488.members.0.js
1016

no rank
2
2640652

species
1
node490.members.0.js
1316596

1
node491.members.0.js
species
2545799

species
1
node492.members.0.js
45243

1848904
node493.members.0.js
1
species

28188
species
node494.members.0.js
3

species
node495.members.0.js
3
1017

1204360
10
genus

762954
10
species

1454006
node498.members.0.js
10
strain

15
genus
308865
node499.members.0.js
53

no rank
8
2685307

2583851
species
node501.members.0.js
8

1756150
species
1
node502.members.0.js

238
5
node503.members.0.js
species

1117645
species
node504.members.0.js
24

genus
73
291183

no rank
73
2647285

60
node507.members.0.js
species
2057808

13
node508.members.0.js
species
983544

genus
23
34084

17
species
34085
23
node510.members.0.js

1228997
node511.members.0.js
6
strain

1649495
genus
6

2622645
6
no rank

1936081
6
node514.members.0.js
species

genus
6
393005

2614803
6
no rank

2069432
6
node517.members.0.js
species

genus
1
178469

616991
node519.members.0.js
1
species

59735
genus
8

species
8
node521.members.0.js
1585976

genus
7
node522.members.0.js
156
76831

256
species
1

strain
node524.members.0.js
1
929704

76832
species
node525.members.0.js
148

44
genus
221065

2685202
15
no rank

2282170
15
node528.members.0.js
species

species
node529.members.0.js
29
1218801

6
genus
2049301

2049305
6
node531.members.0.js
species

genus
3
244698

no rank
3
2615025

node534.members.0.js
3
species
1336796

292691
19
genus

1913577
species
node536.members.0.js
5

node537.members.0.js
3
species
2126553

6
no rank
2615027

1250231
node539.members.0.js
5
species

1250205
species
1
node540.members.0.js

species
1
411153

411154
strain
1
node542.members.0.js

1486245
species
4

strain
node544.members.0.js
4
1229726

genus
1
1013

1014
species
1
node546.members.0.js

genus
20
501783

node548.members.0.js
20
species
237258

2058174
1
genus

2631190
1
no rank

1
node551.members.0.js
species
2585771

node552.members.0.js
15
143222
genus
1

node553.members.0.js
5
species
143223

2633436
no rank
9

1729720
node555.members.0.js
9
species

336276
55
genus

3
no rank
2615019

node558.members.0.js
3
species
2058135

52
node559.members.0.js
species
639310

5
genus
1778601

5
no rank
2630820

2500547
node562.members.0.js
5
species

genus
5
290174

5
no rank
2627091

1714860
1
node565.members.0.js
species

4
node566.members.0.js
species
1714848

225842
25
node567.members.0.js
5
genus

no rank
12
2644710

1798225
species
node569.members.0.js
4

8
node570.members.0.js
species
2686363

node571.members.0.js
5
species
2594004

320324
species
3

1347342
node573.members.0.js
3
strain

104264
39
node574.members.0.js
6
genus

species
9
node575.members.0.js
979

no rank
6
2634405

6
node577.members.0.js
species
2686362

10
node578.members.0.js
76594
species
4

node579.members.0.js
6
strain
1348584

59600
species
8

8
node581.members.0.js
strain
688270

8
genus
104267
node582.members.0.js
91

node583.members.0.js
23
species
104268

species
7
node584.members.0.js
1850252

669041
species
node585.members.0.js
4

107401
16
node586.members.0.js
species

584609
species
1
node587.members.0.js

2635139
no rank
32

species
26
node589.members.0.js
2358479

754423
species
node590.members.0.js
6

1
genus
83612

57029
species
1

313595
node593.members.0.js
1
strain

252356
genus
16

2615042
no rank
12

1250153
1
node596.members.0.js
species

3
node597.members.0.js
species
2496865

313603
1
node598.members.0.js
species

node599.members.0.js
7
species
1836467

species
node600.members.0.js
4
1178778

genus
6
49277

2615011
no rank
6

2570561
node603.members.0.js
6
species

4
genus
216431

4
species
313588

216432
4
node606.members.0.js
strain

family
4
39782

34098
4
genus

2647581
1
no rank

298656
1
node610.members.0.js
species

species
3
node611.members.0.js
1653831

family
32
1853230

332102
genus
32

191579
species
32

strain
32
node615.members.0.js
755732

7
no rank
313602

4
genus
1940138

species
4
node618.members.0.js
1415657

genus
3
336809

336810
node620.members.0.js
3
species

246874
12
family

267986
12
genus

253245
species
12

node624.members.0.js
12
strain
926562

1755828
family
1

genus
1
1755829

node627.members.0.js
1
species
242600

200643
class
293

53
order
171549
node629.members.0.js
292

3
no rank
333046

511434
genus
3

3
node632.members.0.js
511435
species
2

511995
1
node633.members.0.js
isolate

171550
7
family

239759
7
genus

species
node636.members.0.js
7
2585118

171551
12
family

836
genus
1

node639.members.0.js
1
species
393921

9
genus
1784836

1562970
node641.members.0.js
9
species

genus
2
307628

1642646
2
node643.members.0.js
species

815
family
133

genus
7
node645.members.0.js
133
816

28116
species
node646.members.0.js
79

species
node647.members.0.js
3
28113

376805
species
1

667015
strain
1
node649.members.0.js

3
species
151276

679937
3
node651.members.0.js
strain

1
node652.members.0.js
species
47678

357276
species
node653.members.0.js
1

node654.members.0.js
2
species
329854

821
node655.members.0.js
3
species

818
species
node656.members.0.js
5

817
28
node657.members.0.js
species

no rank
1
185291

1
node659.members.0.js
species
1400053

2005523
12
family

12
genus
346096

12
species
185300

strain
node663.members.0.js
12
694427

1
family
2005519

397864
1
genus

397865
species
1

880074
strain
1
node667.members.0.js

family
2
2005520

294702
2
genus

1642647
2
node670.members.0.js
species

68
family
171552

838
68
genus

10
node673.members.0.js
species
28132

1
species
589436

1236517
strain
1
node675.members.0.js

8
no rank
2638335

8
species
652716

node678.members.0.js
8
strain
575614

13
node679.members.0.js
species
28131

node680.members.0.js
1
species
28135

species
2
node681.members.0.js
1177574

species
33
839

node683.members.0.js
33
strain
264731

1970189
order
1

family
1
1471398

1471399
1
genus

species
1
node687.members.0.js
1168034

class
1423
768503

768507
1423
node689.members.0.js
150
order

family
1
1853234

genus
1
59740

2664020
no rank
1

species
1
node693.members.0.js
1085624

family
1
node694.members.0.js
106
1853232

65
genus
323449

species
2
node696.members.0.js
323450

388950
species
4
node697.members.0.js

species
6
node698.members.0.js
400092

no rank
53
2648980

node700.members.0.js
12
species
2694930

2571030
41
node701.members.0.js
species

89966
23
genus

2502781
4
node703.members.0.js
species

2319843
1
node704.members.0.js
species

2615202
no rank
15

1484116
4
node706.members.0.js
species

1356852
species
node707.members.0.js
1

1484118
species
node708.members.0.js
1

species
node709.members.0.js
7
2496028

node710.members.0.js
1
species
2615203

species
node711.members.0.js
1
2584940

species
node712.members.0.js
3
1411621

1379908
17
genus

16
no rank
2639626

12
node715.members.0.js
species
1379910

1379909
node716.members.0.js
4
species

512763
species
1
node717.members.0.js

1937968
family
35

1937972
35
genus

species
35
999

880071
35
node721.members.0.js
strain

7
family
1501348

7
genus
281119

281120
7
species

452471
strain
node725.members.0.js
7

9
family
563798
147
node726.members.0.js

15
genus
280472

15
species
280473

758820
strain
15
node729.members.0.js

68288
genus
17

species
10
node731.members.0.js
320787

7
species
104

strain
7
node733.members.0.js
880070

genus
24
232244

232259
24
species

node736.members.0.js
24
strain
866536

390846
genus
36

390884
species
12

strain
node739.members.0.js
12
926556

1807691
species
node740.members.0.js
10

species
node741.members.0.js
14
1795355

246875
genus
46

388413
23
node743.members.0.js
species

1727163
node744.members.0.js
23
species

6
no rank
1124781

1433993
genus
6

node747.members.0.js
6
species
2321403

family
64
744
node748.members.0.js
89373

455076
genus
63

2704464
no rank
63

species
node751.members.0.js
63
2704465

148
genus
105

2631759
148
no rank

2259595
species
18
node754.members.0.js

2268026
species
node755.members.0.js
130

2676060
214
genus

species
214
node757.members.0.js
998844

genus
11
861914

2
species
651143

1166018
strain
2
node760.members.0.js

2620963
no rank
9

node762.members.0.js
9
species
1834519

27
genus
2173039

1784714
node764.members.0.js
27
species

16
genus
120831

2625061
no rank
8

species
node767.members.0.js
8
538966

94254
8
species

471854
strain
node769.members.0.js
8

genus
26
978

26
species
985

269798
26
node772.members.0.js
strain

53
genus
319458

316068
species
53

649349
node775.members.0.js
53
strain

genus
12
node776.members.0.js
122
107

1211326
species
node777.members.0.js
34

1178516
14
node778.members.0.js
species

36
no rank
2621999

2520506
node780.members.0.js
28
species

species
8
node781.members.0.js
2666025

1379870
node782.members.0.js
16
species

species
10
node783.members.0.js
2057025

1751870
no rank
50

species
node785.members.0.js
50
1945892

200667
177
family

340671
no rank
32

species
32
node788.members.0.js
1257021

genus
23
446458

23
node790.members.0.js
species
1267423

869806
genus
41

41
species
1006

643867
41
node793.members.0.js
strain

59739
genus
81

46
node795.members.0.js
species
2494373

35
no rank
2637820

35
node797.members.0.js
species
1191459

1853228
class
139

1853229
139
order

10
family
563835
node800.members.0.js
139

1860196
7
genus

661488
species
node802.members.0.js
7

649460
17
genus

17
node804.members.0.js
species
477680

19
genus
1769012

2636138
no rank
18

2545455
species
node807.members.0.js
10

2341117
8
node808.members.0.js
species

1
node809.members.0.js
species
496057

79328
40
genus

node811.members.0.js
4
species
2029983

species
7
79329

strain
node813.members.0.js
7
485918

29
no rank
2619133

2033437
species
10
node815.members.0.js

2703787
species
node816.members.0.js
19

12
genus
354354

12
species
354356

700598
node819.members.0.js
12
strain

genus
2
379899

1176587
2
node821.members.0.js
species

2698688
genus
9

2315862
node823.members.0.js
9
species

1874621
13
genus

species
13
node825.members.0.js
1813871

10
genus
398041

661481
species
3
node827.members.0.js

no rank
7
2639558

2502779
species
node829.members.0.js
7

class
457
117747

200666
457
order

family
53
457
node832.members.0.js
84566

genus
21
929509

species
21
995

929556
strain
node835.members.0.js
21

84568
no rank
24

species
24
node837.members.0.js
1986952

1
genus
376469

2632301
no rank
1

2592345
species
1
node840.members.0.js

127
node841.members.0.js
84567
genus
5

363852
species
node842.members.0.js
4

188932
species
node843.members.0.js
17

332999
species
22
node844.members.0.js

species
6
node845.members.0.js
430522

51
no rank
2628915

36
node847.members.0.js
species
1727164

2578106
species
node848.members.0.js
8

node849.members.0.js
4
species
2482728

2201271
node850.members.0.js
1
species

2
node851.members.0.js
species
2605747

species
22
984

485917
strain
node853.members.0.js
22

1649482
genus
14

151895
14
species

strain
14
node856.members.0.js
762903

16
genus
423349
node857.members.0.js
88

2
node858.members.0.js
species
2027860

species
10
423351

10
node860.members.0.js
strain
714943

12
node861.members.0.js
species
862126

10
node862.members.0.js
species
1550579

no rank
26
2617802

species
node864.members.0.js
3
2703789

1300914
species
node865.members.0.js
13

10
node866.members.0.js
species
2305508

398053
6
node867.members.0.js
species

species
node868.members.0.js
5
652787

1234841
species
1
node869.members.0.js

node870.members.0.js
128
28453
genus
11

3
node871.members.0.js
species
649196

371142
species
3
node872.members.0.js

node873.members.0.js
18
species
1010

84
node874.members.0.js
2609468
no rank
22

2003121
species
node875.members.0.js
1

2557994
species
node876.members.0.js
15

18
node877.members.0.js
species
2713573

species
28
node878.members.0.js
2662364

species
9
node879.members.0.js
259

1773450
genus
1

species
1
node881.members.0.js
496056

1937959
class
45

1936988
order
45

family
32
89374

genus
32
1007

32
species
1008

32
node887.members.0.js
strain
984262

family
13
1937961

13
genus
2349

species
13
2350

13
node891.members.0.js
strain
760192

456828
3
phylum

456826
3
genus

456827
species
3

459349
3
node895.members.0.js
strain

142182
2
phylum

219685
2
class

2
order
219686

219687
family
2

1706036
genus
2

861299
species
node901.members.0.js
2

node902.members.0.js
12484
1783272
clade
29

67819
1
phylum

class
1
1663419

1663425
1
order

family
1
1663426

genus
1
1005038

species
1
1005039

661478
1
node909.members.0.js
strain

45
phylum
201174
node910.members.0.js
9414

49
class
1497346

588673
49
order

320583
49
family

49
genus
191494

49
species
191495

strain
49
node916.members.0.js
469383

class
3
84992

3
order
84993

1
family
633392

2562118
no rank
1

1
node921.members.0.js
species
2664374

84994
2
family

2
genus
53634

2
species
53635

525909
node925.members.0.js
2
strain

84998
class
5

1643822
4
order

family
1
node928.members.0.js
4
1643826

84111
2
genus

84112
2
node930.members.0.js
species

2005386
genus
1

1870985
species
1
node932.members.0.js

84999
order
1

1643824
1
family

genus
1
1380

1382
1
species

521095
node937.members.0.js
1
strain

631
class
1760
9307
node938.members.0.js

414714
6
order

414877
family
6

414878
6
genus

304895
6
species

strain
node943.members.0.js
6
479433

85012
28
order

node945.members.0.js
10
2012
family
1

genus
8
1988

no rank
1
6
node947.members.0.js
2626254

species
5
node948.members.0.js
2591108

species
2
node949.members.0.js
1411117

2019
genus
1

species
1
2020

strain
node952.members.0.js
1
471852

83676
family
11

10
genus
2013

280236
species
3

1235441
strain
node956.members.0.js
3

species
3
53437

1205910
3
node958.members.0.js
strain

node959.members.0.js
4
species
2014

104204
genus
1

2635841
no rank
1

2498135
1
node962.members.0.js
species

7
family
2004

genus
3
83681

2593643
3
no rank

species
1
node966.members.0.js
1909395

species
node967.members.0.js
2
2656914

2000
4
genus

2632669
3
no rank

node970.members.0.js
3
species
2202249

species
1
2001

node972.members.0.js
1
strain
479432

3
order
1217098

3
family
1217100

1
genus
281472
3
node975.members.0.js

species
1
node976.members.0.js
419479

no rank
1
2624397

1798224
species
1
node978.members.0.js

order
184
85011

2062
node980.members.0.js
184
4
family

genus
2
2063

2633591
no rank
1

2018025
species
node983.members.0.js
1

1
species
2066

452652
strain
1
node985.members.0.js

49
genus
1883
178
node986.members.0.js

28894
1
node987.members.0.js
species

1
species
1038928

strain
1
node989.members.0.js
1038929

species
1
node990.members.0.js
2049881

1690221
species
2
node991.members.0.js

species
2
node992.members.0.js
68270

285570
species
1
node993.members.0.js

553510
2
node994.members.0.js
species

68246
species
1

subspecies
1
node996.members.0.js
284034

species group
1
1477431

1
node998.members.0.js
species
1886

1
node999.members.0.js
species
68203

66425
species
2
node1000.members.0.js

83656
species
node1001.members.0.js
2

47716
species
1
node1002.members.0.js

629295
species group
2

species subgroup
1
1482596

1911
1
species

67263
subspecies
node1006.members.0.js
1

1482558
1
species subgroup

species
node1008.members.0.js
1
1908

1940
species
node1009.members.0.js
1

1
species
33903

227882
node1011.members.0.js
1
strain

1912
4
node1012.members.0.js
3
species

311982
node1013.members.0.js
1
subspecies

node1014.members.0.js
1
species
33899

node1015.members.0.js
1
species
68175

408015
1
node1016.members.0.js
species

node1017.members.0.js
2
species
36818

1901
1
node1018.members.0.js
species

379067
1
species

749414
1
node1020.members.0.js
strain

species
node1021.members.0.js
1
47763

2496836
2
node1022.members.0.js
species

no rank
13
55
node1023.members.0.js
2593676

1855352
species
node1024.members.0.js
1

2305220
species
1
node1025.members.0.js

465541
node1026.members.0.js
2
species

species
node1027.members.0.js
3
1841249

1
node1028.members.0.js
species
2662065

1961713
1
node1029.members.0.js
species

2005885
species
1
node1030.members.0.js

2705439
species
1
node1031.members.0.js

1442032
species
node1032.members.0.js
3

2695266
1
node1033.members.0.js
species

1725411
species
1
node1034.members.0.js

2662397
species
node1035.members.0.js
2

862751
species
1
node1036.members.0.js

2203205
species
node1037.members.0.js
1

1661694
node1038.members.0.js
1
species

node1039.members.0.js
1
species
1882757

1
node1040.members.0.js
species
1972846

species
1
node1041.members.0.js
2282738

species
1
node1042.members.0.js
2676871

1
node1043.members.0.js
species
2684468

1736046
1
node1044.members.0.js
species

species
node1045.members.0.js
1
2692234

species
node1046.members.0.js
1
2202000

2
node1047.members.0.js
species
1262452

species
node1048.members.0.js
1
2653200

node1049.members.0.js
1
species
2059884

2184053
1
node1050.members.0.js
species

2203204
1
node1051.members.0.js
species

3
node1052.members.0.js
species
1649184

1
node1053.members.0.js
species
2686304

2175864
species
node1054.members.0.js
1

species
node1055.members.0.js
1
2135430

2153485
node1056.members.0.js
1
species

1
node1057.members.0.js
species
1616117

species
node1058.members.0.js
1
1783515

193462
node1059.members.0.js
2
1
species

node1060.members.0.js
1
strain
1352941

1927
species
node1061.members.0.js
1

362257
species
node1062.members.0.js
1

2
species
1950

55158
2
subspecies

strain
2
node1065.members.0.js
316280

15
species
285450

15
subspecies
149682

1352936
15
node1068.members.0.js
strain

1
node1069.members.0.js
species
116188

1
species
1969

1079985
1
node1071.members.0.js
strain

68192
1
species

1
node1073.members.0.js
subspecies
477245

1437453
species
1
node1074.members.0.js

68249
species
1
node1075.members.0.js

54571
species
5
node1076.members.0.js

68209
species
1
node1077.members.0.js

species
1
42684

1214242
node1079.members.0.js
1
strain

1977088
species
1
node1080.members.0.js

species
node1081.members.0.js
2
1888

2495578
order
1

85033
family
1

2078948
1
genus

species
1
node1085.members.0.js
1891644

2037
order
16

node1087.members.0.js
16
2049
family
1

1654
12
node1088.members.0.js
1
genus

8
no rank
2609248

node1090.members.0.js
1
species
2081702

species
1
node1091.members.0.js
1851395

2
node1092.members.0.js
species
2321394

712122
2
node1093.members.0.js
species

2
species
649739

node1095.members.0.js
2
strain
649743

52774
1
node1096.members.0.js
species

1
node1097.members.0.js
species
111015

species
1
node1098.members.0.js
52771

2529408
1
genus

species
node1100.members.0.js
1
52773

1522056
genus
2

2
node1102.members.0.js
species
1282737

2039638
1
order

1
family
2162846

622681
genus
1

species
1
node1106.members.0.js
1884913

4
order
85009
306
node1107.members.0.js

family
141
31957

genus
2
135
node1109.members.0.js
1912216

species
133
node1110.members.0.js
1747

72763
2
genus

2
no rank
2635419

node1113.members.0.js
2
species
1909732

1278221
genus
1

675864
species
1
node1115.members.0.js

29404
genus
2

29405
1
species

1
node1118.members.0.js
strain
1032480

2619695
no rank
1

2672569
species
1
node1120.members.0.js

1912215
genus
1

species
node1122.members.0.js
1
2057246

161
node1123.members.0.js
85015
family
1

2
genus
2044

node1125.members.0.js
2
species
2045

53387
genus
2

node1127.members.0.js
2
species
546874

182639
1
genus

182640
species
1

479435
1
node1130.members.0.js
strain

2
genus
116071

node1132.members.0.js
2
species
75385

117156
genus
3

117157
node1134.members.0.js
3
species

genus
1
138
node1135.members.0.js
1839

450734
species
3

1300347
3
node1137.members.0.js
strain

2518370
species
node1138.members.0.js
1

129
no rank
2615069

2589074
node1140.members.0.js
2
species

2582905
4
node1141.members.0.js
species

2
node1142.members.0.js
species
196162

species
node1143.members.0.js
2
2575373

species
1
node1144.members.0.js
2662361

species
node1145.members.0.js
118
2558918

species
1
node1146.members.0.js
402297

node1147.members.0.js
3
species
449461

node1148.members.0.js
12
2040
genus
2

3
node1149.members.0.js
species
2041

219314
species
2

strain
2
node1151.members.0.js
585531

2633570
no rank
2

1
node1153.members.0.js
species
2107713

1
node1154.members.0.js
species
2079793

species
node1155.members.0.js
3
1736691

3
order
1643682

85030
3
node1157.members.0.js
1
family

38501
1
genus

1
species
138336

1146883
strain
node1160.members.0.js
1

88138
1
genus

species
1
node1162.members.0.js
477641

order
5
85004

5
family
31953

genus
1
node1165.members.0.js
5
1678

species
1
node1166.members.0.js
35760

species
1
158787

1150461
node1168.members.0.js
1
strain

species
node1169.members.0.js
2
33905

282
order
85007
6767
node1170.members.0.js

5
family
85025
node1171.members.0.js
1242

node1172.members.0.js
51
1827
genus
17

1
node1173.members.0.js
species
103816

species
1
node1174.members.0.js
1830

species
2
node1175.members.0.js
1829

43767
species
4
node1176.members.0.js

1828
species
2

1443893
node1178.members.0.js
2
strain

38310
node1179.members.0.js
5
species

192944
12
no rank

species
node1181.members.0.js
1
1570939

species
node1182.members.0.js
1
2594007

species
1
node1183.members.0.js
1302308

1
node1184.members.0.js
species
935199

species
3
node1185.members.0.js
1990687

1805827
node1186.members.0.js
2
species

species
3
node1187.members.0.js
1033922

132919
1
species

strain
node1189.members.0.js
1
101510

1500843
species
1
node1190.members.0.js

37919
node1191.members.0.js
5
4
species

1
node1192.members.0.js
strain
632772

genus
89
node1193.members.0.js
1186
1817

2213200
node1194.members.0.js
6
species

2637762
no rank
4

node1196.members.0.js
3
species
2382165

1
node1197.members.0.js
species
1047172

2
node1198.members.0.js
species
37329

species
node1199.members.0.js
3
257277

2
species
37330

strain
2
node1201.members.0.js
1415166

species
node1202.members.0.js
5
135487

1824
species
node1203.members.0.js
1066

1823
2
node1204.members.0.js
species

455432
2
node1205.members.0.js
species

3
species
37326
5
node1206.members.0.js

1133849
strain
node1207.members.0.js
2

1653
family
65

node1209.members.0.js
65
1716
genus
6

1032851
1
species

strain
1
node1211.members.0.js
863239

2506452
species
node1212.members.0.js
6

3
species
225326

1121362
3
node1214.members.0.js
strain

species
5
169292

548476
strain
5
node1216.members.0.js

species
1
1404244

strain
node1218.members.0.js
1
1404245

1223514
1
species

1
node1220.members.0.js
strain
1223515

1
node1221.members.0.js
species
161896

152794
species
1

196164
strain
node1223.members.0.js
1

43770
1
node1224.members.0.js
species

1
node1225.members.0.js
species
43771

2624378
no rank
2

node1227.members.0.js
1
species
2675218

species
1
node1228.members.0.js
2080740

401472
species
3
node1229.members.0.js

species
1
38288

585529
strain
1
node1231.members.0.js

species
1
node1232.members.0.js
571915

38305
1
species

strain
node1234.members.0.js
1
1224164

191493
1
species

1
node1236.members.0.js
strain
1437874

1725
1
node1237.members.0.js
species

species
1
node1238.members.0.js
38301

1471400
species
node1239.members.0.js
24

43990
3
node1240.members.0.js
species

family
1
316606

1
genus
286801

286802
1
species

node1244.members.0.js
1
strain
640132

3150
family
85026

3150
node1246.members.0.js
2053
genus
443

1
node1247.members.0.js
species
158898

84595
species
13

1112204
strain
13
node1249.members.0.js

2055
node1250.members.0.js
45
species

node1251.members.0.js
3
species
1004901

36822
species
node1252.members.0.js
2

species
1
node1253.members.0.js
2054

1136941
10
node1254.members.0.js
species

species
4
node1255.members.0.js
84096

no rank
114
node1256.members.0.js
2626
2657482

2059875
node1257.members.0.js
2
species

species
2455
node1258.members.0.js
2698900

2676309
node1259.members.0.js
7
species

species
node1260.members.0.js
1
2597659

1737359
species
node1261.members.0.js
5

337191
species
42
node1262.members.0.js

2420509
species
2
node1263.members.0.js

697024
no rank
2

2
genus
1847725

species
node1266.members.0.js
2
1528099

family
435
node1267.members.0.js
2017
1762

genus
2
node1268.members.0.js
12
670516

species
node1269.members.0.js
1
404941

species
1
node1270.members.0.js
83262

1578165
2
node1271.members.0.js
species

species
6
node1272.members.0.js
36809

1866885
444
node1273.members.0.js
91
genus

3
node1274.members.0.js
species
39692

5
species
1804
10
node1275.members.0.js

350054
4
node1276.members.0.js
strain

278137
node1277.members.0.js
1
strain

species
node1278.members.0.js
1
28047

1431246
species
3
node1279.members.0.js

216929
node1280.members.0.js
72
species

39695
species
2
node1281.members.0.js

758802
3
node1282.members.0.js
species

1799
species
node1283.members.0.js
3

3
species
110539

350058
node1285.members.0.js
3
strain

5
species
1772
node1286.members.0.js
8

1214915
node1287.members.0.js
3
strain

4
node1288.members.0.js
species
1286180

1766
species
node1289.members.0.js
58

species
7
node1290.members.0.js
1534348

species
node1291.members.0.js
3
1797

species
7
node1292.members.0.js
1534349

species
node1293.members.0.js
5
1792

39694
species
1
node1294.members.0.js

species
1
36814

1
node1296.members.0.js
strain
710685

species
3
node1297.members.0.js
370526

1791
5
node1298.members.0.js
species

2
node1299.members.0.js
species
1286181

319707
species
node1300.members.0.js
4

319706
species
6
node1301.members.0.js

5
node1302.members.0.js
species
134601

67081
33
node1303.members.0.js
species

36813
6
node1304.members.0.js
species

146017
44
node1305.members.0.js
species

39688
species
node1306.members.0.js
5

126673
node1307.members.0.js
2
species

1794
node1308.members.0.js
2
species

444597
species
node1309.members.0.js
5

species
2
node1310.members.0.js
1771

12
node1311.members.0.js
species
53462

46351
species
5

1122247
5
node1313.members.0.js
strain

1249101
2
node1314.members.0.js
species

5
species
1810

1354275
node1316.members.0.js
5
strain

1
node1317.members.0.js
species
212765

1800
10
species

710421
node1319.members.0.js
10
strain

1073531
genus
10

875328
species
node1321.members.0.js
1

species
node1322.members.0.js
3
1788

1118379
species
1
node1323.members.0.js

species
node1324.members.0.js
5
29314

genus
182
1114
node1325.members.0.js
1763

459858
5
node1326.members.0.js
species

species
4
node1327.members.0.js
1775

species
4
node1328.members.0.js
386911

1389713
631
node1329.members.0.js
species

species
node1330.members.0.js
6
2094119

39689
species
8
node1331.members.0.js

node1332.members.0.js
4
1781
species
3

216594
strain
node1333.members.0.js
1

species group
9
46
node1334.members.0.js
2249310

292462
species
4
node1335.members.0.js

220927
species
7
node1336.members.0.js

185642
species
10
node1337.members.0.js

1784
species
node1338.members.0.js
3

species
8
node1339.members.0.js
53376

470076
1
node1340.members.0.js
species

4
node1341.members.0.js
species
722731

10
no rank
2642494
node1342.members.0.js
71

species
node1343.members.0.js
2
212767

node1344.members.0.js
2
species
1547487

species
1
node1345.members.0.js
2587868

1936029
node1346.members.0.js
2
species

species
13
node1347.members.0.js
1682113

189918
node1348.members.0.js
2
species

species
35
node1349.members.0.js
2487344

node1350.members.0.js
3
species
1545728

2051552
1
node1351.members.0.js
species

species
node1352.members.0.js
6
1552759

169765
species
node1353.members.0.js
10

node1354.members.0.js
3
species
590652

node1355.members.0.js
10
species
44010

species group
19
77643

1
species
78331

strain
node1358.members.0.js
1
1205675

18
node1359.members.0.js
1773
species
17

1266446
strain
node1360.members.0.js
1

120793
node1361.members.0.js
48
9
species group

701042
node1362.members.0.js
2
species

4
species
1764
19
node1363.members.0.js

243243
node1364.members.0.js
1
strain

subspecies
14
node1365.members.0.js
439334

4
node1366.members.0.js
species
222805

339268
11
species

1041522
strain
node1368.members.0.js
11

560555
node1369.members.0.js
3
species

482462
node1370.members.0.js
13
species

43348
5
node1371.members.0.js
species

1769
node1372.members.0.js
1
species

29311
4
species

4
node1374.members.0.js
strain
1202450

3
node1375.members.0.js
species
1789

1768
node1376.members.0.js
16
species

398694
node1377.members.0.js
15
species

2
genus
2126281

1069220
species
2
node1379.members.0.js

85028
family
2

genus
2
2060

2061
node1382.members.0.js
2
1
species

strain
node1383.members.0.js
1
521096

6
family
85029

37914
node1385.members.0.js
6
1
genus

1
no rank
2617939
node1386.members.0.js
3

712270
species
2
node1387.members.0.js

139021
2
node1388.members.0.js
species

1237
node1389.members.0.js
85006
order
107

family
1
node1390.members.0.js
10
85016

genus
1
665568

545619
node1392.members.0.js
1
species

1
genus
1926259

no rank
1
2649176

2654547
node1395.members.0.js
1
species

genus
2
node1396.members.0.js
7
1707

species
1
1711

446466
node1398.members.0.js
1
strain

4
no rank
2620175

2591145
species
3
node1400.members.0.js

2654191
species
node1401.members.0.js
1

family
4
145358

154116
4
genus

2626815
4
no rank

species
node1405.members.0.js
1
2585135

2483799
species
2
node1406.members.0.js

1
node1407.members.0.js
species
2589797

85019
family
7

1696
node1409.members.0.js
7
3
genus

1703
1
node1410.members.0.js
species

273384
species
1
node1411.members.0.js

1136497
species
node1412.members.0.js
2

85021
node1413.members.0.js
15
1
family

2
genus
53357

53358
node1415.members.0.js
2
species

genus
1
267408

no rank
1
2663846

1658671
1
node1418.members.0.js
species

genus
4
265976

1
species
247333

1123251
strain
1
node1421.members.0.js

1758689
node1422.members.0.js
1
species

1
node1423.members.0.js
species
1078471

1
no rank
2643101

node1425.members.0.js
1
species
2593973

125287
genus
2

1
no rank
2615080

1
node1428.members.0.js
species
2594265

1288636
species
1
node1429.members.0.js

node1430.members.0.js
5
53457
genus
1

1
node1431.members.0.js
species
857417

species
1
node1432.members.0.js
53458

2
node1433.members.0.js
species
262209

4
no rank
577468

genus
4
754249

1133546
species
node1436.members.0.js
4

151
family
85023
996
node1437.members.0.js

1705353
genus
2

species
1
node1439.members.0.js
708131

2623385
no rank
1

species
node1441.members.0.js
1
1987356

518733
genus
4

4
node1443.members.0.js
species
412690

110932
6
genus

2663824
no rank
node1445.members.0.js
3

species
2
3
node1446.members.0.js
1575

59736
subspecies
1

281090
1
node1448.members.0.js
strain

genus
1
76634

2685235
no rank
1

node1451.members.0.js
1
species
2079792

5
node1452.members.0.js
69578
genus
2

2649013
1
node1453.members.0.js
no rank

670052
species
node1454.members.0.js
1

2220095
node1455.members.0.js
1
species

8
genus
1573

8
node1457.members.0.js
28447
species
4

31963
subspecies
node1458.members.0.js
1

33014
node1459.members.0.js
1
subspecies

31964
2
node1460.members.0.js
subspecies

6
genus
190323
8
node1461.members.0.js

1
no rank
2624265

species
1
node1463.members.0.js
2583822

species
1
node1464.members.0.js
150123

9
node1465.members.0.js
33886
genus
2

no rank
3
2609250

2609253
species
node1467.members.0.js
2

2609254
1
node1468.members.0.js
species

1671680
1
node1469.members.0.js
species

2
species
110937

strain
2
node1471.members.0.js
1328866

33888
node1472.members.0.js
1
species

1
no rank
1655488

1
clade
1655489

529881
genus
1

2617988
1
no rank

1855377
1
node1477.members.0.js
species

2
genus
1434018

2621488
no rank
2

species
2
node1480.members.0.js
2592654

genus
1
337004

1
node1482.members.0.js
species
279828

genus
1
1759331

node1484.members.0.js
1
species
1619308

427753
2
genus

2635918
no rank
2

species
1
node1487.members.0.js
2282656

node1488.members.0.js
1
species
2599293

46352
genus
3

species
node1490.members.0.js
1
684552

2
no rank
2615065

2070347
node1492.members.0.js
2
species

447237
1
genus

1
no rank
2626248

1795630
node1495.members.0.js
1
species

genus
1
6
node1496.members.0.js
2034

3
node1497.members.0.js
257496
no rank
2

node1498.members.0.js
1
species
1561023

2
node1499.members.0.js
species
69373

1433997
1
genus

1
no rank
2643261

species
node1502.members.0.js
1
2683590

1
genus
1195526

2419771
1
node1504.members.0.js
species

genus
30
33877

species
node1506.members.0.js
3
2509455

species
node1507.members.0.js
4
453304

6
node1508.members.0.js
species
589382

2
no rank
2639701
17
node1509.members.0.js

species
5
node1510.members.0.js
2585717

node1511.members.0.js
6
species
2592652

species
1
node1512.members.0.js
2498704

2080742
species
3
node1513.members.0.js

2680004
genus
2

species
2
node1515.members.0.js
2419774

235888
genus
18

18
no rank
2632331

species
node1518.members.0.js
1
2508880

species
node1519.members.0.js
17
2079791

1230698
3
genus

species
3
node1521.members.0.js
674079

728
node1522.members.0.js
33882
genus
203

2509458
8
node1523.members.0.js
species

1526412
node1524.members.0.js
4
species

node1525.members.0.js
21
species
162426

84292
node1526.members.0.js
20
species

node1527.members.0.js
7
species
82380

370764
species
node1528.members.0.js
32

2033
species
5

979556
5
node1530.members.0.js
strain

6
node1531.members.0.js
species
743009

936337
4
node1532.members.0.js
species

1072463
20
node1533.members.0.js
species

11
node1534.members.0.js
species
2541726

node1535.members.0.js
11
species
104336

node1536.members.0.js
11
species
904291

no rank
21
node1537.members.0.js
333
2609290

species
node1538.members.0.js
1
1696072

species
node1539.members.0.js
5
912630

node1540.members.0.js
9
species
2048898

node1541.members.0.js
7
species
1906742

2567934
species
node1542.members.0.js
24

species
3
node1543.members.0.js
1938334

species
node1544.members.0.js
35
2483401

1916917
1
node1545.members.0.js
species

species
2
node1546.members.0.js
2070348

2268461
16
node1547.members.0.js
species

1714373
node1548.members.0.js
155
species

3
node1549.members.0.js
species
2606451

11
node1550.members.0.js
species
2614638

2614639
node1551.members.0.js
5
species

species
node1552.members.0.js
12
2014534

367477
species
node1553.members.0.js
7

5
node1554.members.0.js
species
1795053

node1555.members.0.js
6
species
2489212

2603598
node1556.members.0.js
5
species

26
node1557.members.0.js
species
36805

node1558.members.0.js
5
species
273677

300019
species
node1559.members.0.js
1

2
genus
55968

species
2
node1561.members.0.js
1784719

1331736
family
1

1331737
1
genus

2624404
1
no rank

2675754
species
1
node1565.members.0.js

145357
3
family

genus
1
57499

1276
1
species

478801
1
node1569.members.0.js
strain

2
genus
745364

species
2
node1571.members.0.js
571913

3
family
125316

947525
1
genus

no rank
1
2626367

species
node1575.members.0.js
1
2171623

2
genus
84756

84757
2
species

471853
strain
node1578.members.0.js
2

1
family
145360

genus
1
60919

1
species
60920

446469
node1582.members.0.js
1
strain

1268
node1583.members.0.js
73
2
family

6
genus
32207

172042
2
node1585.members.0.js
species

node1586.members.0.js
4
43675
species
2

node1587.members.0.js
2
strain
680646

1663
27
genus

species
5
node1589.members.0.js
2211210

656366
species
4
node1590.members.0.js

node1591.members.0.js
1
species
37928

17
no rank
235627

1806905
species
1
node1593.members.0.js

2578107
node1594.members.0.js
1
species

species
node1595.members.0.js
3
1849032

1
node1596.members.0.js
species
1704044

1494608
2
node1597.members.0.js
species

species
9
node1598.members.0.js
1652545

6
genus
1742989

256701
species
6

strain
node1601.members.0.js
6
861360

1269
node1602.members.0.js
7
4
genus

species
node1603.members.0.js
3
1270

169133
1
genus

no rank
node1605.members.0.js
1
2632435

2
genus
596707

species
2
node1607.members.0.js
37927

57493
node1608.members.0.js
10
2
genus

71999
1
node1609.members.0.js
species

1275
species
3
node1610.members.0.js

species
2
node1611.members.0.js
446860

species
node1612.members.0.js
2
388357

2078575
1
genus

1618207
species
node1614.members.0.js
1

370735
9
genus

no rank
9
2626589

2058657
9
node1617.members.0.js
species

1
genus
1742993
node1618.members.0.js
2

no rank
1
node1619.members.0.js
2647000

85017
3
family

genus
1
157920

species
node1622.members.0.js
1
1710

genus
1
254250

372663
species
1

node1625.members.0.js
1
strain
1300344

186188
genus
1

186189
species
1

446471
node1628.members.0.js
1
strain

85020
9
family

genus
node1630.members.0.js
2
36739

472568
1
genus

472569
node1632.members.0.js
1
species

43668
6
genus

2623841
4
no rank

node1635.members.0.js
4
species
1903186

node1636.members.0.js
1
species
556288

43669
species
1

446465
strain
node1638.members.0.js
1

85022
family
1

43673
1
genus

43674
1
node1641.members.0.js
species

85008
35
order

28056
node1643.members.0.js
35
1
family

1873
19
node1644.members.0.js
5
genus

47872
node1645.members.0.js
1
species

species
node1646.members.0.js
1
1881

no rank
3
2617518

node1648.members.0.js
1
species
2201999

species
1
node1649.members.0.js
2675222

2583243
species
1
node1650.members.0.js

47850
species
1

strain
1
node1652.members.0.js
644283

species
node1653.members.0.js
1
261654

node1654.members.0.js
1
species
299152

285665
species
2
node1655.members.0.js

47858
node1656.members.0.js
1
species

node1657.members.0.js
2
species
291594

479978
node1658.members.0.js
1
species

genus
11
1865

2626549
5
node1660.members.0.js
2
no rank

species
node1661.members.0.js
2
946334

649831
node1662.members.0.js
1
species

1
node1663.members.0.js
species
113562

1867
species
2

457423
strain
node1665.members.0.js
2

1866
1
species

strain
1
node1667.members.0.js
512565

196914
species
2

2
node1669.members.0.js
strain
1246995

2
genus
673534

2
no rank
2631981

2024580
species
1
node1672.members.0.js

node1673.members.0.js
1
species
2071627

1
genus
84593

species
1
1003110

strain
node1676.members.0.js
1
263358

168694
1
genus

species
1
168697

strain
node1679.members.0.js
1
391037

85013
10
order

family
10
74712

1
genus
1854
9
node1682.members.0.js

no rank
2
2632575

species
1
node1684.members.0.js
298653

710111
species
1
node1685.members.0.js

2
node1686.members.0.js
species
2714109

2
species
1859

strain
2
node1688.members.0.js
326424

298654
species
1
node1689.members.0.js

species
node1690.members.0.js
1
106370

1434010
genus
1

2629395
1
no rank

1
node1693.members.0.js
species
1907575

order
2
622450

2
family
622451

1849
2
genus

species
2
node1697.members.0.js
414996

1643684
order
16

85031
16
family

16
genus
53460

16
species
53461

479431
strain
node1702.members.0.js
16

85010
56
order

node1704.members.0.js
56
2070
family
4

674734
genus
1

1
no rank
2636053

1
node1707.members.0.js
species
1653480

1813
13
node1708.members.0.js
3
genus

2
node1709.members.0.js
species
33910

129921
node1710.members.0.js
2
species

1814
1
species

1068978
node1712.members.0.js
1
strain

1804986
3
node1713.members.0.js
species

no rank
2
2618356

1896961
node1715.members.0.js
2
species

1851
genus
12

1
species
40988

882081
node1718.members.0.js
1
strain

1852
1
species

strain
1
node1720.members.0.js
471857

2634184
1
no rank

1
node1722.members.0.js
species
2528243

40990
9
species

9
node1724.members.0.js
strain
928724

2071
genus
1

103731
species
1

1
node1727.members.0.js
strain
1179773

165301
2
genus

1586287
2
node1729.members.0.js
species

65496
genus
3

species
node1731.members.0.js
2
1470176

340345
species
1
node1732.members.0.js

2029
genus
2

860235
species
2
node1734.members.0.js

1
genus
142577

species
1
node1736.members.0.js
530584

genus
3
1835

species
node1738.members.0.js
2
2665642

species
1
1836

405948
node1740.members.0.js
1
strain

1847
14
genus

node1742.members.0.js
14
2619320
no rank
3

species
node1743.members.0.js
8
1690815

1
node1744.members.0.js
species
1096868

445576
node1745.members.0.js
1
species

species
node1746.members.0.js
1
1641402

class
2
84995

84996
2
order

84997
2
family

genus
2
42255

42256
species
1
node1751.members.0.js

49319
1
node1752.members.0.js
species

3
class
908620

1755823
order
1

1755824
family
1

genus
1
1755825

1670830
species
node1757.members.0.js
1

order
2
1747768

family
2
1747769

1747770
genus
2

species
node1761.members.0.js
2
1670831

1798711
466
clade

4
phylum
1117
node1763.members.0.js
466

1890505
2
order

2
family
1890528

2
genus
54298

species
2
54299

2
node1768.members.0.js
strain
251229

52604
2
order

1890500
1
family

genus
1
44474

54308
1
species

strain
node1773.members.0.js
1
118163

1
family
1890498

102115
genus
1

1
no rank
2642663

1807358
species
1
node1777.members.0.js

1983111
3
no rank

species
2
node1779.members.0.js
1763363

1
species
718217

forma specialis
1
node1781.members.0.js
1228987

304
subclass
1301283

205
order
1150

44887
no rank
159

864702
species
node1785.members.0.js
159

1892254
family
2

genus
1
1158

species
1
482564

strain
1
node1789.members.0.js
179408

1155738
genus
1

1155739
species
1

1454205
1
node1792.members.0.js
strain

family
3
1892255

genus
3
241421

3
species
241425

3
node1796.members.0.js
strain
1173022

41
family
1892252

genus
38
1205

1206
species
38

node1800.members.0.js
38
strain
203124

genus
1
35823

118562
species
1

459495
strain
node1803.members.0.js
1

genus
2
54304

1160
2
species

388467
node1806.members.0.js
2
strain

order
99
1118

27
family
1890464

genus
4
268175

2648896
4
no rank

4
node1811.members.0.js
species
2005460

669357
genus
16

species
2
669359

strain
node1814.members.0.js
2
113355

2625037
no rank
14

1615909
7
node1816.members.0.js
species

species
node1817.members.0.js
7
1617448

102231
7
genus

2623012
7
no rank

1173026
species
node1820.members.0.js
7

1890449
family
30

1125
30
genus

2643300
no rank
29

1967666
29
node1824.members.0.js
species

1126
species
1
node1825.members.0.js

1890452
family
21

102234
genus
21

species
21
379064

755178
node1829.members.0.js
21
strain

21
family
1890450

2546365
1
genus

2546366
species
1

strain
node1833.members.0.js
1
395962

92682
no rank
2

genus
2
76023

no rank
2
2632878

65093
2
node1837.members.0.js
species

28070
genus
16

2546359
species
12

497965
12
node1840.members.0.js
strain

species
4
2546356

65393
strain
4
node1842.members.0.js

263510
2
genus

2546360
2
species

2
node1845.members.0.js
strain
43989

1890424
28
order

13
family
1213

genus
1
13
node1848.members.0.js
1218

1
species
1219
node1849.members.0.js
11

strain
node1850.members.0.js
2
93060

167546
strain
node1851.members.0.js
1

node1852.members.0.js
4
strain
167542

142479
subspecies
2

59919
strain
2
node1854.members.0.js

167555
1
node1855.members.0.js
strain

no rank
1
node1856.members.0.js
2627481

10
family
1890426

genus
node1858.members.0.js
1
146785

167375
1
genus

no rank
1
2627006

node1861.members.0.js
1
species
1851505

genus
2
node1862.members.0.js
8
1129

node1863.members.0.js
6
2626047
no rank
2

node1864.members.0.js
2
species
585423

species
node1865.members.0.js
1
1916956

node1866.members.0.js
1
species
585425

family
5
2303730

genus
5
170610

1209493
5
species

1641165
node1870.members.0.js
5
strain

node1871.members.0.js
123
1161
order
1

family
9
1892259

244599
8
genus

312883
species
8

1973482
strain
node1875.members.0.js
8

752201
1
genus

289435
1
species

strain
1
node1878.members.0.js
1973480

1892263
family
2

1190
2
genus

494603
no rank
node1881.members.0.js
2

2
family
1182

1203
genus
2

2618749
no rank
2

species
1
node1885.members.0.js
1137095

species
node1886.members.0.js
1
2005464

1185
family
2

373984
genus
1

2676603
1
no rank

node1890.members.0.js
1
species
373994

188910
genus
1

1197
1
species

1
node1893.members.0.js
strain
1973485

83
family
1162

1177
node1895.members.0.js
82
2
genus

374162
species
1

strain
1
node1897.members.0.js
1973483

272131
24
species

63737
node1899.members.0.js
24
strain

2593658
no rank
55

317936
2
node1901.members.0.js
species

28072
species
50
node1902.members.0.js

species
node1903.members.0.js
1
1869241

76335
node1904.members.0.js
2
species

1163
1
genus

2619674
no rank
1

node1907.members.0.js
1
species
1647413

2661849
24
family

1186
24
genus

4
no rank
2619626
node1910.members.0.js
18

1954171
species
8
node1911.members.0.js

99598
species
node1912.members.0.js
6

species
5
1973486

strain
node1914.members.0.js
5
1973488

species
1
938406

strain
node1916.members.0.js
1
1973478

1239
node1917.members.0.js
2329
10
phylum

526524
class
5

5
order
526525

family
5
128827

1
genus
1573534

1
species
39483

strain
1
node1923.members.0.js
717960

1647
2
genus

1514105
2
node1925.members.0.js
species

1918538
1
genus

2636055
1
no rank

2584943
species
1
node1928.members.0.js

1
node1929.members.0.js
genus
2057233

1737404
76
class

76
order
1737405

12
family
2042895

12
genus
1505664

1556
12
species

node1935.members.0.js
12
strain
1128398

1570339
family
17

165779
5
genus

1870984
species
node1938.members.0.js
5

1
genus
150022

species
1
1260

node1941.members.0.js
1
strain
525282

162289
11
genus

node1943.members.0.js
11
species
54005

1737406
family
47

genus
3
41273

3
no rank
2638726

2507161
node1947.members.0.js
3
species

species
44
45497

1288971
44
node1949.members.0.js
strain

no rank
1
33974

1
genus
1930845

1871025
species
1
node1952.members.0.js

498
class
186801

order
1
node1954.members.0.js
376
186802

538999
3
no rank

1
family
543314

2060094
genus
1

1
no rank
2623050

node1959.members.0.js
1
species
2697030

539000
family
2

73918
2
node1961.members.0.js
1
genus

2619059
no rank
1

species
node1963.members.0.js
1
2546351

32
family
186804

genus
4
1849828

species
node1966.members.0.js
4
1505

1849822
genus
3

1490
species
node1968.members.0.js
3

genus
25
1870884

1496
node1970.members.0.js
25
species

3
family
31979
305
node1971.members.0.js

1769729
genus
4

species
node1973.members.0.js
4
1498

genus
4
49082

2638829
4
no rank

1041504
species
4
node1976.members.0.js

no rank
2
189971

2082193
species
2
node1978.members.0.js

10
genus
44258

no rank
10
2629145

2576307
node1981.members.0.js
10
species

20
genus
1485
277
node1982.members.0.js

species
1
1538

748727
strain
node1984.members.0.js
1

species
2
node1985.members.0.js
1548

species
1
node1986.members.0.js
169679

29341
species
3
node1987.members.0.js

36745
species
1
node1988.members.0.js

238834
species
1

1552
1
node1990.members.0.js
subspecies

no rank
34
2614128

2587161
species
node1992.members.0.js
1

1
node1993.members.0.js
species
755731

species
2
node1994.members.0.js
2068654

species
7
node1995.members.0.js
641107

node1996.members.0.js
14
species
2212991

2507159
node1997.members.0.js
4
species

species
node1998.members.0.js
5
2483110

68
node1999.members.0.js
1491
species
56

36826
1
no rank

strain
1
node2001.members.0.js
498214

941968
strain
node2002.members.0.js
3

strain
node2003.members.0.js
7
929506

1
no rank
36827

strain
1
node2005.members.0.js
935198

84022
1
node2006.members.0.js
species

node2007.members.0.js
1
species
1216932

1488
node2008.members.0.js
1
species

1502
species
5
node2009.members.0.js

node2010.members.0.js
2
1513
species
1

strain
node2011.members.0.js
1
1231072

84023
species
1

1
node2013.members.0.js
strain
1341692

2
species
1501
node2014.members.0.js
3

86416
1
node2015.members.0.js
strain

1542
node2016.members.0.js
9
1
species

node2017.members.0.js
8
strain
386415

31
node2018.members.0.js
species
1509

1534
species
15
node2019.members.0.js

1519
species
4
node2020.members.0.js

223919
species
node2021.members.0.js
2

species
14
node2022.members.0.js
19
1520

1428454
strain
node2023.members.0.js
5

394958
species
3
node2024.members.0.js

1
species
217159

1
node2026.members.0.js
strain
536227

species
3
node2027.members.0.js
4
1561

1415775
strain
node2028.members.0.js
1

1494
3
node2029.members.0.js
species

node2030.members.0.js
7
species
1492

species
3
node2031.members.0.js
1504

17
node2032.members.0.js
species
182773

species
12
1493

573061
12
node2034.members.0.js
strain

species
2
node2035.members.0.js
46867

2
genus
171003

116090
node2037.members.0.js
2
species

3
genus
1848399

no rank
3
2633479

2599308
node2040.members.0.js
3
species

family
6
2304686

genus
6
2304692

288965
4
species

strain
node2044.members.0.js
4
720554

species
node2045.members.0.js
2
1677857

family
1
541000

1
genus
1263

1
species
1264

697329
node2049.members.0.js
1
strain

186806
family
2

1730
genus
node2051.members.0.js
2

990719
2
family

2
genus
990721

2649046
1
no rank

1
node2055.members.0.js
species
2086585

node2056.members.0.js
1
species
626937

186807
7
family

6
genus
79206

885581
species
3

strain
3
node2060.members.0.js
646529

species
3
1563

3
node2062.members.0.js
strain
768706

278993
genus
1

1
species
863643

strain
1
node2065.members.0.js
635013

186803
17
family

1843210
genus
2

2642604
no rank
2

2696063
node2069.members.0.js
2
species

genus
node2070.members.0.js
3
841

genus
3
572511

no rank
1
2648079

species
node2073.members.0.js
1
2479767

33035
species
2
node2074.members.0.js

genus
1
2719313

species
node2076.members.0.js
1
208479

698776
genus
node2077.members.0.js
1

46205
1
genus

185007
node2079.members.0.js
1
species

186928
5
no rank

4
node2081.members.0.js
species
2093742

1
node2082.members.0.js
species
2109690

1506553
genus
1

29347
species
1

node2085.members.0.js
1
strain
411468

53433
7
order

4
family
972

genus
3
46466

3
no rank
2640322

2382161
species
3
node2090.members.0.js

genus
1
2330

species
1
2331

node2093.members.0.js
1
strain
572479

family
1
53434

42417
genus
1

42422
species
1

748449
node2097.members.0.js
1
strain

no rank
2
387655

1769008
2
genus

2
node2100.members.0.js
species
1323375

115
order
68295

no rank
1
68296

species
1
node2103.members.0.js
2316383

76
family
543371

44000
57
node2105.members.0.js
genus

genus
1
node2106.members.0.js
19
28895

no rank
1
2622527

1550240
species
1
node2108.members.0.js

10
species
1517
node2109.members.0.js
17

698948
node2110.members.0.js
7
strain

227387
32
family

227388
32
genus

species
23
184064

23
node2114.members.0.js
strain
747365

1794699
node2115.members.0.js
9
species

6
node2116.members.0.js
186814
family
1

1
genus
202949

species
1
161154

698762
node2119.members.0.js
1
strain

genus
4
1754

1757
species
1

node2122.members.0.js
1
strain
509192

583357
species
2

subspecies
2
57481

2
node2125.members.0.js
strain
583358

108150
1
species

580331
strain
node2127.members.0.js
1

class
33
909932

909929
order
23

1843490
9
family

365348
9
genus

3
species
365349

node2133.members.0.js
3
strain
1192197

no rank
6
2629460

species
6
node2135.members.0.js
484770

14
family
1843491

158846
genus
14

species
node2138.members.0.js
12
158847

437897
2
node2139.members.0.js
species

7
order
1843489

31977
7
family

genus
1
5
node2142.members.0.js
39948

4
node2143.members.0.js
species
39950

genus
1
2
node2144.members.0.js
29465

node2145.members.0.js
1
species
39778

3
order
1843488

3
family
909930

3
genus
33024

species
3
node2149.members.0.js
33025

91061
node2150.members.0.js
1706
7
class

order
5
912
node2151.members.0.js
1385

family
418
90964

2
genus
227979

1461582
2
node2154.members.0.js
species

genus
12
408
node2155.members.0.js
1279

species
node2156.members.0.js
1
61015

node2157.members.0.js
2
species
214473

species
7
node2158.members.0.js
29378

70258
node2159.members.0.js
4
species

170573
species
3
node2160.members.0.js

46127
species
25
node2161.members.0.js

node2162.members.0.js
1
species
985762

1294
1
node2163.members.0.js
species

1296
node2164.members.0.js
1
species

1280
species
31
node2165.members.0.js

1282
species
node2166.members.0.js
5

29382
species
294
node2167.members.0.js

species
node2168.members.0.js
1
1281

1290
3
node2169.members.0.js
species

species
2
node2170.members.0.js
28035

1
node2171.members.0.js
species
308354

1286
8
node2172.members.0.js
species

species
node2173.members.0.js
3
1288

2
node2174.members.0.js
species
1283

45972
node2175.members.0.js
1
species

69965
node2176.members.0.js
8
1
genus

node2177.members.0.js
5
69966
species
3

458233
node2178.members.0.js
2
strain

species
1
node2179.members.0.js
1855823

no rank
1
2619255

1898474
1
node2181.members.0.js
species

21
no rank
539002

no rank
15
539738

1378
genus
15

1379
2
node2185.members.0.js
species

12
node2186.members.0.js
species
84135

no rank
node2187.members.0.js
1
2624949

539742
no rank
6

1
genus
33986
node2189.members.0.js
6

node2190.members.0.js
5
2644629
no rank
3

1389960
species
node2191.members.0.js
1

360911
1
node2192.members.0.js
species

38
family
186820

1637
genus
37

36
species
1639
node2195.members.0.js
37

node2196.members.0.js
1
strain
393119

1
genus
2755

node2198.members.0.js
1
species
2756

25
family
186818

2
genus
1372
7
node2200.members.0.js

2662419
2
no rank

2058136
species
2
node2202.members.0.js

species
1
161360

1185653
1
node2204.members.0.js
strain

node2205.members.0.js
1
species
1374

192421
species
1
node2206.members.0.js

genus
4
648800

species
2
node2208.members.0.js
4
76853

2
node2209.members.0.js
strain
1002809

1649
genus
6

1650
species
6
node2211.members.0.js

1569
genus
7

1476
6
node2213.members.0.js
species

1571
node2214.members.0.js
1
species

1
genus
651660

1
node2216.members.0.js
species
417367

node2217.members.0.js
325
186817
family
8

genus
1
2675233

species
1
node2219.members.0.js
152268

6
genus
1329200

species
6
node2221.members.0.js
255247

400634
node2222.members.0.js
14
9
genus

2636778
no rank
3

species
1
node2224.members.0.js
2169540

2072025
node2225.members.0.js
2
species

1421
species
1
node2226.members.0.js

species
node2227.members.0.js
1
2086577

84406
29
genus

no rank
22
2620237

403957
species
node2230.members.0.js
1

species
1
node2231.members.0.js
2419842

2700081
species
node2232.members.0.js
16

node2233.members.0.js
4
species
2419841

species
node2234.members.0.js
6
1482

163877
1
node2235.members.0.js
species

51
genus
1386
185
node2236.members.0.js

653685
species group
7

1423
3
node2238.members.0.js
2
species

135461
node2239.members.0.js
1
subspecies

1938374
species subgroup
node2240.members.0.js
1

species
node2241.members.0.js
2
1648923

72361
1
node2242.members.0.js
species

1404
2
node2243.members.0.js
species

1670641
species
13
node2244.members.0.js

1
node2245.members.0.js
species
1467

1
node2246.members.0.js
species
1408

64
node2247.members.0.js
86661
species group
16

node2248.members.0.js
1
species
1392

4
node2249.members.0.js
1428
species
2

no rank
1
180877

527028
strain
node2251.members.0.js
1

529122
1
node2252.members.0.js
strain

species
node2253.members.0.js
1
1890302

5
node2254.members.0.js
species
1405

2750818
8
no rank

2217832
species
node2256.members.0.js
8

1396
18
node2257.members.0.js
10
species

strain
node2258.members.0.js
1
572264

1
node2259.members.0.js
strain
526989

1
node2260.members.0.js
strain
288681

5
node2261.members.0.js
strain
526986

2026186
species
node2262.members.0.js
1

64104
10
node2263.members.0.js
9
species

527000
strain
node2264.members.0.js
1

1441095
species
node2265.members.0.js
13

86664
node2266.members.0.js
1
species

1398
species
node2267.members.0.js
5

185979
12
no rank

2014076
node2269.members.0.js
1
species

node2270.members.0.js
2
species
352858

486398
species
3
node2271.members.0.js

2567941
1
node2272.members.0.js
species

1827146
node2273.members.0.js
2
species

species
node2274.members.0.js
1
1565991

species
node2275.members.0.js
1
1742359

666686
species
1
node2276.members.0.js

1397
1
node2277.members.0.js
species

species
node2278.members.0.js
1
1478

1402861
species
2
node2279.members.0.js

species
1
node2280.members.0.js
632773

35841
5
node2281.members.0.js
species

561879
species
node2282.members.0.js
5

182709
30
genus

182710
node2284.members.0.js
30
species

3
genus
2675231

species
node2286.members.0.js
2
129985

node2287.members.0.js
1
species
279826

genus
1
12
node2288.members.0.js
129337

2642459
11
no rank

node2290.members.0.js
1
species
471223

1233873
node2291.members.0.js
1
species

391290
9
node2292.members.0.js
species

2675230
genus
1

665099
1
species

node2295.members.0.js
1
strain
1196031

150247
1
node2296.members.0.js
genus

1
genus
2675232

species
node2298.members.0.js
1
1193713

1906945
3
genus

1426
species
1
node2300.members.0.js

1295642
species
2
node2301.members.0.js

1055323
genus
1

species
1
node2303.members.0.js
33936

200903
15
genus

no rank
15
2627918

node2306.members.0.js
15
species
2213194

351195
9
genus

1230341
species
9
node2308.members.0.js

genus
1
459532

386490
species
node2310.members.0.js
1

4
genus
45667

1570
species
node2312.members.0.js
4

2651279
genus
1

no rank
1
2651280

2651284
species
node2315.members.0.js
1

186821
3
family

no rank
3
663587

species
3
85683

node2319.members.0.js
3
strain
439292

family
2
node2320.members.0.js
70
186822

no rank
1
234447

species
node2322.members.0.js
1
1882832

9
node2323.members.0.js
55080
genus
6

51101
2
node2324.members.0.js
species

1465
species
1
node2325.members.0.js

4
genus
44249
node2326.members.0.js
58

2044880
species group
1

483937
1
species

1073571
strain
1
node2329.members.0.js

49283
species
node2330.members.0.js
1

61624
species
1

1
node2332.members.0.js
strain
1036673

node2333.members.0.js
2
species
248903

253703
species
node2334.members.0.js
11

1763538
13
node2335.members.0.js
species

4
no rank
185978
10
node2336.members.0.js

species
node2337.members.0.js
1
1536771

species
1
node2338.members.0.js
1566358

1536770
node2339.members.0.js
1
species

2704463
species
1
node2340.members.0.js

1536775
1
node2341.members.0.js
species

1532905
1
node2342.members.0.js
species

1712516
species
6
node2343.members.0.js

species
6
node2344.members.0.js
2707005

2
node2345.members.0.js
species
1178515

1338368
node2346.members.0.js
1
species

186824
7
family

292635
7
genus

37482
node2349.members.0.js
7
species

node2350.members.0.js
787
186826
order
5

81852
family
59

2
genus
51668

51669
node2353.members.0.js
2
species

33969
genus
12

species
12
node2355.members.0.js
33970

genus
2
2737

no rank
1
2648499

1
node2358.members.0.js
species
2571750

node2359.members.0.js
1
species
519472

genus
2
43
node2360.members.0.js
1350

1354
species
1

768486
strain
node2362.members.0.js
1

5
node2363.members.0.js
species
1352

44008
node2364.members.0.js
1
species

node2365.members.0.js
1
species
53345

species
25
node2366.members.0.js
1353

1351
species
6
node2367.members.0.js

2608891
no rank
1

1
node2369.members.0.js
species
2582830

1
node2370.members.0.js
species
53346

family
627
33958

606
node2372.members.0.js
1578
genus
4

node2373.members.0.js
2
species
53444

1193095
1
species

1
node2375.members.0.js
strain
1291742

82688
node2376.members.0.js
6
species

node2377.members.0.js
1
species
2269374

1
node2378.members.0.js
species
468911

species
1
293371

1423778
node2380.members.0.js
1
strain

1138822
species
7
node2381.members.0.js

89059
species
2
node2382.members.0.js

node2383.members.0.js
3
species
1847728

node2384.members.0.js
6
species
1074467

1607
species
1
node2385.members.0.js

species
7
node2386.members.0.js
1296540

1
node2387.members.0.js
species
1589

node2388.members.0.js
3
species
47770

544
species group
655183

544
node2390.members.0.js
species
1582

152331
species
node2391.members.0.js
3

species
1
node2392.members.0.js
109790

2620435
2
no rank

2419772
species
node2394.members.0.js
2

species
node2395.members.0.js
1
1587

species
1
node2396.members.0.js
392416

1600
5
node2397.members.0.js
species

1720083
species
node2398.members.0.js
1

164393
species
1
node2399.members.0.js

species
1
node2400.members.0.js
60520

1253
21
genus

species
1
node2402.members.0.js
187452

3
species
1255
node2403.members.0.js
20

strain
node2404.members.0.js
17
278197

186827
2
family

2689587
genus
2

2
node2407.members.0.js
species
2036206

81850
family
28

46255
26
genus

2506420
19
node2410.members.0.js
species

165096
species
node2411.members.0.js
1

759620
species
node2412.members.0.js
1

species
node2413.members.0.js
4
155866

46256
node2414.members.0.js
1
species

genus
2
1243

1511761
species
node2416.members.0.js
1

species
node2417.members.0.js
1
33964

186828
family
9

191769
3
genus

2632303
3
no rank

species
node2421.members.0.js
3
1911586

genus
3
2747

1
no rank
257487

208596
species
node2424.members.0.js
1

2751
2
node2425.members.0.js
species

genus
2
1470540

2496265
species
1
node2427.members.0.js

2621505
1
no rank

1903686
species
1
node2429.members.0.js

29393
1
genus

29394
node2431.members.0.js
1
species

1300
57
family

1
genus
1357
node2433.members.0.js
15

1366
node2434.members.0.js
1
species

no rank
node2435.members.0.js
1
2643510

1358
node2436.members.0.js
4
2
species

subspecies
2
node2437.members.0.js
1360

node2438.members.0.js
7
species
1940789

1363
species
node2439.members.0.js
1

genus
10
node2440.members.0.js
42
1301

1311
node2441.members.0.js
1
species

1302
node2442.members.0.js
3
species

1
node2443.members.0.js
species
1888195

1304
5
node2444.members.0.js
species

node2445.members.0.js
1
species
1314

1308
node2446.members.0.js
2
species

species group
1
119603

1336
species
1

40041
subspecies
1
node2449.members.0.js

species
node2450.members.0.js
1
1341

82348
node2451.members.0.js
1
species

node2452.members.0.js
1
species
1433513

species
node2453.members.0.js
2
1310

28037
species
2
node2454.members.0.js

1
node2455.members.0.js
species
1335

1305
node2456.members.0.js
7
species

1307
species
2
node2457.members.0.js

1811193
species
node2458.members.0.js
1

226
phylum
544448

226
class
31969

2085
175
order

family
175
2092

genus
170
2093

node2464.members.0.js
1
species
171284

3
no rank
2683645

2683967
node2466.members.0.js
3
species

species
1
node2467.members.0.js
45361

1
species group
656088

2095
species
1

node2470.members.0.js
1
subspecies
40480

2112
species
node2471.members.0.js
1

2096
species
1
node2472.members.0.js

45363
1
node2473.members.0.js
species

2113
node2474.members.0.js
2
species

2109
species
1
node2475.members.0.js

species
1
node2476.members.0.js
142649

species
1
node2477.members.0.js
114885

33923
species
2
node2478.members.0.js

2120
species
9
node2479.members.0.js

51365
species
1

strain
1
node2481.members.0.js
743967

1
node2482.members.0.js
species
2098

node2483.members.0.js
2
species
29554

species
2
node2484.members.0.js
29562

2121
node2485.members.0.js
1
species

171281
species
1
node2486.members.0.js

species
node2487.members.0.js
43
538220

node2488.members.0.js
1
species
48003

node2489.members.0.js
3
species
2094

node2490.members.0.js
2
species
2110

2111
species
node2491.members.0.js
32

node2492.members.0.js
1
species
57372

node2493.members.0.js
28
species
29561

species
node2494.members.0.js
17
2099

519450
5
node2495.members.0.js
species

28227
species
2

272633
strain
2
node2497.members.0.js

2128
1
species

743971
strain
1
node2499.members.0.js

2
node2500.members.0.js
species
2123

5
genus
2129

species
node2502.members.0.js
5
2130

186328
order
44

12
family
33925

genus
6
46238

node2506.members.0.js
5
species
214888

node2507.members.0.js
1
species
81459

1
genus
46239
6
node2508.members.0.js

219745
node2509.members.0.js
2
species

species
node2510.members.0.js
1
216427

2151
species
node2511.members.0.js
1

138853
1
species

strain
1
node2513.members.0.js
81460

family
32
2131

node2515.members.0.js
32
2132
genus
1

362837
species
2
node2516.members.0.js

1
node2517.members.0.js
species
315358

47834
species
1

node2519.members.0.js
1
strain
273035

species
1
node2520.members.0.js
2139

species
2
216933

strain
node2522.members.0.js
2
1276227

2136
4
species

strain
4
node2524.members.0.js
1336748

2637901
no rank
node2525.members.0.js
2

species
4
216935

strain
node2527.members.0.js
4
1276246

2133
species
6
node2528.members.0.js

species
1
2145

1276220
strain
node2530.members.0.js
1

2138
species
node2531.members.0.js
1

1
species
216944

1276257
1
node2533.members.0.js
strain

species
node2534.members.0.js
2
324079

node2535.members.0.js
1
species
216938

216931
species
2
node2536.members.0.js

186329
7
order

2146
7
family

genus
6
33926

1
species group
85620

229545
1
species

node2542.members.0.js
1
strain
322098

85625
5
species group

node2544.members.0.js
5
species
135727

genus
1
2147

node2546.members.0.js
1
species
29552

200795
phylum
13

2
class
292625

292629
2
order

family
2
292628

2019482
2
genus

species
2
node2552.members.0.js
1986204

189775
1
class

189776
1
order

189777
family
1

499
1
genus

1
species
500

309801
strain
node2558.members.0.js
1

class
1
388447

388448
order
1

family
1
2692416

genus
1
2692417

2509675
1
node2563.members.0.js
species

301297
9
class

9
order
1202465

9
family
1202464

61434
genus
9
node2567.members.0.js

1297
phylum
6

class
6
188787

order
1
68933

family
1
188786

270
1
genus

56956
species
node2573.members.0.js
1

order
5
118964

5
family
183710

5
node2576.members.0.js
1298
genus
1

species
1
node2577.members.0.js
317577

1211322
node2578.members.0.js
1
species

2623546
no rank
1

species
1
node2580.members.0.js
2080419

502394
species
1

745776
strain
1
node2582.members.0.js

32066
phylum
101

203490
class
101

order
101
203491

203492
28
family

genus
3
node2587.members.0.js
28
848

1
species
849

469615
1
node2589.members.0.js
strain

861
species
node2590.members.0.js
1

856
2
node2591.members.0.js
species

859
species
node2592.members.0.js
1

2648384
no rank
1

node2594.members.0.js
1
species
671211

3
node2595.members.0.js
1583098
species
1

strain
1
node2596.members.0.js
1307444

1
node2597.members.0.js
strain
1307443

3
node2598.members.0.js
species
2663009

4
species
851
13
node2599.members.0.js

1
subspecies
155615

1307427
strain
1
node2601.members.0.js

subspecies
node2602.members.0.js
2
76857

subspecies
5
6
node2603.members.0.js
76859

457405
strain
node2604.members.0.js
1

73
family
1129771

32067
node2606.members.0.js
43
30
genus

node2607.members.0.js
6
species
157687

no rank
1
2633022

1785996
1
node2609.members.0.js
species

2
node2610.members.0.js
species
554406

species
node2611.members.0.js
3
157688

109328
node2612.members.0.js
1
species

genus
1
2755140

157692
species
node2614.members.0.js
1

genus
29
32068

826
29
species

526218
node2617.members.0.js
29
strain

1224
4531
node2618.members.0.js
209
phylum

205
subphylum
68525

class
42
28221

order
9
213118

213121
family
6

109168
4
genus

species
4
84980

177439
4
node2625.members.0.js
strain

genus
1
427922

1
species
427923

589865
strain
node2628.members.0.js
1

893
1
genus

1
node2630.members.0.js
species
1986146

213119
family
3

1
genus
28222

1
species
28223

651182
node2634.members.0.js
1
strain

2299
genus
2

1
node2636.members.0.js
species
947919

1
node2637.members.0.js
species
571177

213113
order
4

117942
4
family

4
genus
33001

4
species
33002

694431
4
node2642.members.0.js
strain

2
order
69541

family
2
213422

2
genus
28231

313985
species
2

398767
2
node2647.members.0.js
strain

node2648.members.0.js
2
1779134
order
1

2099666
1
no rank

2600177
node2650.members.0.js
1
species

213115
3
order

194924
3
family

1
genus
2035811

1716143
node2654.members.0.js
1
species

872
genus
2

species
1
345370

694327
node2657.members.0.js
1
subspecies

species
1
880

526222
node2659.members.0.js
1
strain

29
22
order

80811
16
suborder

1524215
3
family

3
node2663.members.0.js
161492
genus
1

species
1
161493

node2665.members.0.js
1
strain
290397

1
no rank
2620896

1
node2667.members.0.js
species
404589

31
family
6

83461
1
genus

1
node2670.members.0.js
species
35

32
genus
5

1
species
34
2
node2672.members.0.js

1
node2673.members.0.js
strain
246197

2
species
83455

1278073
node2675.members.0.js
2
strain

species
1
node2676.members.0.js
1297742

7
family
39

47
genus
2

2
node2679.members.0.js
species
48

40
genus
2

2
species
41

378806
2
node2682.members.0.js
strain

2
genus
42

species
2
node2684.members.0.js
43

44
1
genus

83453
species
1

1294270
strain
1
node2687.members.0.js

80812
suborder
6

no rank
1
215910

1649470
1
genus

species
node2691.members.0.js
1
888845

5
family
49

50
genus
1

52
species
1
node2694.members.0.js

39643
genus
4

2
species
56
node2696.members.0.js
4

448385
node2697.members.0.js
2
strain

29547
class
163

213849
order
160

10
family
72293

1
genus
209
node2701.members.0.js
8

35818
species
1
node2702.members.0.js

1591088
species
1
node2703.members.0.js

213
1
node2704.members.0.js
species

210
species
2
node2705.members.0.js

node2706.members.0.js
1
species
45498

138563
1
node2707.members.0.js
species

2
genus
202746

202747
species
1

node2710.members.0.js
1
strain
563040

species
1
39766

node2712.members.0.js
1
strain
326298

150
family
72294

genus
1
69
node2714.members.0.js
194

species
3
827

node2716.members.0.js
3
strain
1032069

1
node2717.members.0.js
species
28080

1244531
species
node2718.members.0.js
2

species
2
node2719.members.0.js
4
201

node2720.members.0.js
1
strain
1388750

1
node2721.members.0.js
strain
306263

497724
3
node2722.members.0.js
1
species

node2723.members.0.js
2
strain
1388752

species
17
196

node2725.members.0.js
17
subspecies
1507806

195
3
species

node2727.members.0.js
2
strain
1183379

node2728.members.0.js
1
strain
1367491

species
2
76517

2
node2730.members.0.js
strain
360107

1965231
4
species

subspecies
1
node2732.members.0.js
1874362

1660067
node2733.members.0.js
3
subspecies

1031542
17
node2734.members.0.js
species

2
species
197
7
node2735.members.0.js

32021
1
node2736.members.0.js
subspecies

3
subspecies
32022

683082
node2738.members.0.js
2
strain

683083
1
node2739.members.0.js
strain

strain
1
node2740.members.0.js
1338035

206
species
2
node2741.members.0.js

374106
species
1

1121267
1
node2743.members.0.js
strain

204
species
node2744.members.0.js
2

3
no rank
2321108
node2745.members.0.js
81

2
genus
28196
node2746.members.0.js
43

2
species
1278212

663365
strain
2
node2748.members.0.js

2320140
node2749.members.0.js
1
species

species
1
node2750.members.0.js
913109

1080223
species
node2751.members.0.js
1

species
node2752.members.0.js
1
255507

603050
11
species

1032238
11
node2754.members.0.js
strain

species
7
544718

7
node2756.members.0.js
strain
1032240

species
node2757.members.0.js
1
1912871

1
species
28199

strain
node2759.members.0.js
1
572480

28197
species
1

strain
1
node2761.members.0.js
1036172

species
2
node2762.members.0.js
877500

197482
node2763.members.0.js
5
species

505249
species
1
node2764.members.0.js

species
3
6
node2765.members.0.js
28198

1032070
strain
node2766.members.0.js
3

2321207
no rank
1

944547
1
node2768.members.0.js
species

2321115
34
genus

34
node2770.members.0.js
species
663364

235899
order
3

224467
3
family

genus
2
191301

species
2
291048

node2775.members.0.js
2
strain
391592

191291
genus
1

1
species
244787

598659
node2778.members.0.js
1
strain

28
class
28216
node2779.members.0.js
841

order
22
206351

1499392
9
family

6
no rank
90153

535
2
genus

394935
species
1
node2784.members.0.js

2641838
no rank
1
node2785.members.0.js

4
genus
32014

4
no rank
235634

2496266
species
4
node2788.members.0.js

genus
1
568394

no rank
1
2642558

748280
1
node2791.members.0.js
species

1
genus
57739

2684990
no rank
1

1
node2794.members.0.js
species
1192162

1
genus
407217

2628611
1
no rank

1590041
node2797.members.0.js
1
species

481
13
family

482
12
genus

node2800.members.0.js
1
species
492

species
1
node2801.members.0.js
326523

species
node2802.members.0.js
1
495

2666100
4
node2803.members.0.js
species

species
node2804.members.0.js
1
326522

1853278
1
node2805.members.0.js
species

2623750
1
no rank

species
node2807.members.0.js
1
1853276

488
node2808.members.0.js
1
species

1
node2809.members.0.js
species
487

1
no rank
421605

node2811.members.0.js
1
species
2052837

node2812.members.0.js
739
80840
order
74

119060
node2813.members.0.js
108
7
family

node2814.members.0.js
31
48736
genus
2

3
node2815.members.0.js
species
305

3
species
329
node2816.members.0.js
16

node2817.members.0.js
13
strain
428406

190721
10
node2818.members.0.js
species

47670
genus
1

47671
1
node2820.members.0.js
species

genus
29
32008

41899
species
node2822.members.0.js
3

species
1
node2823.members.0.js
28095

3
no rank
2613784

758782
1
node2825.members.0.js
species

node2826.members.0.js
1
species
1637862

1
node2827.members.0.js
species
416344

1
species group
111527
2
node2828.members.0.js

57975
species
node2829.members.0.js
1

758793
1
node2830.members.0.js
species

species group
3
19
node2831.members.0.js
87882

species
node2832.members.0.js
2
292

265293
node2833.members.0.js
1
species

2
node2834.members.0.js
species
95486

1
node2835.members.0.js
species
1207504

60550
species
1
node2836.members.0.js

species
node2837.members.0.js
2
101571

87883
node2838.members.0.js
2
species

488729
1
node2839.members.0.js
species

488731
species
2
node2840.members.0.js

488447
species
node2841.members.0.js
1

node2842.members.0.js
1
species
95485

44013
13
genus

1743168
9
node2844.members.0.js
species

species
2
node2845.members.0.js
576610

556054
species
2
node2846.members.0.js

4
genus
106589
16
node2847.members.0.js

96344
species
1
node2848.members.0.js

164546
3
node2849.members.0.js
species

species
2
82541

1267562
strain
node2851.members.0.js
2

species
node2852.members.0.js
2
119219

2
node2853.members.0.js
species
106590

node2854.members.0.js
1
species
68895

species
1
node2855.members.0.js
82633

2
genus
1822464
6
node2856.members.0.js

no rank
2
2615204

node2858.members.0.js
2
species
2571747

species
1
node2859.members.0.js
60548

1
node2860.members.0.js
species
311230

genus
5
93217

node2862.members.0.js
1
species
445709

node2863.members.0.js
1
species
656179

species
1
node2864.members.0.js
1891094

93220
1
node2865.members.0.js
species

species
node2866.members.0.js
1
573737

75682
53
node2867.members.0.js
10
family

963
5
genus

node2869.members.0.js
1
species
2014887

341045
2
species

1262470
2
node2871.members.0.js
strain

2
no rank
2624150

2025949
species
2
node2873.members.0.js

303379
1
genus

204773
node2875.members.0.js
1
species

29580
4
genus

2
species
55508

strain
node2878.members.0.js
2
1349767

2610881
no rank
2

1236179
species
node2880.members.0.js
1

1938606
species
1
node2881.members.0.js

202907
5
genus

4
node2883.members.0.js
species
279058

158899
node2884.members.0.js
1
species

1
genus
1344552

no rank
1
2617509

species
node2887.members.0.js
1
2601898

401469
15
node2888.members.0.js
2
genus

no rank
5
node2889.members.0.js
10
2630295

2058625
species
node2890.members.0.js
3

2058624
2
node2891.members.0.js
species

401471
3
node2892.members.0.js
species

genus
12
149698

1141883
node2894.members.0.js
1
species

321984
1
node2895.members.0.js
species

321985
2
node2896.members.0.js
species

no rank
3
2609279

1678028
species
node2898.members.0.js
1

2
node2899.members.0.js
species
1707785

321983
species
node2900.members.0.js
1

node2901.members.0.js
2
species
945844

species
node2902.members.0.js
2
2045208

family
43
node2903.members.0.js
438
80864

genus
3
52972

species
1
216465

node2906.members.0.js
1
strain
365044

2638319
2
node2907.members.0.js
1
no rank

296591
node2908.members.0.js
1
species

genus
1
232523

80880
species
node2910.members.0.js
1

16
genus
80865
node2911.members.0.js
32

species
1
2
node2912.members.0.js
80866

strain
1
node2913.members.0.js
398578

180282
species
14
node2914.members.0.js

219181
2
genus

2109914
2
node2916.members.0.js
species

genus
2
174951

1
species
94132
node2918.members.0.js
2

strain
1
node2919.members.0.js
365046

1
genus
352450

species
1
node2921.members.0.js
2109915

genus
1
node2922.members.0.js
8
34072

no rank
4
663243

species
node2924.members.0.js
2
434008

node2925.members.0.js
1
species
2126319

282217
node2926.members.0.js
1
species

species
node2927.members.0.js
3
34073

4
genus
28065

2627954
2
no rank

2527691
node2930.members.0.js
2
species

1
node2931.members.0.js
species
2509614

1
node2932.members.0.js
species
1842727

12916
244
node2933.members.0.js
11
genus

species
6
node2934.members.0.js
553814

no rank
15
node2935.members.0.js
224
2684926

358220
species
node2936.members.0.js
151

species
36
node2937.members.0.js
232721

1858609
3
node2938.members.0.js
species

2478662
10
node2939.members.0.js
species

node2940.members.0.js
9
species
1842533

80867
2
species

subspecies
2
node2942.members.0.js
80870

80869
1
node2943.members.0.js
species

1436289
2
genus

1436290
species
2

strain
node2946.members.0.js
2
946483

1
genus
364316

1
species
364317

391735
strain
node2949.members.0.js
1

80
node2950.members.0.js
283
genus
45

225991
species
node2951.members.0.js
3

32013
2
species

1219032
strain
2
node2953.members.0.js

species
2
node2954.members.0.js
16
285

strain
node2955.members.0.js
11
1191062

1392005
strain
3
node2956.members.0.js

2638500
1
no rank

1
node2958.members.0.js
species
2597701

363952
species
node2959.members.0.js
12

1082851
species
node2960.members.0.js
1

12
node2961.members.0.js
47420
genus
2

1763535
node2962.members.0.js
2
species

2610897
node2963.members.0.js
8
3
no rank

species
1
node2964.members.0.js
795665

1842537
node2965.members.0.js
1
species

node2966.members.0.js
3
species
2651974

201096
genus
3

3
node2968.members.0.js
species
179636

995019
1
family

1
genus
40544

species
node2971.members.0.js
1
2584944

1
family
506
13
node2972.members.0.js

1
genus
152267

2626614
1
no rank

2488560
node2975.members.0.js
1
species

106146
2
genus

2652175
2
no rank

node2978.members.0.js
2
species
2652177

1
genus
517
3
node2979.members.0.js

463040
species
1
node2980.members.0.js

35814
species
1
node2981.members.0.js

5
genus
222

85698
species
1
node2983.members.0.js

32002
species
2
node2984.members.0.js

2626865
no rank
2

2282475
node2986.members.0.js
1
species

1758194
node2987.members.0.js
1
species

507
genus
1

species
1
node2989.members.0.js
511

80841
no rank
2

864051
2
node2991.members.0.js
species

node2992.members.0.js
50
224471
no rank
4

65047
1
genus

no rank
1
2648776

1658665
1
node2995.members.0.js
species

318147
genus
1

2642959
no rank
1

1768242
node2998.members.0.js
1
species

1
genus
644355

species
node3000.members.0.js
1
392593

4
genus
92793

1296669
4
node3002.members.0.js
species

32012
genus
3

no rank
1
2625466

1050370
1
node3005.members.0.js
species

926
node3006.members.0.js
2
1
species

75379
strain
node3007.members.0.js
1

5
genus
34102

34103
species
5

node3010.members.0.js
5
subspecies
639200

54066
2
genus

2646274
2
no rank

node3013.members.0.js
2
species
2697032

genus
3
28067

species
3
28068

3
node3016.members.0.js
strain
983917

316612
10
node3017.members.0.js
2
genus

species
8
105560

node3019.members.0.js
8
strain
420662

species
2
node3020.members.0.js
413882

2
genus
212743

946333
species
node3022.members.0.js
2

genus
12
93681

76731
12
node3024.members.0.js
species

order
19
206389

13
family
2008794

12960
7
node3027.members.0.js
2
genus

5
no rank
2629479

species
2
node3029.members.0.js
2067960

62928
species
1
node3030.members.0.js

356837
species
node3031.members.0.js
1

2027405
species
node3032.members.0.js
1

genus
1
6
node3033.members.0.js
33057

59405
1
species

strain
1
node3035.members.0.js
44139

1134435
3
node3036.members.0.js
species

1
no rank
2609274

node3038.members.0.js
1
species
85643

family
3
2008795

73029
3
node3040.members.0.js
1
genus

259537
2
species

node3042.members.0.js
2
strain
159087

75787
family
3

1
genus
551759

551760
1
species

76114
1
node3046.members.0.js
strain

146937
genus
2
node3047.members.0.js

1
no rank
33809

1904640
species
node3049.members.0.js
1

16
order
32003

family
3
206379

914
3
genus

2609265
no rank
1

node3054.members.0.js
1
species
261292

species
1
node3055.members.0.js
44577

species
1
916

335283
1
node3057.members.0.js
strain

90627
family
1

1
genus
1443590

1188319
1
node3060.members.0.js
species

32011
11
family

1
genus
404

405
1
species

strain
node3064.members.0.js
1
265072

node3065.members.0.js
2
genus
81682

1679002
genus
7

2588536
2
node3067.members.0.js
species

node3068.members.0.js
5
species
2588535

1
genus
359407

1
species
1055487

1
node3071.members.0.js
strain
666681

2008790
family
1

919
genus
1

1
species
36861

strain
node3075.members.0.js
1
292415

16
no rank
119066

327159
genus
1

1
species
327160

522306
1
node3079.members.0.js
strain

33055
genus
15

33056
species
node3081.members.0.js
9

species
1
994696

strain
node3083.members.0.js
1
1208920

species
1
994695

1208921
1
node3085.members.0.js
strain

994692
4
species

1208919
strain
4
node3087.members.0.js

93
class
28211
1179
node3088.members.0.js

order
81
204455

19
family
31989
node3090.members.0.js
76

genus
1
366614

195105
1
node3092.members.0.js
species

285107
genus
2

species
2
node3094.members.0.js
1915078

74030
genus
2

no rank
2
2614913

2587846
1
node3097.members.0.js
species

2494550
species
node3098.members.0.js
1

2433
genus
4

42443
4
species

strain
node3101.members.0.js
4
391595

265
node3102.members.0.js
9
3
genus

1
no rank
246570

2560053
species
1
node3104.members.0.js

34003
1
species

1367847
strain
1
node3106.members.0.js

2
node3107.members.0.js
species
147645

1077935
species
2
node3108.members.0.js

1
genus
478070

2648686
1
no rank

node3111.members.0.js
1
species
2590016

299261
1
genus

299262
node3113.members.0.js
1
species

1
genus
1855413

1
node3115.members.0.js
species
1267768

60136
1
genus

no rank
1
196795

species
1
node3118.members.0.js
1389011

1
genus
387095

2639808
no rank
1

2587842
node3121.members.0.js
1
species

58842
1
genus

2624628
1
no rank

2009329
1
node3124.members.0.js
species

1060
6
genus

5
node3126.members.0.js
1063
species
4

557760
strain
node3127.members.0.js
1

species
node3128.members.0.js
1
1075

1
genus
191028

2614906
1
no rank

species
node3131.members.0.js
1
2508307

1649279
1
genus

node3133.members.0.js
1
species
379347

4
genus
1443919

node3135.members.0.js
4
species
2494374

5
no rank
58840

species
2
node3137.members.0.js
2171755

2605946
species
1
node3138.members.0.js

2033435
2
node3139.members.0.js
species

74032
genus
1

species
node3141.members.0.js
1
74033

53945
1
node3142.members.0.js
genus

2
genus
263377

1229727
2
node3144.members.0.js
species

302485
genus
1

1
node3146.members.0.js
species
221822

227873
genus
4

121719
4
node3148.members.0.js
species

genus
1
188905

2645469
1
no rank

species
1
node3151.members.0.js
290400

genus
2
1844498

species
node3153.members.0.js
2
1505036

1955420
genus
2

1826607
species
node3155.members.0.js
2

1097466
2
genus

1335048
node3157.members.0.js
2
species

5
family
69657

74317
4
genus

74318
4
species

4
node3161.members.0.js
strain
394221

85
1
genus

81032
species
1

node3164.members.0.js
1
strain
228405

82117
4
no rank

4
node3166.members.0.js
1632780
genus
1

1940610
species
1
node3167.members.0.js

species
1
node3168.members.0.js
1868589

no rank
1
2644610

species
1
node3170.members.0.js
2570229

order
71
204441

41295
24
family

204447
genus
1

2233999
species
1
node3174.members.0.js

1263978
genus
2

1263979
species
2

1401328
strain
2
node3177.members.0.js

171436
1
genus

171437
1
species

1110502
strain
1
node3180.members.0.js

genus
1
1231242

2594003
species
1
node3182.members.0.js

13134
genus
2

species
1
84159

342108
strain
1
node3185.members.0.js

1
no rank
2617991

node3187.members.0.js
1
species
1663591

1804663
genus
3

1549855
species
3
node3189.members.0.js

2
genus
1081

34018
2
species

2
node3192.members.0.js
strain
414684

2705399
3
genus

species
1
node3194.members.0.js
2602016

node3195.members.0.js
2
species
2602015

191
9
node3196.members.0.js
2
genus

1
species
192

1
node3198.members.0.js
strain
1064539

2202148
1
node3199.members.0.js
species

2
no rank
2630922

652764
species
1
node3201.members.0.js

node3202.members.0.js
1
species
664962

1226968
node3203.members.0.js
1
species

528244
node3204.members.0.js
2
species

1
family
433
47
node3205.members.0.js

1434011
5
node3206.members.0.js
1
genus

436
2
species

714995
strain
2
node3208.members.0.js

265959
species
1
node3209.members.0.js

1
node3210.members.0.js
species
28448

1223423
genus
1

1
node3212.members.0.js
species
661191

genus
1
153497

node3214.members.0.js
1
species
153496

genus
30
node3215.members.0.js
434

41293
1
no rank

1909293
node3217.members.0.js
1
species

2603324
genus
1

species
1
node3219.members.0.js
1969806

1602345
genus
1

1
node3221.members.0.js
species
1510841

1079922
1
genus

2630218
no rank
node3223.members.0.js
1

1654741
1
genus

no rank
1
2644098

species
node3226.members.0.js
1
2697033

1
genus
125216
4
node3227.members.0.js

257708
2
node3228.members.0.js
species

no rank
1
2617492

2018065
node3230.members.0.js
1
species

42
order
204458

42
node3232.members.0.js
76892
family
3

genus
1
76890

78587
1
node3234.members.0.js
species

4
genus
41275
node3235.members.0.js
13

588932
2
node3236.members.0.js
species

species
2
74313

633149
node3238.members.0.js
2
strain

2622653
5
node3239.members.0.js
4
no rank

species
node3240.members.0.js
1
2591463

1
genus
20

284016
species
1

450851
strain
node3243.members.0.js
1

6
genus
75
node3244.members.0.js
24

155892
1
node3245.members.0.js
species

1
node3246.members.0.js
species
2010972

2648921
12
no rank

366602
node3248.members.0.js
1
species

11
node3249.members.0.js
species
69665

69395
node3250.members.0.js
1
species

1679497
node3251.members.0.js
2
species

88688
species
node3252.members.0.js
1

order
1
1191478

1
family
1191479

genus
1
162171

1124597
1
species

node3257.members.0.js
1
strain
156889

5
order
54526

1655514
family
5

no rank
2
1655516

species
2
node3261.members.0.js
859653

198251
3
genus

2647897
3
no rank

1002672
1
node3264.members.0.js
species

2
node3265.members.0.js
species
1388755

1921002
1
order

44746
family
1

1
no rank
204454

node3269.members.0.js
1
species
86106

74
order
356
node3270.members.0.js
727

family
2
2723775

2723776
2
genus

2
node3273.members.0.js
species
2528642

node3274.members.0.js
328
41294
family
11

911
1
genus

1
species
912

323097
node3277.members.0.js
1
strain

1395974
1
genus

no rank
node3279.members.0.js
1
2631404

node3280.members.0.js
284
85413
genus
27

node3281.members.0.js
244
2653178
no rank
51

1867715
node3282.members.0.js
26
species

1842539
species
node3283.members.0.js
12

42
node3284.members.0.js
species
2015316

node3285.members.0.js
107
species
2599640

1792307
species
node3286.members.0.js
6

1526658
13
node3287.members.0.js
species

374
26
node3288.members.0.js
7
genus

1
node3289.members.0.js
species
931866

375
species
1
node3290.members.0.js

node3291.members.0.js
2
species
244734

species
node3292.members.0.js
1
1355477

species
node3293.members.0.js
2
1325107

1325095
1
node3294.members.0.js
species

node3295.members.0.js
6
2631580
no rank
2

2493093
1
node3296.members.0.js
species

1223566
species
1
node3297.members.0.js

167468
species
node3298.members.0.js
2

1325115
species
node3299.members.0.js
1

1437360
species
node3300.members.0.js
3

722472
species
node3301.members.0.js
1

3
genus
1073

species
3
1076

strain
node3304.members.0.js
1
316058

316055
node3305.members.0.js
2
strain

81426
no rank
2

709797
node3307.members.0.js
2
species

772
family
18

genus
6
node3309.members.0.js
18
773

388640
species
1

1094489
1
node3311.members.0.js
strain

1
species
56426

696125
1
node3313.members.0.js
strain

species
node3314.members.0.js
3
85701

species
node3315.members.0.js
2
803

1686310
1
node3316.members.0.js
species

3
node3317.members.0.js
species
807

33047
species
node3318.members.0.js
1

family
3
255475

node3320.members.0.js
3
293088
genus
1

293089
species
2

1122214
strain
node3322.members.0.js
2

4
family
335928

279
2
genus

species
2
280

78245
node3326.members.0.js
2
strain

204476
1
genus

376174
1
node3328.members.0.js
species

6
1
genus

1
species
7

438753
node3331.members.0.js
1
strain

2036754
3
family

28209
node3333.members.0.js
3
1
genus

2638111
no rank
2

node3335.members.0.js
2
species
1702325

20
family
45401

2
genus
46913

1
node3338.members.0.js
species
400770

1
no rank
196773

node3340.members.0.js
1
species
1736675

1
genus
1827478

1447062
1
node3342.members.0.js
species

81
15
genus

species
15
53399

582899
1
node3345.members.0.js
strain

14
node3346.members.0.js
strain
670307

59282
2
genus

node3348.members.0.js
1
species
2233851

1079
species
node3349.members.0.js
1

1
family
45404

genus
1
1156568

569860
species
1
node3352.members.0.js

family
1
node3353.members.0.js
35
69277

genus
2
31988

no rank
2
2644704

species
2
node3356.members.0.js
374606

2712688
genus
1

species
node3358.members.0.js
1
2712698

genus
4
1649463

no rank
4
2647570

species
node3361.members.0.js
4
2305987

28100
5
genus

1867719
species
5
node3363.members.0.js

68287
node3364.members.0.js
22
5
genus

species
1
71433

1082933
strain
1
node3366.members.0.js

536018
species
1

strain
1
node3368.members.0.js
754035

593909
species
1

strain
1
node3370.members.0.js
536019

species
node3371.members.0.js
5
1295366

species
node3372.members.0.js
1
1670800

2066070
1
node3373.members.0.js
species

no rank
7
325217

node3375.members.0.js
1
species
2493673

node3376.members.0.js
2
species
2082387

2493669
node3377.members.0.js
1
species

2493668
species
2
node3378.members.0.js

1
node3379.members.0.js
species
2493678

119045
node3380.members.0.js
34
2
family

genus
2
186650

species
node3382.members.0.js
1
1882682

2617746
1
no rank

node3384.members.0.js
1
species
2082949

16
genus
407
node3385.members.0.js
29

species
node3386.members.0.js
1
418223

node3387.members.0.js
2
species
2202827

no rank
1
node3388.members.0.js
4
2615210

2202828
species
node3389.members.0.js
1

925818
species
node3390.members.0.js
1

species
1
node3391.members.0.js
426117

species
2
114616

460265
strain
node3393.members.0.js
2

270351
species
node3394.members.0.js
3

31998
1
species

426355
strain
1
node3396.members.0.js

genus
1
2282523

223967
species
1
node3398.members.0.js

family
5
31993

133
node3400.members.0.js
2
1
genus

species
1
node3401.members.0.js
173366

1
genus
425
node3402.members.0.js
3

1
no rank
2624500

2699395
species
node3404.members.0.js
1

426
1
species

595536
node3406.members.0.js
1
strain

34
family
82115
187
node3407.members.0.js

node3408.members.0.js
56
227290
no rank
9

29
node3409.members.0.js
379
genus
8

1
species
384
node3410.members.0.js
4

no rank
1
386

395491
1
node3412.members.0.js
strain

1
no rank
387
2
node3413.members.0.js

strain
node3414.members.0.js
1
216596

2
no rank
2613769
12
node3415.members.0.js

2020312
1
node3416.members.0.js
species

2048897
node3417.members.0.js
2
species

1
node3418.members.0.js
species
1869170

2
node3419.members.0.js
species
2028343

node3420.members.0.js
2
species
1571470

species
1
node3421.members.0.js
2603277

species
node3422.members.0.js
1
1981173

species
node3423.members.0.js
1
396

240521
node3424.members.0.js
1
species

348824
1
node3425.members.0.js
species

node3426.members.0.js
1
species
379684

56730
node3427.members.0.js
1
species

16
genus
357

373
1
species

311402
1
node3430.members.0.js
strain

2632611
no rank
1

node3432.members.0.js
1
species
2580515

160699
1
node3433.members.0.js
species

species group
13
1183400

node3435.members.0.js
13
species
358

1525371
genus
2

399
species
1

1
no rank
323655

1
node3439.members.0.js
strain
1028800

2629175
no rank
1

1825976
1
node3441.members.0.js
species

2
no rank
227292
50
node3442.members.0.js

106591
genus
18

716925
1
species

716928
node3445.members.0.js
1
strain

species
12
16
node3446.members.0.js
106592

strain
node3447.members.0.js
4
1416753

1752398
node3448.members.0.js
1
species

30
node3449.members.0.js
28105
genus
2

663276
species group
2

380
node3451.members.0.js
2
1
species

1
node3452.members.0.js
strain
394

25
no rank
2613772

1842534
25
node3454.members.0.js
species

382
node3455.members.0.js
1
species

2661800
1
genus

2341112
1
node3457.members.0.js
species

34019
genus
1

1273132
species
1

1215343
strain
node3460.members.0.js
1

323620
genus
45

no rank
45
2643062

879274
species
node3463.members.0.js
45

119042
no rank
8

2
genus
573657

573658
node3466.members.0.js
2
species

169055
2
genus

2
no rank
2623691

species
2
node3469.members.0.js
2712222

1734920
4
genus

1235591
node3471.members.0.js
4
species

1
family
655351

1
genus
1406135

2624130
1
no rank

2304600
species
node3475.members.0.js
1

118882
4
family

1
genus
528
3
node3477.members.0.js

529
species
2

439375
strain
node3479.members.0.js
2

1
genus
234

235
species
node3481.members.0.js
1

138
node3482.members.0.js
204457
order
4

335929
56
family

1111
2
genus

2683265
2
no rank

node3486.members.0.js
2
species
2547601

genus
52
361177

4
no rank
2614945

species
4
node3489.members.0.js
2060312

361183
node3490.members.0.js
48
species

2
genus
1041

1
no rank
2633097

species
1
node3493.members.0.js
2011159

502682
node3494.members.0.js
1
species

41297
78
node3495.members.0.js
3
family

1
genus
335405

335406
species
node3497.members.0.js
1

node3498.members.0.js
25
165697
genus
7

117207
1
species

strain
node3500.members.0.js
1
317655

33050
species
3
node3501.members.0.js

species
node3502.members.0.js
1
2054227

13
no rank
2614943

species
node3504.members.0.js
5
1357916

species
3
node3505.members.0.js
1874061

node3506.members.0.js
2
species
1914525

2
node3507.members.0.js
species
1913578

species
node3508.members.0.js
1
292913

genus
1
node3509.members.0.js
3
165696

species
1
169176

1219035
node3511.members.0.js
1
strain

2644732
no rank
1

702113
1
node3513.members.0.js
species

165695
4
genus

species
1
336203

1208342
strain
1
node3516.members.0.js

species
node3517.members.0.js
2
120107

1
species
46429

690566
strain
1
node3519.members.0.js

genus
1
1434046

2614947
1
no rank

species
node3522.members.0.js
1
1806885

7
genus
13687
41
node3523.members.0.js

species
node3524.members.0.js
1
1560345

node3525.members.0.js
3
species
13689

1609977
1
node3526.members.0.js
species

196159
17
no rank

2681549
species
2
node3528.members.0.js

2319844
species
node3529.members.0.js
8

1523415
species
2
node3530.members.0.js

1961362
1
node3531.members.0.js
species

2219696
1
node3532.members.0.js
species

1381597
node3533.members.0.js
1
species

species
node3534.members.0.js
1
1390395

2698679
species
node3535.members.0.js
1

363835
node3536.members.0.js
1
species

152682
11
node3537.members.0.js
9
species

621456
2
node3538.members.0.js
strain

order
16
766

family
7
775

33988
tribe
6

69474
1
genus

784
species
node3543.members.0.js
1

5
genus
780

114292
1
species group

785
1
node3546.members.0.js
species

4
species group
1129742

33990
species
3
node3548.members.0.js

788
1
node3549.members.0.js
species

genus
1
2115980

2115978
node3551.members.0.js
1
species

2603433
family
2

2603321
genus
2

node3554.members.0.js
2
species
2163644

family
7
942

genus
1
768

106179
species group
1

species
node3558.members.0.js
1
948

943
genus
4

4
species group
106178

species
2
4
node3561.members.0.js
779

302409
strain
2
node3562.members.0.js

952
tribe
2

genus
2
node3564.members.0.js
953

class
1
1807140

1
order
225057

225058
1
family

genus
1
119977

920
species
1

243159
node3570.members.0.js
1
strain

class
27
1553900

2024973
order
8

3
no rank
2493640

node3574.members.0.js
3
species
2493639

5
family
2024974

genus
5
2024975

node3577.members.0.js
5
species
1915309

order
10
213481

213483
10
family

genus
10
958

2
species
453816

1184267
node3582.members.0.js
2
strain

959
species
8

765869
strain
node3584.members.0.js
8

9
order
2024979

1652132
9
family

1652133
9
genus

8
no rank
2639665

8
node3589.members.0.js
species
2109558

97084
1
node3590.members.0.js
species

62
class
1236
2069
node3591.members.0.js

135625
order
43

43
node3593.members.0.js
712
family
1

416916
genus
1

739
species
1

888057
1
node3596.members.0.js
strain

1
genus
75984

75985
species
node3598.members.0.js
1

3
genus
713

node3600.members.0.js
3
species
51049

724
node3601.members.0.js
24
5
genus

node3602.members.0.js
1
species
726

node3603.members.0.js
1
species
249188

species
node3604.members.0.js
1
730

729
species
16
node3605.members.0.js

genus
7
476528

species
node3607.members.0.js
7
47735

6
genus
745

node3609.members.0.js
1
species
754

747
species
5
node3610.members.0.js

order
94
135624

family
1
94
node3612.members.0.js
84642

129577
genus
1

no rank
1
2636315

511062
node3615.members.0.js
1
species

43947
genus
1

1
species
43948

595494
1
node3618.members.0.js
strain

91
node3619.members.0.js
642
genus
60

948519
species
1
node3620.members.0.js

node3621.members.0.js
1
species
196024

645
species
1
node3622.members.0.js

652
1
node3623.members.0.js
species

no rank
3
257493

1636609
2
node3625.members.0.js
species

1636606
1
node3626.members.0.js
species

654
2
node3627.members.0.js
species

node3628.members.0.js
22
644
species
21

196023
node3629.members.0.js
1
subspecies

135613
11
order

72276
family
2

1
genus
106633

node3633.members.0.js
1
species
106634

1335745
1
genus

1335746
species
1

strain
1
node3636.members.0.js
1260251

1046
9
family

genus
3
67575

no rank
3
115860

2498451
species
node3640.members.0.js
1

species
node3641.members.0.js
2
2545632

2
no rank
82569

species
2
node3643.members.0.js
1978339

3
genus
1227

3
node3645.members.0.js
species
1814290

genus
1
53392

1166950
species
node3647.members.0.js
1

72273
order
40

family
31
34064

genus
18
node3650.members.0.js
31
262

species
node3651.members.0.js
1
2249302

263
species
node3652.members.0.js
1

species
1
node3653.members.0.js
573569

species
2
node3654.members.0.js
2007306

4
no rank
2610885

4
node3656.members.0.js
species
1547445

573570
1
node3657.members.0.js
species

species
1
node3658.members.0.js
28110

species
1
954

1086726
1
node3660.members.0.js
strain

1542390
species
node3661.members.0.js
1

135616
8
family

1
genus
933

147268
1
species

717773
strain
1
node3665.members.0.js

genus
6
28884

39765
species
6

317025
node3668.members.0.js
6
strain

1237
1
genus

1238
1
node3670.members.0.js
species

family
1
135617

1
genus
1021

species
1
node3673.members.0.js
288004

2
order
1775403

family
2
568386

1
genus
413435

2637139
1
no rank

1
node3678.members.0.js
species
2303331

1861863
1
genus

2621506
no rank
1

1
node3681.members.0.js
species
2698684

118969
order
39

1
family
118968

776
1
genus

node3685.members.0.js
1
species
2054173

family
38
444

461
genus
5

5
node3688.members.0.js
species
463

33
genus
445

2005262
species
node3690.members.0.js
4

node3691.members.0.js
4
species
446

6
species
29423

1268635
strain
node3693.members.0.js
6

45065
node3694.members.0.js
1
species

1867846
9
node3695.members.0.js
species

452
species
1
node3696.members.0.js

1
node3697.members.0.js
species
454

449
species
2
node3698.members.0.js

450
species
5
node3699.members.0.js

order
13
1240482

1240483
family
13

genus
3
1193503

3
node3703.members.0.js
species
1196095

1335631
genus
10

1267021
species
10
node3705.members.0.js

159
order
135614

32033
node3707.members.0.js
153
2
family

genus
6
node3708.members.0.js
13
40323

5
node3709.members.0.js
995085
species group
2

species
1
2
node3710.members.0.js
40324

node3711.members.0.js
1
strain
522373

node3712.members.0.js
1
species
2072413

196198
2
node3713.members.0.js
1
no rank

node3714.members.0.js
1
species
2005046

68
genus
6

species
node3716.members.0.js
2
435897

69
4
node3717.members.0.js
species

genus
1
490567

370777
species
1
node3719.members.0.js

1
genus
338
node3720.members.0.js
130

343
species
1

no rank
1
134875

1261556
strain
1
node3723.members.0.js

56448
node3724.members.0.js
1
species

1985254
1
species

1
no rank
92828

1437877
strain
node3727.members.0.js
1

species
1
347

64187
node3729.members.0.js
1
no rank

456327
119
species

subspecies
119
359387

1365647
119
node3732.members.0.js
strain

56450
species
1

1219375
strain
node3734.members.0.js
1

species
node3735.members.0.js
1
56455

node3736.members.0.js
1
species
56458

species group
3
643453

species
1
node3738.members.0.js
3
346

no rank
2
node3739.members.0.js
473421

83618
genus
1

1
species
314722

743721
1
node3742.members.0.js
strain

1775411
6
family

242605
1
genus

242606
1
species

strain
node3746.members.0.js
1
1440763

2233801
genus
4

2021234
4
node3748.members.0.js
species

1
genus
75309

node3750.members.0.js
1
species
582702

1
order
1934945

family
1
1934946

1
genus
1934947

1810504
species
1
node3754.members.0.js

135618
1
order

403
1
family

genus
1
416

1
node3758.members.0.js
species
1538553

node3759.members.0.js
250
135622
order
47

267890
family
28

genus
4
node3761.members.0.js
25
22

1965282
node3762.members.0.js
1
species

species
2
70863

211586
node3764.members.0.js
2
strain

5
node3765.members.0.js
species
150120

38313
species
node3766.members.0.js
1

5
species
56812

318167
5
node3768.members.0.js
strain

species
3
404011

3
node3770.members.0.js
strain
225849

196818
no rank
4

node3772.members.0.js
1
species
1930557

node3773.members.0.js
1
species
2029986

2575361
species
node3774.members.0.js
1

node3775.members.0.js
1
species
2487742

3
genus
2547964

1
node3777.members.0.js
species
342950

2547970
2
node3778.members.0.js
species

14
family
267889

node3780.members.0.js
3
28228
genus
2

1
no rank
196834

2161872
species
node3782.members.0.js
1

11
genus
1518149

2614972
no rank
1

species
1
node3785.members.0.js
2552945

1763536
node3786.members.0.js
10
species

4
family
267891

4
genus
58050

node3789.members.0.js
4
species
80854

38
family
267888

10
genus
53246
38
node3791.members.0.js

43657
2
node3792.members.0.js
species

394751
species
4

strain
node3794.members.0.js
4
1117313

1
node3795.members.0.js
species
314281

288
1
species

342610
node3797.members.0.js
1
strain

species
1
node3798.members.0.js
621376

10
no rank
194690

1761891
species
node3800.members.0.js
1

species
6
node3801.members.0.js
2583375

1709477
3
node3802.members.0.js
species

43662
node3803.members.0.js
9
species

family
1
node3804.members.0.js
119
72275

17
genus
288793

no rank
1
2645119

2572577
1
node3807.members.0.js
species

species
16
node3808.members.0.js
914153

genus
11
2742

1420917
node3810.members.0.js
9
species

1
no rank
83889

species
1
node3812.members.0.js
1749259

species
node3813.members.0.js
1
2743

genus
2
89404

2
no rank
2618684

2
node3816.members.0.js
species
2489595

genus
9
88
node3817.members.0.js
226

314275
species
49
node3818.members.0.js

species
8
9
node3819.members.0.js
28108

strain
1
node3820.members.0.js
1004785

589873
node3821.members.0.js
12
species

no rank
9
2614992

2652380
species
4
node3823.members.0.js

species
3
node3824.members.0.js
2058133

species
node3825.members.0.js
2
2267264

1692040
2
order

1692041
family
2

1744881
genus
1

1620215
species
node3829.members.0.js
1

1692042
1
genus

node3831.members.0.js
1
species
1675686

1706369
order
13

2
family
1706373

48073
genus
2

252514
node3835.members.0.js
1
species

1
no rank
2619833

1516059
species
node3837.members.0.js
1

1706371
family
11

2
genus
10

2624793
no rank
2

1945512
node3841.members.0.js
1
species

1
node3842.members.0.js
species
454662

2036021
3
genus

3
node3844.members.0.js
species
1737490

6
genus
447467

447471
species
6

1117647
node3847.members.0.js
6
strain

order
61
135619

family
26
135620

48075
genus
1

1821621
node3851.members.0.js
1
species

15
genus
28253

species
7
119864

717774
strain
node3854.members.0.js
7

196814
no rank
8

2071621
species
4
node3856.members.0.js

400668
4
node3857.members.0.js
species

1
genus
267849

no rank
1
2614692

species
node3860.members.0.js
1
2614693

9
genus
187492

187493
node3862.members.0.js
9
species

439484
1
no rank

2683272
1
node3864.members.0.js
species

28256
family
34

1
genus
1897649

species
1
node3867.members.0.js
1883414

genus
2
2745

2609666
node3869.members.0.js
2
1
no rank

1
node3870.members.0.js
species
1504981

114403
no rank
29

1495768
genus
28

species
28
node3873.members.0.js
1495769

genus
1
235572

91844
species
node3875.members.0.js
1

1
genus
376488

1
node3877.members.0.js
species
376489

404432
1
genus

1771309
node3879.members.0.js
1
species

node3880.members.0.js
366
91347
order
10

family
28
1903414

genus
14
node3882.members.0.js
18
586

1
node3883.members.0.js
species
588

1
species
516075

1141660
strain
1
node3885.members.0.js

587
node3886.members.0.js
2
species

genus
3
581

582
3
node3888.members.0.js
species

1
genus
583
4
node3889.members.0.js

species
1
node3890.members.0.js
584

585
node3891.members.0.js
1
species

1
no rank
257482

2697019
species
1
node3893.members.0.js

637
3
genus

638
species
2
node3895.members.0.js

species
1
node3896.members.0.js
235559

1903410
family
10

1
genus
84565

species
1
63612

343509
node3900.members.0.js
1
strain

204037
6
genus

species
1
node3902.members.0.js
2
204038

node3903.members.0.js
1
strain
198628

556
species
1

1223569
node3905.members.0.js
1
strain

1089444
1
node3906.members.0.js
species

species
node3907.members.0.js
2
204039

genus
2
122277

29471
species
1

1
node3910.members.0.js
strain
218491

1
node3911.members.0.js
species
180957

genus
1
1082702

species
node3913.members.0.js
1
1082704

1903412
9
family

568
genus
9

569
9
node3916.members.0.js
species

19
family
1903411

34037
1
genus

no rank
1
2635087

species
node3920.members.0.js
1
1805933

5
genus
613
node3921.members.0.js
14

614
species
node3922.members.0.js
1

2
node3923.members.0.js
species
615

species
6
138074

568817
node3925.members.0.js
6
strain

629
4
genus

29483
1
species

strain
1
node3928.members.0.js
1453495

node3929.members.0.js
1
species
2339259

29486
species
node3930.members.0.js
1

935293
species
1
node3931.members.0.js

45
family
1903409

21
genus
32199

1
species
9
node3934.members.0.js
21

1265350
forma specialis
node3935.members.0.js
1

forma specialis
1
node3936.members.0.js
98804

forma specialis
1
node3937.members.0.js
1241833

571430
forma specialis
1
node3938.members.0.js

98794
2
node3939.members.0.js
forma specialis

557993
forma specialis
node3940.members.0.js
1

forma specialis
1
node3941.members.0.js
2518980

118101
2
node3942.members.0.js
forma specialis

135842
1
forma specialis

224915
node3944.members.0.js
1
strain

forma specialis
4
node3945.members.0.js
571428

2173854
node3946.members.0.js
1
forma specialis

forma specialis
1
node3947.members.0.js
255719

forma specialis
node3948.members.0.js
1
1241836

forma specialis
1
node3949.members.0.js
118103

forma specialis
node3950.members.0.js
1
98795

1
genus
2100764

1615494
species
node3952.members.0.js
1

19
genus
53335

66269
species
node3954.members.0.js
1

2
node3955.members.0.js
species
470934

node3956.members.0.js
1
species
1235990

1076550
species
2
node3957.members.0.js

2630326
no rank
12

1484158
species
1
node3959.members.0.js

1
node3960.members.0.js
species
2490851

species
10
node3961.members.0.js
592316

59814
node3962.members.0.js
1
species

genus
4
551

1922217
species
node3964.members.0.js
4

245
node3965.members.0.js
543
family
43

1
no rank
36866

891974
1
node3967.members.0.js
species

2
genus
561
node3968.members.0.js
42

node3969.members.0.js
1
species
208962

1
node3970.members.0.js
species
564

37
species
562
38
node3971.members.0.js

serotype
node3972.members.0.js
1
1446746

node3973.members.0.js
6
genus
547

genus
1
node3974.members.0.js
2
413496

1
species
413502

strain
node3976.members.0.js
1
693216

590
3
genus

1
species
28901
3
node3978.members.0.js

node3979.members.0.js
2
59201
subspecies
1

no rank
1
1242084

1242106
1
node3981.members.0.js
strain

9
node3982.members.0.js
544
genus
1

1344959
node3983.members.0.js
8
3
species group

1
node3984.members.0.js
species
133448

57706
species
node3985.members.0.js
3

546
1
node3986.members.0.js
species

1
genus
83654
2
node3987.members.0.js

2627398
1
no rank

node3989.members.0.js
1
species
2282309

191675
8
no rank

clade
8
84563

84564
clade
6

203804
6
genus

251542
1
species

strain
node3995.members.0.js
1
1240471

101534
species
1

strain
node3997.members.0.js
1
291272

711328
no rank
1

species
node3999.members.0.js
1
1505597

2
species
251535

2
node4001.members.0.js
strain
859654

203907
1
node4002.members.0.js
species

472825
1
genus

species
1
node4004.members.0.js
472834

1
genus
1906660

species
node4006.members.0.js
1
1778264

401618
4
genus

species
3
node4008.members.0.js
401619

species
1
node4009.members.0.js
428411

1
genus
1330547

2632876
no rank
1

node4012.members.0.js
1
species
2492396

genus
114
124
node4013.members.0.js
570

species
5
node4014.members.0.js
548

571
species
node4015.members.0.js
1

2153354
species
1
node4016.members.0.js

species
1
node4017.members.0.js
1134687

573
species
node4018.members.0.js
2

no rank
3
118884

745410
genus
1

2291597
species
node4021.members.0.js
1

2
clade
32036

1
genus
2732587

node4024.members.0.js
1
species
412965

113267
species
1

forma specialis
node4026.members.0.js
1
1303921

135623
89
order

641
family
89

511678
genus
2

668
2
species

388396
strain
2
node4031.members.0.js

1
genus
188143

1
node4033.members.0.js
species
1927128

node4034.members.0.js
86
662
genus
52

28173
1
node4035.members.0.js
species

1481923
species
1
node4036.members.0.js

717610
species group
5

670
species
node4038.members.0.js
4

696485
species
1
node4039.members.0.js

212663
6
species

node4041.members.0.js
6
strain
575788

species
node4042.members.0.js
1
672

6
node4043.members.0.js
species
1074311

node4044.members.0.js
2
species
674

2
node4045.members.0.js
species
687

1435069
species
1
node4046.members.0.js

species
2
node4047.members.0.js
190893

species
1
node4048.members.0.js
2572923

species
node4049.members.0.js
2
676

29494
3
node4050.members.0.js
species

246167
species
1

1
node4052.members.0.js
strain
1191300

820
node4053.members.0.js
72274
order
1

family
2
244
node4054.members.0.js
135621

48
genus
286
237
node4055.members.0.js

136843
123
species group

node4057.members.0.js
1
species
380021

species
node4058.members.0.js
1
76758

294
node4059.members.0.js
2
species

47878
species
5
node4060.members.0.js

114
node4061.members.0.js
species
29442

219572
species
node4062.members.0.js
1

species group
2
6
node4063.members.0.js
136849

1206777
1
node4064.members.0.js
species

species
node4065.members.0.js
1
33069

species subgroup
2
251695

1
species
317
node4067.members.0.js
2

1357289
1
node4068.members.0.js
strain

136842
4
species group

587753
species
node4070.members.0.js
2

species
node4071.members.0.js
1
296

node4072.members.0.js
1
species
86185

1
node4073.members.0.js
species
395598

472181
1
node4074.members.0.js
species

1245526
species
1
node4075.members.0.js

species group
11
136841

627141
1
species subgroup

species
1
node4078.members.0.js
46680

3
species
300
node4079.members.0.js
5

399739
1
node4080.members.0.js
strain

1225174
node4081.members.0.js
1
strain

node4082.members.0.js
1
species
53408

node4083.members.0.js
4
species
287

104087
3
node4084.members.0.js
species

9
species group
136846

74829
species
2

node4087.members.0.js
2
strain
1123016

species subgroup
2
578833

316
2
node4089.members.0.js
species

species
node4090.members.0.js
5
271420

species group
3
14
node4091.members.0.js
136845

node4092.members.0.js
11
303
species
7

1
node4093.members.0.js
strain
1211579

1331671
strain
node4094.members.0.js
3

species
node4095.members.0.js
1
65741

node4096.members.0.js
1
species
1615674

216142
node4097.members.0.js
1
species

196821
12
no rank

1294143
1
node4099.members.0.js
species

1
node4100.members.0.js
species
1259844

node4101.members.0.js
1
species
2320867

2518644
1
node4102.members.0.js
species

species
node4103.members.0.js
1
2505979

1930532
1
node4104.members.0.js
species

2083051
species
1
node4105.members.0.js

1
node4106.members.0.js
species
1573720

1028989
species
node4107.members.0.js
1

species
1
node4108.members.0.js
2018067

species
node4109.members.0.js
1
1981174

species
node4110.members.0.js
1
1573719

1849530
genus
5

1697053
species
node4112.members.0.js
5

6
family
468
575
node4113.members.0.js

469
533
node4114.members.0.js
188
genus

909768
139
node4115.members.0.js
13
species group

471
species
node4116.members.0.js
31

470
node4117.members.0.js
40
39
species

strain
1
node4118.members.0.js
1400867

106654
species
1
node4119.members.0.js

52
species
48296
54
node4120.members.0.js

2
node4121.members.0.js
strain
871585

species
1
node4122.members.0.js
1879050

species
node4123.members.0.js
8
1324350

3
node4124.members.0.js
species
40216

node4125.members.0.js
71
species
108981

1148157
1
species

1
node4127.members.0.js
strain
436717

28090
17
node4128.members.0.js
species

2
node4129.members.0.js
species
2004650

106648
species
3
node4130.members.0.js

1
species
202950

62977
strain
node4132.members.0.js
1

196816
no rank
19

1
node4134.members.0.js
species
2609668

1808001
node4135.members.0.js
1
species

species
node4136.members.0.js
12
2004644

1636603
1
node4137.members.0.js
species

species
node4138.members.0.js
4
1646498

106649
species
3
node4139.members.0.js

species
node4140.members.0.js
75
756892

70348
species
1
node4141.members.0.js

29430
1
node4142.members.0.js
species

genus
7
node4143.members.0.js
20
497

3
species
334543

strain
3
node4145.members.0.js
259536

no rank
10
196806

1028416
species
4
node4147.members.0.js

2203895
species
1
node4148.members.0.js

349106
node4149.members.0.js
1
species

1699621
node4150.members.0.js
4
species

15
genus
475

5
node4152.members.0.js
species
476

34062
node4153.members.0.js
4
species

species
1
node4154.members.0.js
34061

species
2
5
node4155.members.0.js
480

1236608
strain
node4156.members.0.js
3

1
no rank
54393

node4158.members.0.js
1
species
2283318

phylum
53
40117

203693
class
53

order
53
189778

family
53
189779

28261
50
genus

28262
50
species

289376
strain
50
node4165.members.0.js

genus
1
1234

species
1
node4167.members.0.js
1715989

genus
2
node4168.members.0.js
179

2
phylum
200940

2
class
67799

order
2
188710

188711
family
2

1740
genus
1

1
no rank
2638574

species
node4175.members.0.js
1
2234087

genus
1
444090

1653476
node4177.members.0.js
1
species

1930617
3
phylum

3
class
1962850

1962852
order
3

family
3
1962854

genus
3
187144

187145
3
species

strain
node4184.members.0.js
3
880073

phylum
4
74152

641853
4
class

641854
order
4

family
4
641876

423604
genus
4

species
4
423605

strain
node4191.members.0.js
4
445932

48
phylum
203691

203692
48
class

order
6
1643688

family
6
170

6
genus
171

species
1
174

1
node4198.members.0.js
no rank
508536

173
node4199.members.0.js
2
1
species

1
node4200.members.0.js
no rank
214675

species
node4201.members.0.js
1
1137606

172
species
2

145259
no rank
node4203.members.0.js
2

16
order
1643686

143786
16
family

genus
2
node4206.members.0.js
16
29521

84378
species
2

526224
2
node4208.members.0.js
strain

3
species
84377

1045858
strain
3
node4210.members.0.js

species
node4211.members.0.js
1
1287055

3
node4212.members.0.js
159
species
2

strain
node4213.members.0.js
1
1266923

3
species
52584
5
node4214.members.0.js

strain
node4215.members.0.js
2
1161918

136
order
26

11
family
1643685

3
genus
64895
node4218.members.0.js
6

64897
1
species

521010
node4220.members.0.js
1
strain

29519
species
1
node4221.members.0.js

species
node4222.members.0.js
1
1964448

1
genus
138
node4223.members.0.js
5

47466
species
3
node4224.members.0.js

140
species
node4225.members.0.js
1

family
15
137

4
genus
146

252967
species
node4228.members.0.js
4

genus
11
157

167
4
species

node4231.members.0.js
4
strain
869209

species
4
6
node4232.members.0.js
158

999432
strain
1
node4233.members.0.js

999434
node4234.members.0.js
1
strain

1
node4235.members.0.js
species
162

508458
phylum
3

649775
class
3

3
order
649776

649777
family
3

336260
genus
3

336261
species
3

strain
3
node4242.members.0.js
580340

200783
phylum
6

187857
6
class

32069
order
6

family
2
64898

2
genus
168657

1
no rank
2622382
node4248.members.0.js
2

380749
1
node4249.members.0.js
species

4
family
224027

212790
genus
4

2619248
4
no rank

436114
species
4
node4253.members.0.js

superkingdom
99
10239

12
clade
2732004

kingdom
1
2732006

2732009
1
phylum

1
class
2732010

2732011
order
1

1714267
family
1

1714268
genus
1

species
node4262.members.0.js
1
1154689

kingdom
11
2732005

10
phylum
2732007

2732525
3
class

2732527
order
3

family
3
10240

subfamily
2
10284

1
no rank
39747

1
node4270.members.0.js
species
2259792

1
genus
10286

1993631
species
1

1293539
1
node4273.members.0.js
no rank

subfamily
1
10241

genus
1
2005509

2025358
1
node4276.members.0.js
no rank

7
class
2732523

2732554
order
4

549779
4
family

genus
4
315393

no rank
4
2501774

1128151
node4282.members.0.js
3
species

species
node4283.members.0.js
1
1094892

2
order
2732555

10486
family
2

1
subfamily
2017757

1
genus
10491

species
node4288.members.0.js
1
345198

1
no rank
180169

2035708
1
node4290.members.0.js
species

2732524
1
order

10501
1
family

1
no rank
455363

2023057
1
node4294.members.0.js
species

1
phylum
2732008

2732528
class
1

order
1
2732556

1914302
family
1

2268405
no rank
1

species
1
node4300.members.0.js
1557033

family
2
10442

558016
genus
2

1675866
1
node4303.members.0.js
species

1
node4304.members.0.js
species
59376

2
clade
2731342

kingdom
2
2732092

2732416
phylum
1

1
class
2732423

2732538
order
1

1
family
251095

genus
1
104766

species
1
node4312.members.0.js
753670

phylum
1
2732415

class
1
2732421

order
1
2732532

family
1
151341

1891713
1
genus

1891742
1
species

1219896
node4319.members.0.js
1
no rank

1
no rank
12429

no rank
1
2204151

1
no rank
35342

2502017
clade
1

node4324.members.0.js
1
species
2293305

2731341
clade
80

clade
80
2731360

2731618
phylum
80

2731619
class
80

80
order
28883

1
family
10744

196895
1
no rank

1978007
clade
1

no rank
1
2315857

1211417
1
node4334.members.0.js
species

10699
family
71

subfamily
1
1982876

1982898
1
genus

1983107
species
1

no rank
node4339.members.0.js
1
1429905

node4340.members.0.js
1
genus
186764

no rank
59
196894

1
node4342.members.0.js
species
1476886

190478
species
node4343.members.0.js
57

species
1
node4344.members.0.js
396359

1918718
genus
1

1918720
1
species

756282
node4347.members.0.js
1
no rank

1
genus
1922243

1
species
1922246

1500386
1
node4350.members.0.js
no rank

subfamily
1
1910976

1
node4352.members.0.js
genus
1910991

2560146
genus
7

2560502
species
7

1887648
node4355.members.0.js
7
no rank

7
family
10662

2560128
1
genus

2560849
species
1

no rank
node4359.members.0.js
1
2052742

2733124
genus
1

2734131
species
1

1229753
node4362.members.0.js
1
no rank

1
subfamily
1636616

genus
1
1636617

1
node4365.members.0.js
species
722417

no rank
2
196896

148943
node4367.members.0.js
1
species

1007869
node4368.members.0.js
1
species

subfamily
node4369.members.0.js
1
1198136

node4370.members.0.js
1
genus
1915205

2731643
family
1

genus
1
2731966

1
species
2733944

1283079
no rank
node4374.members.0.js
1

family
2
1511852

1110703
no rank
2

2
node4377.members.0.js
species
1546257
